# Supplementary material for: The soil microbiome modulates the sorghum root metabolome and cellular traits with a concomitant reduction of Striga infection
Source: Cell Rep. 2024 Mar 26;43(4):113971. doi: 10.1016/j.celrep.2024.113971 (PMC11063626; doi:10.1016/j.celrep.2024.113971)
Supplement: Document S1. Figures S1–S8 [file mmc1.pdf]

Cell Reports, Volume *43*

Supplemental information

The soil microbiome modulates the sorghum root metabolome and cellular traits with a concomitant reduction of Striga infection

Dorota Kawa, Benjamin Thiombiano, Mahdere Z. Shimels, Tamera Taylor, Aimee Walmsley, Hannah E. Vahldick, Dominika Rybka, Marcio F.A. Leite, Zayan Musa, Alexander Bucksch, Francisco Dini-Andreote, Mario Schilder, Alexander J. Chen, Jiregna Daksa, Desalegn W. Etalo, Taye Tessema, Eiko E. Kuramae, Jos M. Raaijmakers, Harro Bouwmeester, and Siobhan M. Brady

Supplementary Figures

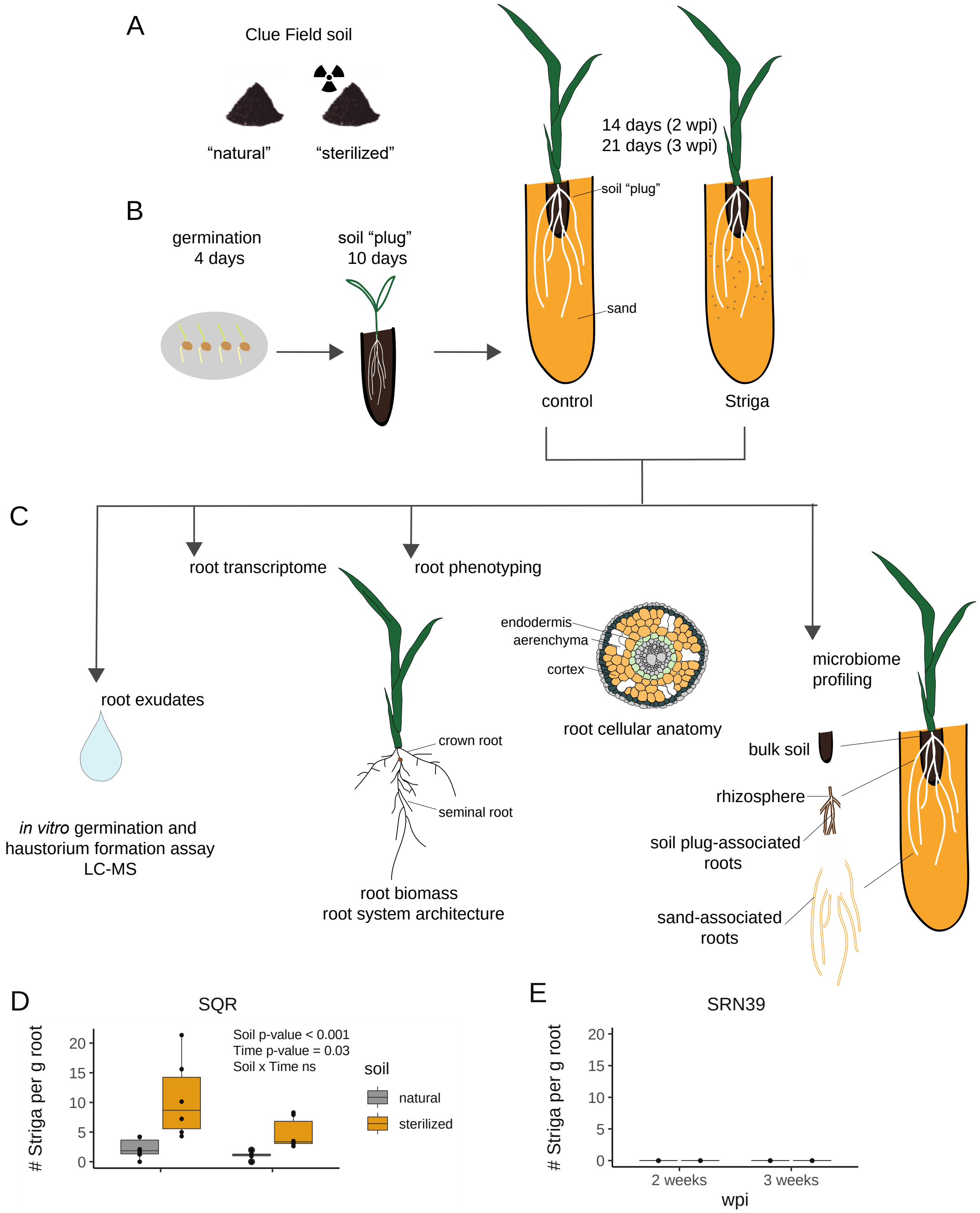

Supplementary Figure 1. Visual description of the methodology used to investigate the mechanisms of Striga infection suppression by soil microbiome. (A) Two batches of Clue Field soil were tested: "natural" and "sterilized", the latter subjected to gamma-irradiation. (B) Sorghum seedlings were germinated and grown for 4 days on moistened filter paper and then (B) transferred to a soil "plug" for 10 days. The seedling, together with the soil "plug" was then transferred to conical tubes filled with sand (control) or sand mixed with Striga seeds. (C) Root tissue and root exudates were collected two and three weeks post-infection (wpi). The root exudates were used for the *in vitro* germination and haustorium formation assay and metabolite analyses. Root phenotyping included quantification of root system architecture and cellular anatomy. Root transcriptomes were profiled with RNAseq. Microbiome profiles were obtained from bulk soil, rhizosphere, soil plug-associated roots (root system part in contact with soil "plug") and sand-associated roots (the root system that was in contact with sand). (D) Number of Striga attachments per gram of fresh root weight of Striga susceptible variety Shanqui Red (SQR) and (E) Striga resistant SRN39 at two and three weeks post-infection (wpi) in natural and sterilized soil. Significance of the differences was assessed with a two-way ANOVA (n=6).

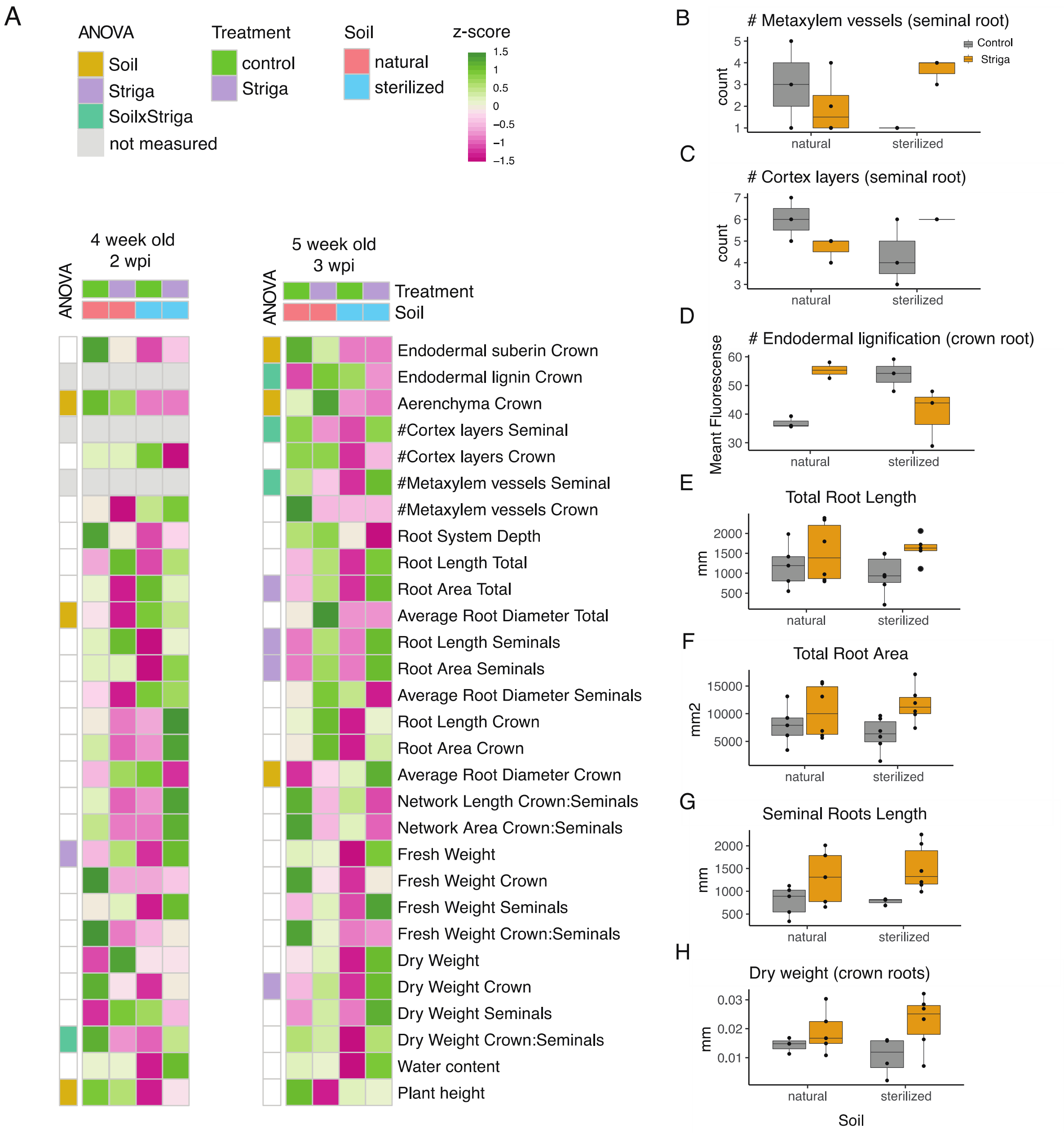

Supplementary Figure 2. Phenotypic characterization of root cellular anatomy, root system architecture and root biomass of Shanqui Red (SQR). (A) Heatmap presents values of each trait scaled across conditions tested. The left panel of each heatmap indicates whether the trait was significantly affected by soil, Striga, and soil by Striga interaction (as identified by a two-way ANOVA). (B) The number of metaxylem vessels and (C) cortex layers in seminal roots, (D) endodermal lignification of crown roots, (E) Length and (F) area of total root system, (G) length of seminal roots and (H) dry biomass of crown roots. Data in B-H are from five-week-old plants (three weeks post-infection). The boxplots denote data spanning from the 25th to the 75th percentile and are centered to the data median. Dots represent individual values.

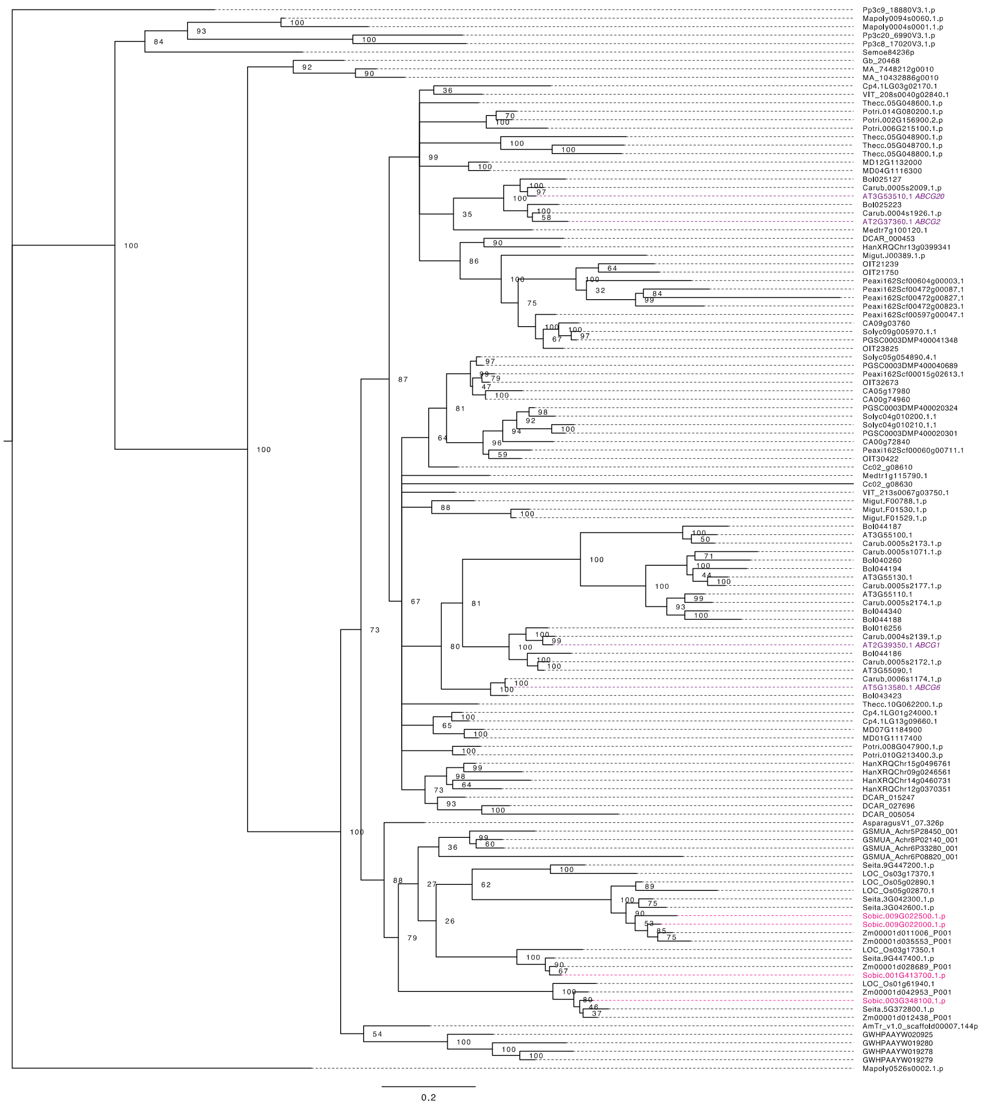

Supplementary Figure 3. Phylogenetic tree of ABCG transporters. Phylogenetic trees generated using protein sequences of several plant species. *Arabidopsis thaliana* genes are highlighted in purple. *S. bicolor* genes are highlighted in pink for reference. AmTr: *Amborella trichopoda*, AT: *Arabidopsis thaliana*, Asparagus: *Asparagus officinalis*, Azfi: *Azolla filiculoides*, Bol: *Brassica oleracea*, Carub: *Capsella rubella*, CA: *Capsicum annuum*, Cc: *Coffea canephora*, Cp: *Cucurbita pepo*, DCAR: *Daucus carota*, Gb: *Ginkgo biloba*, HanXRQ: *Helianthus annuus*, MD: *Malus domestica*, Mapoly: *Marchantia polymorpha*, Medtr: *Medicago truncatula*, Migut: *Mimulus guttatus*, GSMUA: *Musa acuminata*, OIT: *Nicotiana attenuata*, GWHPAAYW: *Nymphaea colorata*, LOC_Os: *Oryza sativa japonica*, Peaxi: *Petunia axillaris,* Pp: *Physcomitrella patens*, MA: *Picea abies*, Potri: *Populus trichocarpa*, Semoe: *Selaginella moellendorffii*, Seita: *Setaria italica,* Solyc: *Solanum lycopersicum*, PGSC: *Solanum tuberosum*, Sobic: *Sorghum bicolor*, Thecc: *Theobroma cacao*, VIT: *Vitis vinifera*, Zm: *Zea mays.*

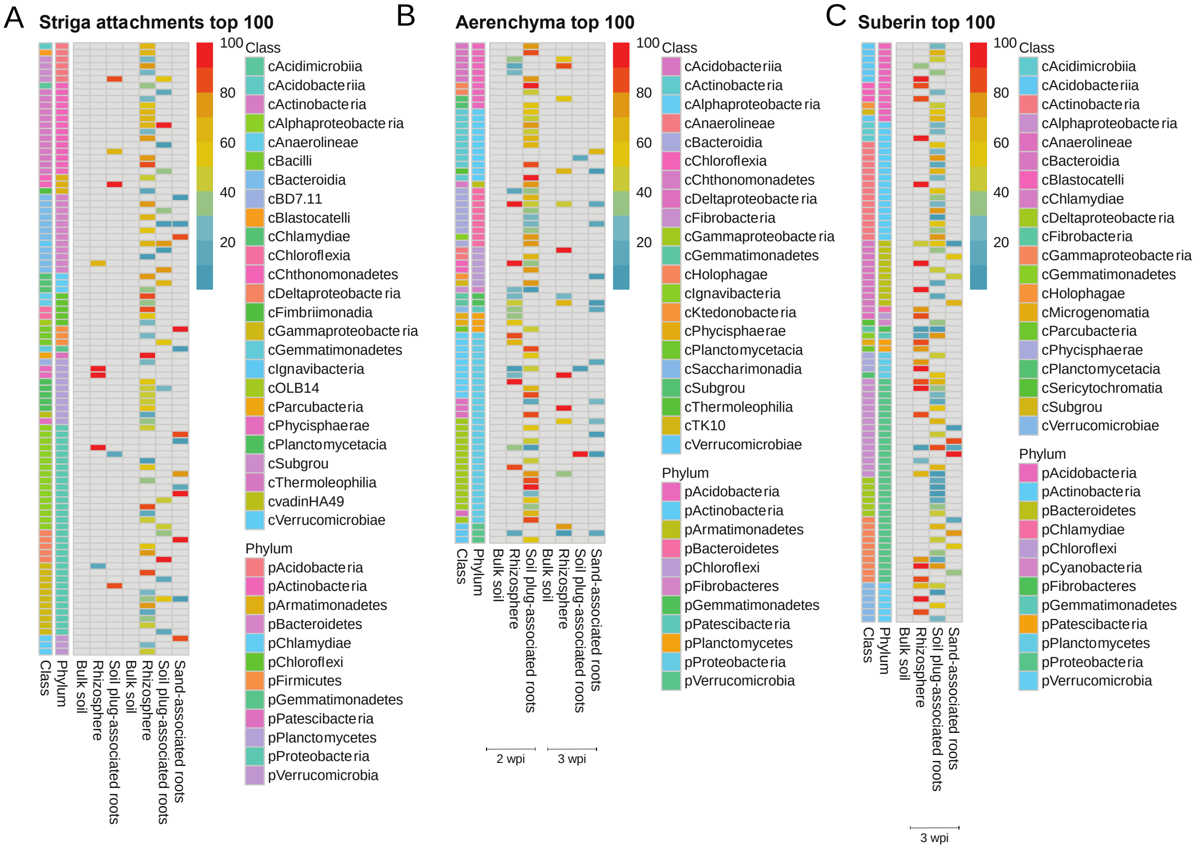

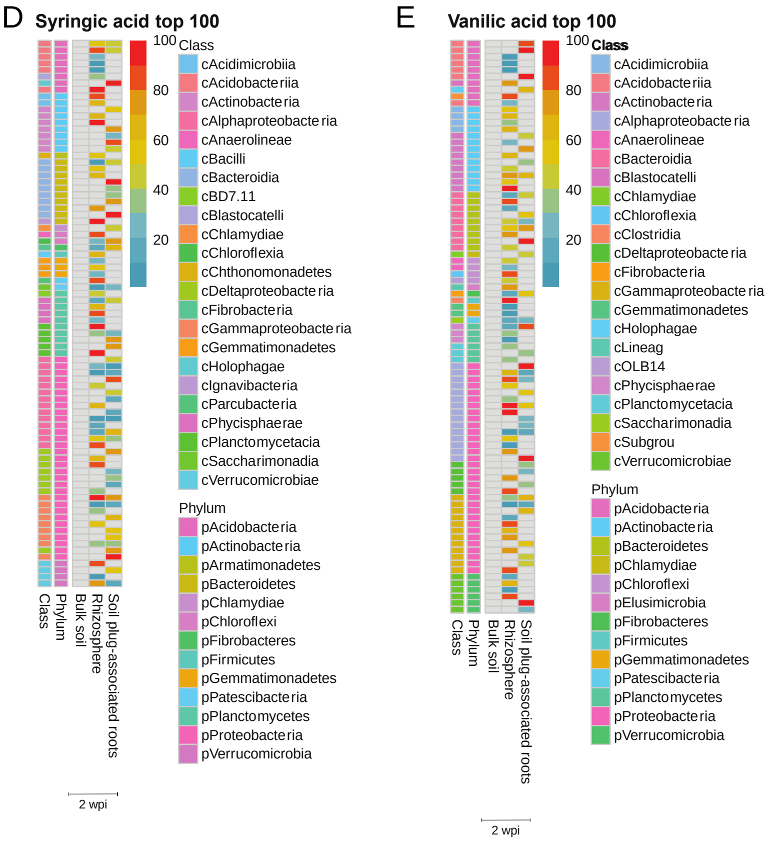

Supplementary Figure 4. Overview of top 100 bacterial taxa predicted to (A) reduce the number of Striga attachments; (B) induce aerenchyma formation; (C) induce endodermal suberization; reduce levels of, (D) syringic acid, (E) vanillic acid. Heatmaps present the rank calculated for each taxa (with blue indicating highest, while red the lowest rank) and the microbial sub-categories where each taxon was found (x-axis). Taxonomic membership at the phylum and class level is indicated with side panel colors.

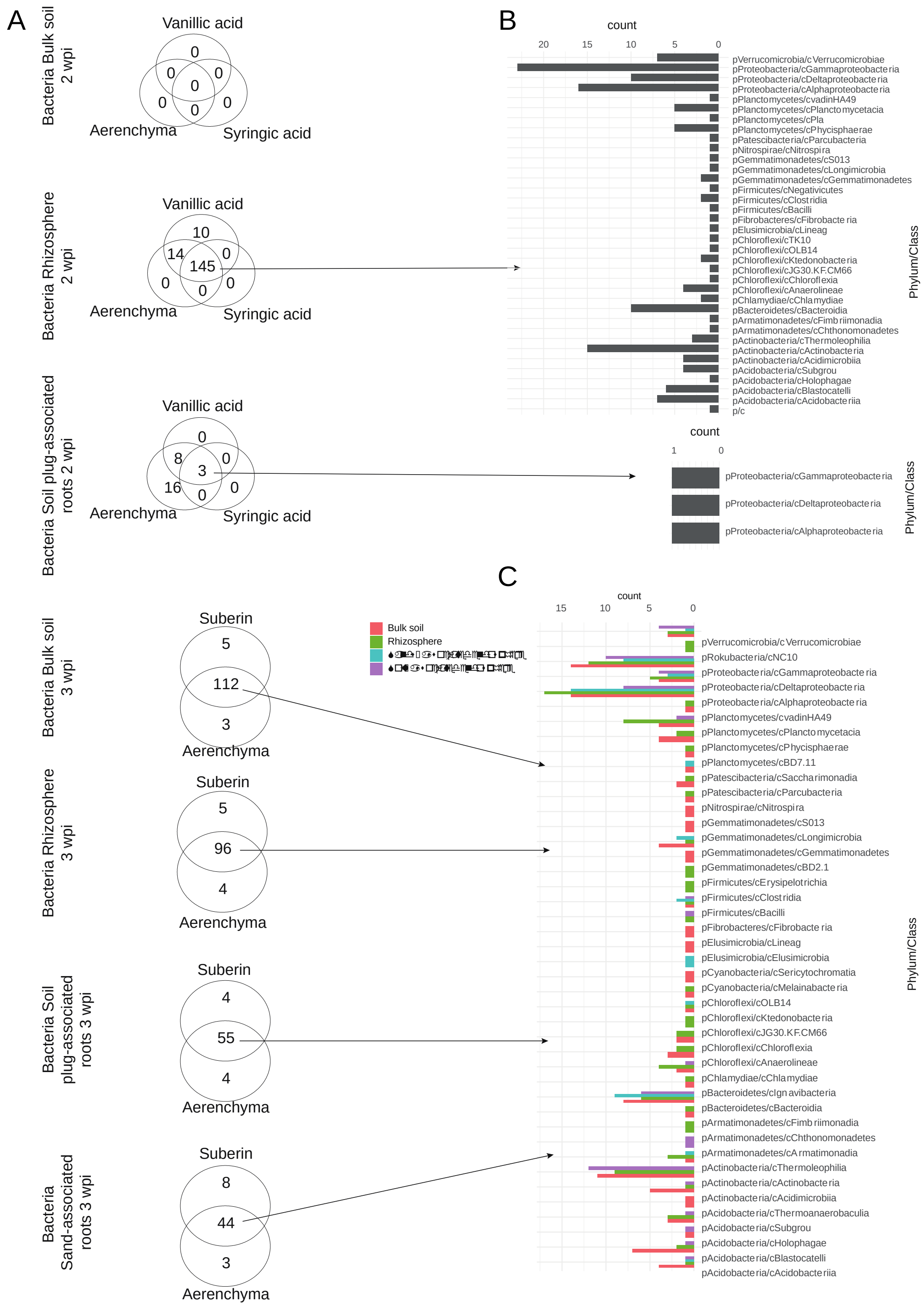

Supplementary Figure 5. Overlap of the number of bacterial taxa predicted to reduce Striga infection via each mechanism of action. (A) Number of bacteria found to reduce Striga infection via each of the mechanisms with the cut-off of residual correlation -0.2 for Striga attachments, syringic acid and vanillic acid levels and 0.2 for aerenchyma proportion and suberin content. Phylogenetic membership of taxa inducing (B) all four modes of Striga suppression in the rhizosphere two weeks post-infection and inducing suberin content and aerenchyma formation across microbial sub-categories three weeks post-infection.

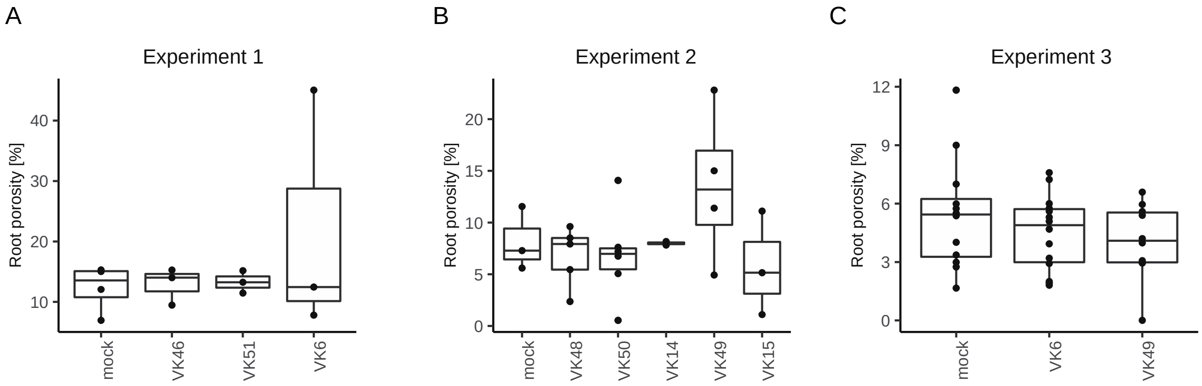

Supplementary Figure 6. Root porosity as a proxy for aerenchyma content of the whole root system (expressed as a proportion of the volume of the whole root system) of plants inoculated with (A) Pseudomonas 1987, 2039 and 2050, (B) Arthrobacter VK48, VK14, VK49, VK49 and Pseudomonas VK50, n = 6. (C) Isolates VK6 and VK49 were retested with n =15. One-way ANOVA was used to determine the effect of the inoculation. No significant differences were detected.

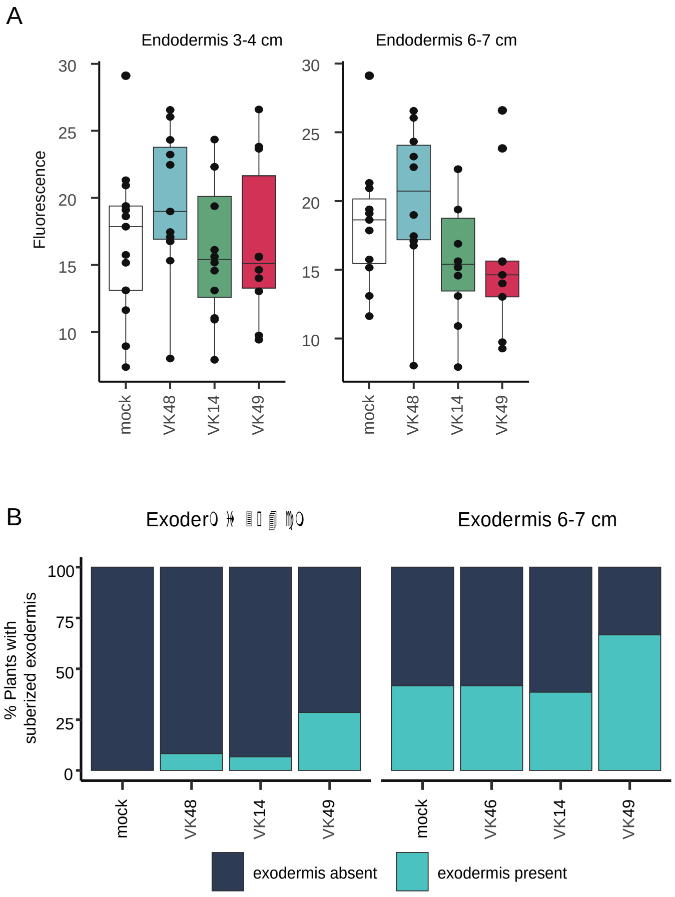

Supplementary Figure 7. Suberin content in the main root endodermis (A) of plants inoculated with Arthrobacter strains VK48, VK 2073 and VK 2105. Suberin was stained with fluorol yellow and quantified with mean intensity of pixel. One-way ANOVA was used to determine the effect of the inoculation. No significant differences were detected. B) Percentage of plants with a suberized or non-suberized exodermis in the root region 3-4 cm and 6-7 cm from the root tip, upon inoculation with Arthrobacter strains VK 1979, VK 2073 and VK 2105. No significant differences were detected as per Fisher exact test.

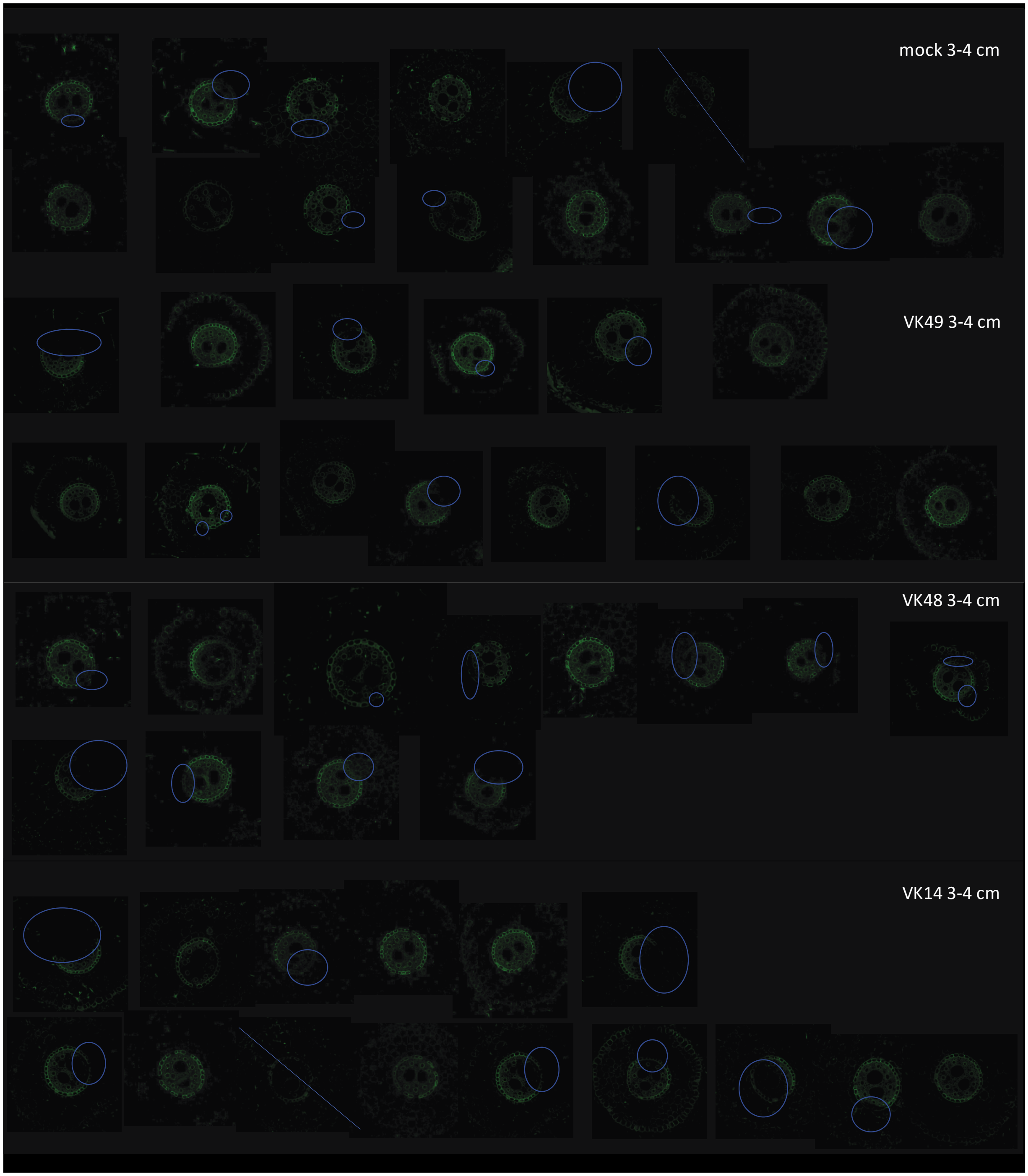

Supplementary Figure 8. Raw images used for quantification of the proportion of suberized cells in endodermis. Blue circle denotes regions that were excluded from the analysis (see Methods).
