# Supplementary material for: The soil microbiome modulates the sorghum root metabolome and cellular traits with a concomitant reduction of Striga infection
Source: Cell Rep. 2024 Mar 26;43(4):113971. doi: 10.1016/j.celrep.2024.113971 (PMC11063626; doi:10.1016/j.celrep.2024.113971)
Supplement: Data S6. An overview of bacterial taxa found at two and three weeks post-infection in microbial sub-categories tested, predicted to influence Striga infection via each of identified mechanisms [file mmc7.pdf]

A

Bacteria Bulk soil 2 wpi

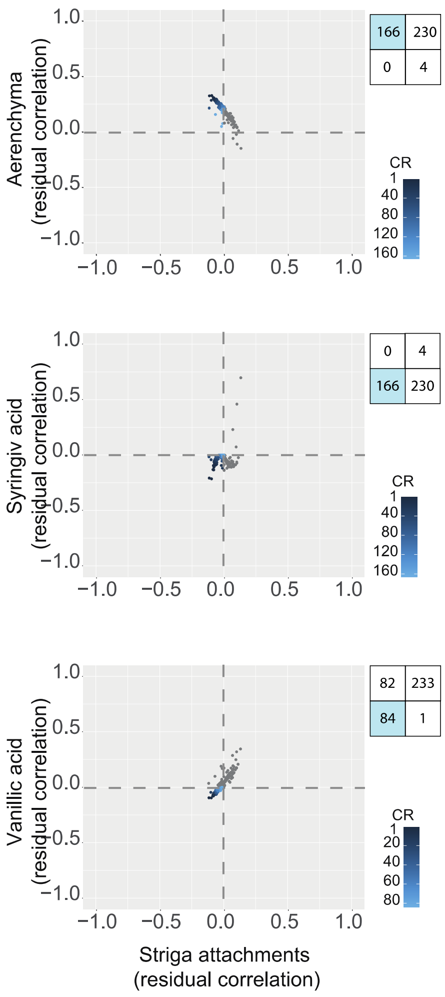

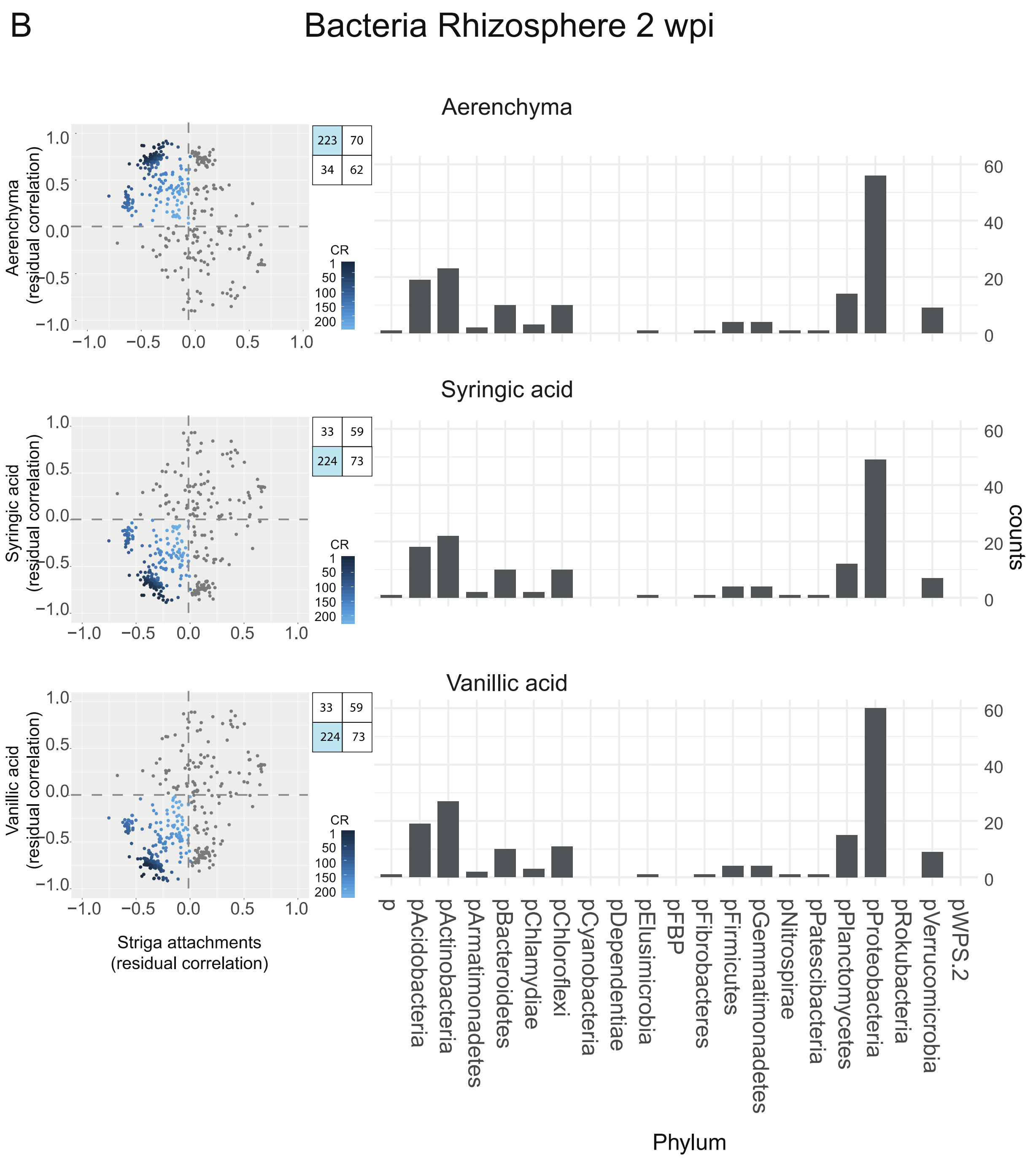
B
Bacteria Rhizosphere 2 wpi
Aerenchyma
Aerenchyma (residual correlation)
223
70
34
62
CR
1
50
100
150
200
Syringic acid
Syringic acid (residual correlation)
33
59
224
73
Vanillic acid
Vanillic acid (residual correlation)
33
59
224
73
counts
Striga attachments (residual correlation)
−1.0
−0.5
0.0
0.5
1.0
0
20
40
60
p
pAcidobacteria
pActinobacteria
pArmatimonadetes
pBacteroidetes
pChlamydiae
pChloroflexi
pCyanobacteria
pDependentiae
pElusimicrobia
pFBP
pFibrobacteres
pFirmicutes
pGemmatimonadetes
pNitrospirae
pPatescibacteria
pPlanctomycetes
pProteobacteria
pRokubacteria
pVerrucomicrobia
pWPS.2
Phylum

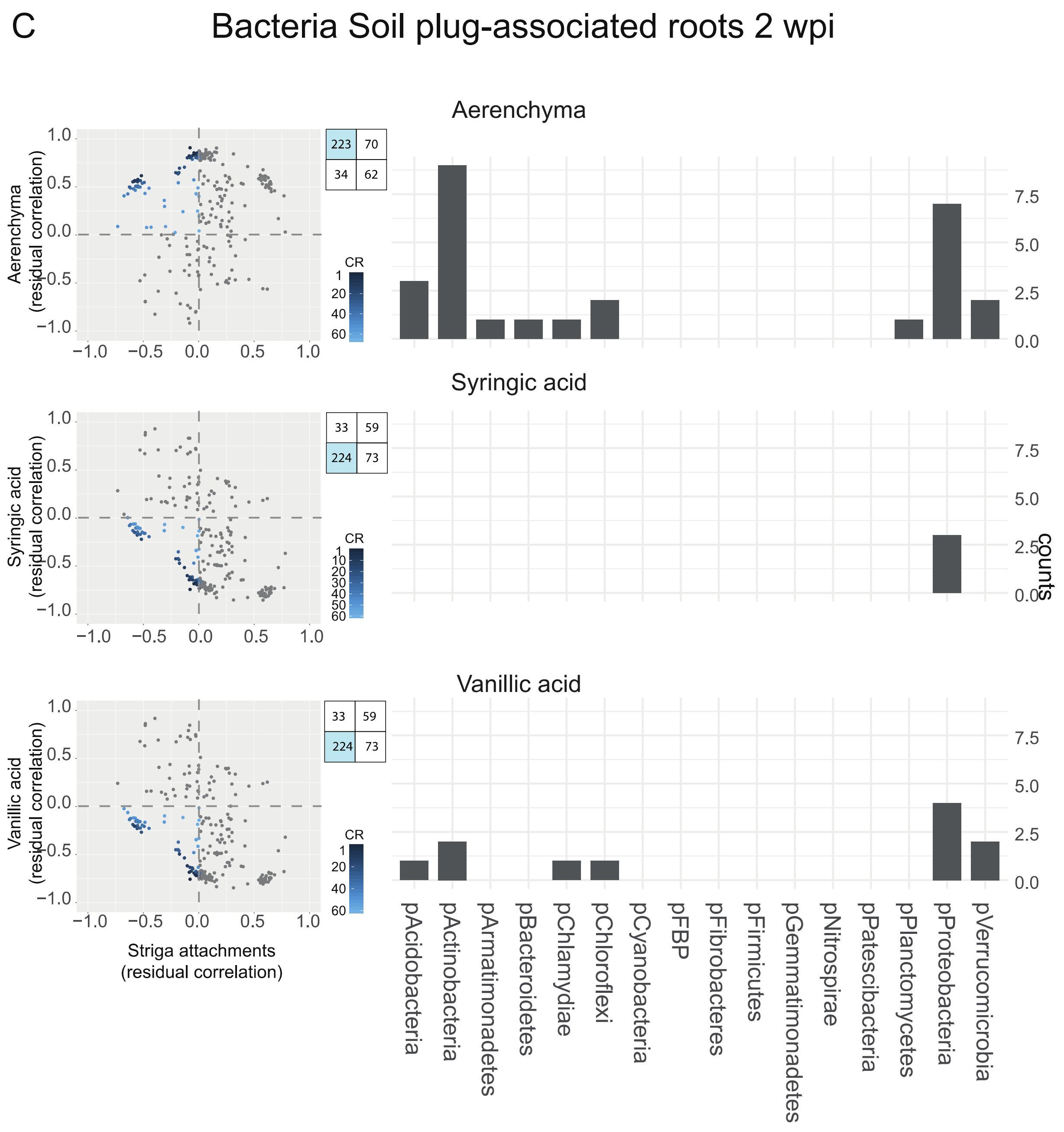
C
Bacteria Soil plug-associated roots 2 wpi
Aerenchyma
Aerenchyma (residual correlation)
223
70
34
62
CR
Syringic acid
Syringic acid (residual correlation)
33
59
224
73
Vanillic acid
Vanillic acid (residual correlation)
33
59
224
73
Striga attachments (residual correlation)
counts
Phylum
pAcidobacteria
pActinobacteria
pArmatimonadetes
pBacteroidetes
pChlamydiae
pChloroflexi
pCyanobacteria
pFBP
pFibrobacteres
pFirmicutes
pGemmatimonadetes
pNitrospirae
pPatescibacteria
pPlanctomycetes
pProteobacteria
pVerrucomicrobia

D

Bacteria Bulk soil 3 wpi

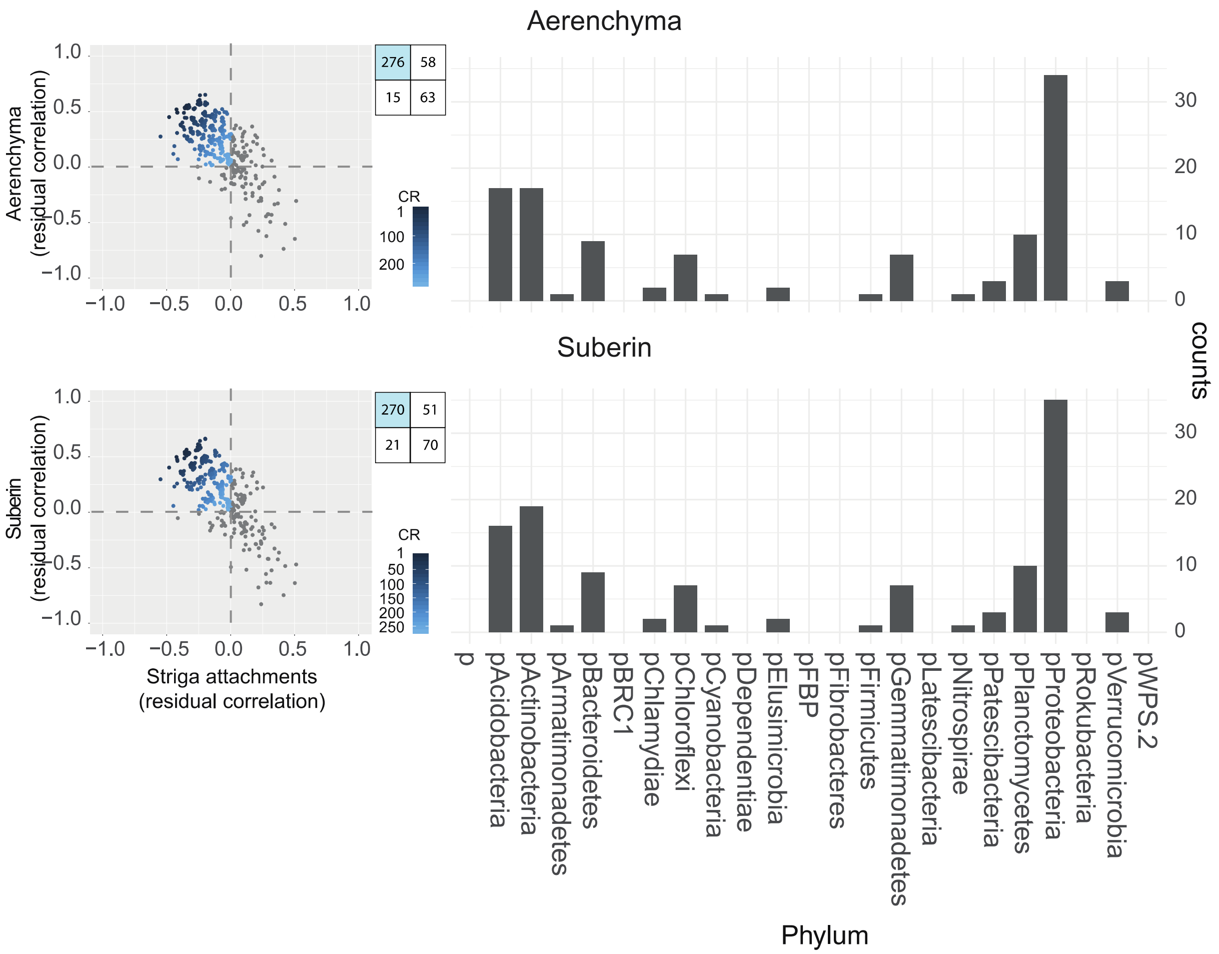

E

Bacteria Rhizosphere 3 wpi

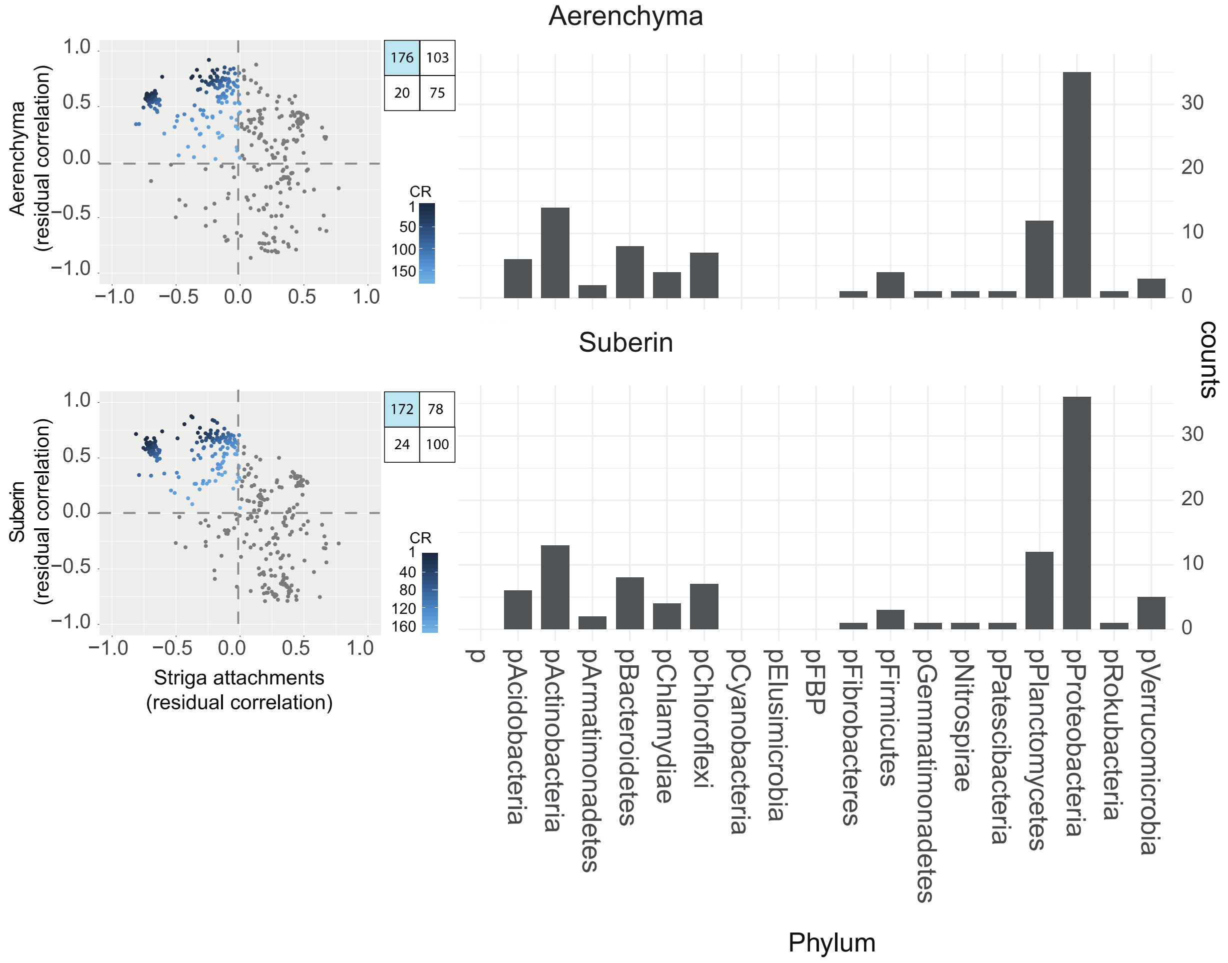

F

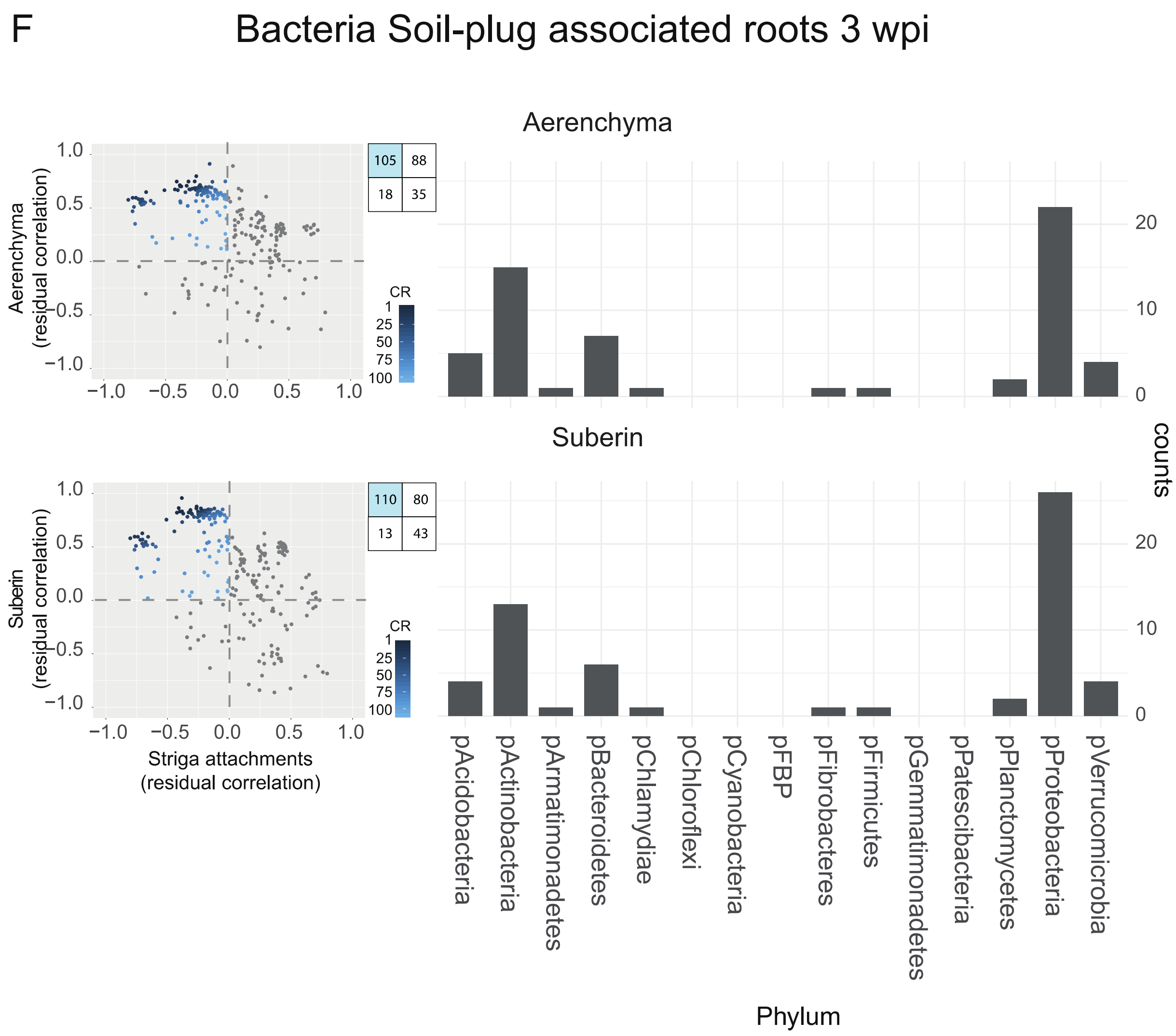

Bacteria Soil-plug associated roots 3 wpi
Aerenchyma
Aerenchyma (residual correlation)
1.0
0.5
0.0
−0.5
−1.0
−1.0
−0.5
0.0
0.5
1.0
105
88
18
35
CR
1
25
50
75
100
20
10
0
Suberin
Suberin (residual correlation)
110
80
13
43
counts
Striga attachments (residual correlation)
pAcidobacteria
pActinobacteria
pArmatimonadetes
pBacteroidetes
pChlamydiae
pChloroflexi
pCyanobacteria
pFBP
pFibrobacteres
pFirmicutes
pGemmatimonadetes
pPatescibacteria
pPlanctomycetes
pProteobacteria
pVerrucomicrobia
Phylum

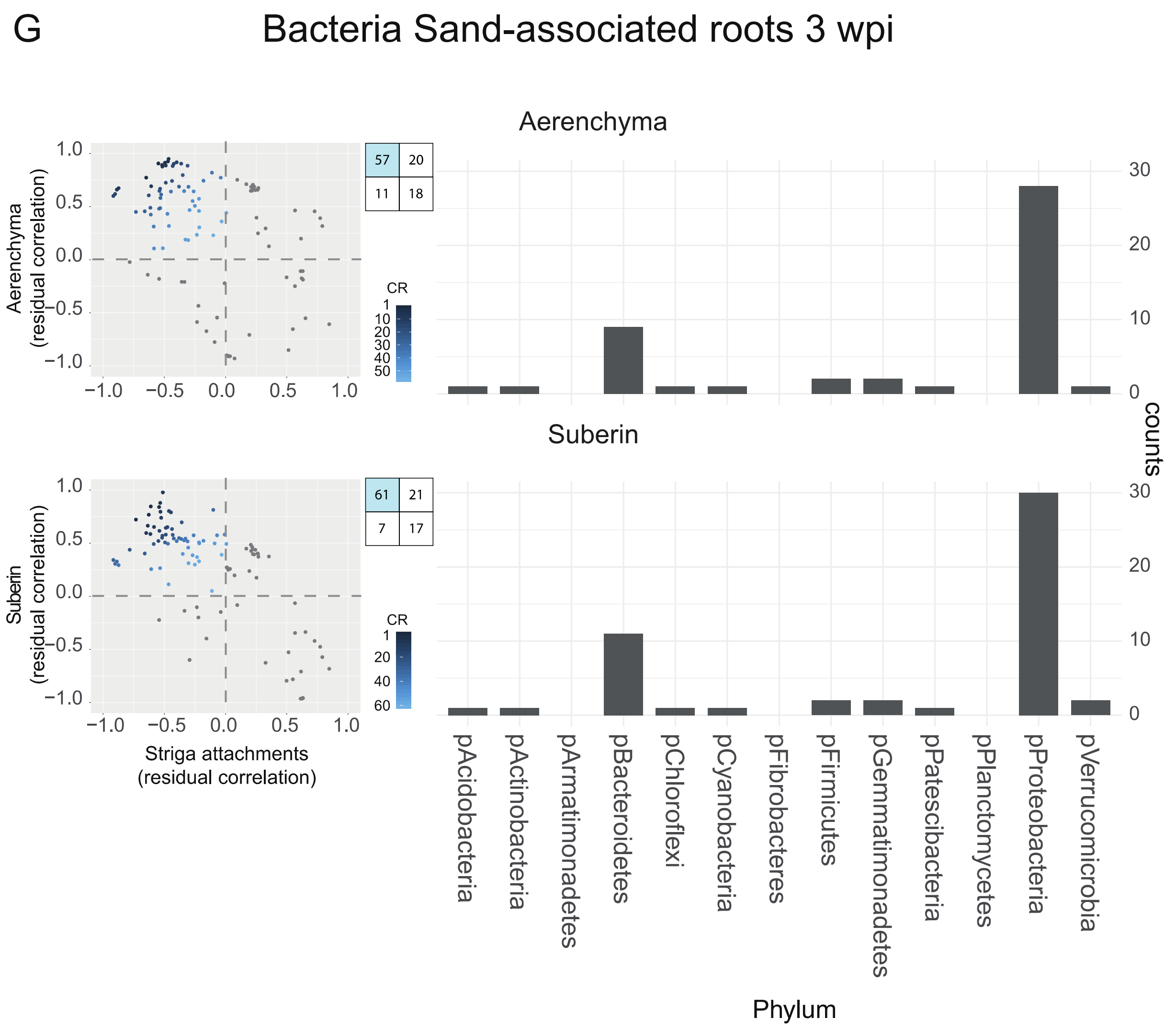

Supplementary Dataset 6. Overview of bacterial taxa found at two weeks post-infection in (A) bulk soil, (B) rhizosphere, (C) soil plug-associated roots and three weeks post-infection in (D) bulk soil, (E) rhizosphere, (F) soil plug-associated roots, (G) sand-associated roots, predicted to influence Striga infection via each of identified mechanisms (see Methods). Left panel: each dot represents individual bacterial taxon and its residual correlation found for Striga attachment (x-axis) and one of the identified mechanisms (y-axis). The four-square inset indicates number of taxa found to be: (i) negatively correlated with Striga attachment number and positively with each mechanism (left, upper square), (ii) positively correlated with Striga attachment number and positively with each mechanism (right, upper square), (iii) negatively correlated with Striga attachment number and negatively with each mechanism (left, lower square), (iv) positively correlated with Striga attachment number and negatively with each mechanism (left, upper square). The blue shading in the four-square inset indicates the number of taxa which were used for combined ranking and which represent taxa predicted to reduce Striga infection given the trait under study. Within the residual correlation plots, the intensity of blue represents their combined rank value (CR). Right panel: Number of bacteria from each phylum found to reduce Striga infection via each of the mechanisms with the cut-off of residual correlation -0.2 for Striga attachments, syringic acid and vanillic acid levels and 0.2 for aerenchyma proportion and suberin content. No bacteria passed the threshold in bulk soil 2wpi (A).
