# Supplementary material for: The soil microbiome modulates the sorghum root metabolome and cellular traits with a concomitant reduction of Striga infection
Source: Cell Rep. 2024 Mar 26;43(4):113971. doi: 10.1016/j.celrep.2024.113971 (PMC11063626; doi:10.1016/j.celrep.2024.113971)
Supplement: Document S2. Article plus supplemental information [file mmc9.pdf]

Cell Reports

Article

The soil microbiome modulates the sorghum root metabolome and cellular traits with a concomitant reduction of Striga infection

Graphical abstract

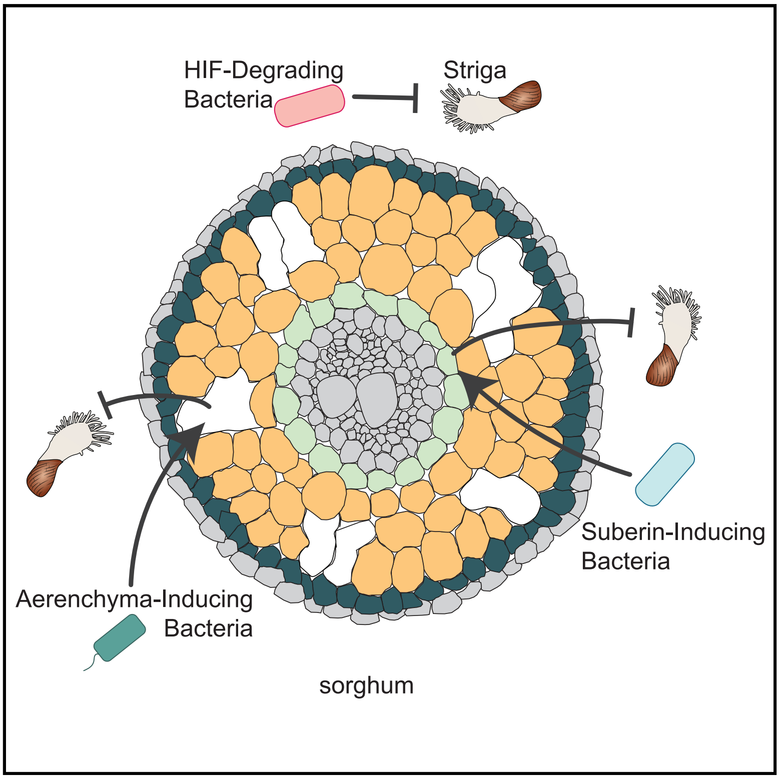

Highlights

- The soil microbiome hinders Striga parasitism of sorghum roots
- A Striga-suppressive microbiome tweaks root exudate, aerenchyma, and suberin content
- *Pseudomonas* strain VK46 reduces haustorium formation by degrading syringic acid
- *Arthrobacter* strain VK49 increases sorghum endodermal suberization

Authors

Dorota Kawa, Benjamin Thiombiano, Mahdere Z. Shimels, ..., Jos M. Raaijmakers, Harro Bouwmeester, Siobhan M. Brady

Correspondence

dkawa@uu.nl (D.K.), sbrady@ucdavis.edu (S.M.B.)

In brief

The soil microbiome protects sorghum, and likely other crops, from infection with the root parasite *Striga hermonthica*. Kawa et al. show that soil-borne bacteria modify sorghum root development and root exudate content, likely preventing Striga from penetrating sorghum. This study provides a framework for developing microbial-based solutions for Striga infestation.

Kawa et al., 2024, Cell Reports *43*, 113971
April 23, 2024 © 2024 The Authors.
https://doi.org/10.1016/j.celrep.2024.113971

CellPress

Cell Reports

CellPress
OPEN ACCESS

Article

The soil microbiome modulates the sorghum root metabolome and cellular traits with a concomitant reduction of Striga infection

Dorota Kawa,[1,2,3,*] Benjamin Thiombiano,[4] Mahdere Z. Shimels,[5] Tamera Taylor,[1,6] Aimee Walmsley,[4] Hannah E. Vahldick,[1] Dominika Rybka,[5] Marcio F.A. Leite,[5] Zayan Musa,[1] Alexander Bucksch,[7,8,9] Francisco Dini-Andreote,[5,10,11] Mario Schilder,[4] Alexander J. Chen,[1] Jiregna Daksa,[1] Desalegn W. Etalo,[5,14] Taye Tessema,[12] Eiko E. Kuramae,[5,13] Jos M. Raaijmakers,[5] Harro Bouwmeester,[4] and Siobhan M. Brady[1,15,*]

[1]Department of Plant Biology and Genome Center, University of California, Davis, Davis, CA 95616, USA
[2]Plant Stress Resilience, Department of Biology, Utrecht University, 3508 TC Utrecht, the Netherlands
[3]Environmental and Computational Plant Development, Department of Biology, Utrecht University, 3508 TC Utrecht, the Netherlands
[4]Plant Hormone Biology Group, Green Life Sciences Cluster, Swammerdam Institute for Life Science, University of Amsterdam, 1098 XH Amsterdam, the Netherlands
[5]Netherlands Institute of Ecology (NIOO-KNAW), Department of Microbial Ecology, 6708 PB Wageningen, the Netherlands
[6]Plant Biology Graduate Group, University of California, Davis, Davis, CA 95616, USA
[7]Department of Plant Biology, University of Georgia, Athens, GA 30602, USA
[8]Institute of Bioinformatics, University of Georgia, Athens, GA 30602, USA
[9]Warnell School of Forestry and Natural Resources, University of Georgia, Athens, GA 30602, USA
[10]Department of Plant Science, The Pennsylvania State University, University Park, PA 16802, USA
[11]Huck Institutes of the Life Sciences, The Pennsylvania State University, University Park, PA 16802, USA
[12]Ethiopian Institute of Agricultural Research, 3G53+6XC Holeta, Ethiopia
[13]Ecology and Biodiversity, Department of Biology, Utrecht University, 3584 CH Utrecht, the Netherlands
[14]Wageningen University and Research, Laboratory of Phytopathology, Wageningen, the Netherlands
[15]Lead contact
*Correspondence: dkawa@uu.nl (D.K.), sbrady@ucdavis.edu (S.M.B.)
https://doi.org/10.1016/j.celrep.2024.113971

SUMMARY

Sorghum bicolor is among the most important cereals globally and a staple crop for smallholder farmers in sub-Saharan Africa. Approximately 20% of sorghum yield is lost annually in Africa due to infestation with the root parasitic weed *Striga hermonthica*. Existing Striga management strategies are not singularly effective and integrated approaches are needed. Here, we demonstrate the functional potential of the soil microbiome to suppress Striga infection in sorghum. We associate this suppression with microbiome-mediated induction of root endodermal suberization and aerenchyma formation and with depletion of haustorium-inducing factors, compounds required for the initial stages of Striga infection. We further identify specific bacterial taxa that trigger the observed Striga-suppressive traits. Collectively, our study describes the importance of the soil microbiome in the early stages of root infection by Striga and pinpoints mechanisms of Striga suppression. These findings open avenues to broaden the effectiveness of integrated Striga management practices.

INTRODUCTION

Sorghum bicolor is one of the most important cereal crops in the world as a source of food, feed, fiber, and fuel. Its ability to withstand drought and soil aridity makes it a preferred crop in sub-Saharan Africa and earned it the name "the camel of crops."[1] Despite its outstanding resilience to abiotic stresses, approximately 20% of sorghum yield is lost annually due to infestation with the root parasitic weed *Striga hermonthica*.[2] *Striga hermonthica* infects not only sorghum but also many other crop species including rice, pearl millet, and maize. An individual Striga plant can produce thousands of tiny, easy-to-spread seeds, and its seedbank can remain dormant in soil for up to 20 years.[3] Striga is thus widespread in sub-Saharan Africa, and its occurrence has been reported in at least 32 African countries.[4,5] It is estimated that its annual cereal production losses amount to more than 6 million tons of grain annually.[5] These yield losses often lead to field abandonment and food insecurity, which particularly affects smallholder farmers in sub-Saharan Africa.

The Striga life cycle is tightly connected to its host root chemistry. Upon phosphorus deprivation, host roots exude strigolactones, carotenoid-derived compounds that serve as a signal to recruit arbuscular mycorrhizal fungi. Striga has hijacked this strigolactone signal and germinates only upon its perception.[6–8] Germinated Striga perceives other exudate compounds that act as haustorium-inducing factors (HIFs).[9] Haustorium development allows Striga to penetrate the host root tissue to reach its vasculature.[10] Further establishment of a Striga xylem-host

© 2024 The Authors.
This is an open access article under the CC BY license (http://creativecommons.org/licenses/by/4.0/).

xylem connection is known as the "essence of the parasitism."[11] Through this xylem-xylem connection, Striga deprives its host plant of nutrients, water, and macromolecules, leading to adverse effects on plant growth and yield.[12]

Currently, major practices of Striga management involve chemical control, "push-pull" methods, crop rotation, and breeding for Striga-resistant host plant varieties. Low germination stimulant (LGS1) genotypes exuding strigolactone variants with reduced capacity to induce Striga seed germination have been used to develop varieties with pre-attachment resistance.[13] Post-attachment resistance, where formation of physical barriers in root tissue prevents Striga from reaching the host vasculature, has been found in few sorghum landraces.[14] Despite these efforts, each management strategy has only partial Striga mitigation efficiency.[15] Moreover, these measures are often not available to smallholder farmers in sub-Saharan countries, where the most common solution is manual weed removal. Thus, there is a need for new and effective methods that can be integrated into current agricultural practices. Microbial-based solutions leveraging the soil suppressiveness phenomenon can meet these criteria.

Suppressiveness of soils to root diseases has been studied for bacterial, fungal, and oomycete pathogens. In most cases, the suppressiveness is microbial in nature, as it can be eliminated by sterilization or pasteurization of the soil and can be transplanted to non-suppressive soils.[16] In the disease-suppressive soils, despite the presence of a virulent pathogen, disease symptoms are less severe or do not occur at all, or the pathogen is able to initially cause a disease that later declines in severity.[17] Thus far, the mechanisms of disease suppressiveness are best understood in the case of fungal root pathogens.[17,18] Little fundamental knowledge is available on the functional potential of the soil microbiome to interfere in the infection cycle of Striga and other plant parasitic weeds.

Masteling et al. proposed several potential mechanisms by which microbes can suppress parasitic plant infection.[19] Microbes can interfere directly with the parasite's life cycle either through their pathogenic effect on parasite seeds or by reduction of parasite seed germination and haustorium formation. The latter can occur via disruption of the biosynthesis or degradation of strigolactones and HIFs.[19] Microbes could also act indirectly by affecting either the host plant itself or its environment. Microbes could enhance host nutrient acquisition and as a result reduce strigolactone exudation and, subsequently, parasite seed germination. Alternatively, microbes could induce changes in root system or cellular architecture, providing an avoidance mechanism or creating physical barriers, respectively. Lastly, microbes could also induce local or systemic resistance in the host plant.[19]

To date, several mechanisms by which microbes directly influence the Striga life cycle have been described including suppression of Striga seed germination by strains of *Pseudomonas*[20] and infection of Striga by *Fusarium oxysporum* f.sp. *strigae*.[21] Following these studies, a *Fusarium*-based inoculant has been developed and integrated into agricultural practices in Kenya, resulting in an increase in maize yield in Striga-infested fields.[21] Thus far, indirect effects of the soil microbiome on Striga infection of sorghum have not been investigated and mechanistically resolved.

Here, we provide a proof of concept for the potential suppressive effects soil-borne microbes can have on the early stages of Striga infection. We identify a soil whose microbiome reduces Striga infection in sorghum and use it as a discovery tool to identify the modes of action of microbiome-based Striga suppression, with a focus on host root chemistry and root cellular traits. We show that in the presence of the microbiome, sorghum HIFs are degraded in this soil with a concomitant adverse effect on haustorium formation. Moreover, in the presence of the soil microbiome, we observed changes in root cellular anatomy including cortical aerenchyma formation and endodermal suberin deposition. We further identify specific soil bacterial taxa associated with Striga suppression and which operate by inducing changes in root cellular traits or by degrading specific HIFs. Our data reveal that specific soil bacteria can induce changes in host roots associated with protection against Striga. Our findings provide a foundation to harness the protective effects of microbes in integrated Striga management practices.

RESULTS

The soil microbiome impedes the post-germination stages of Striga infection

To explore the existence of soil suppressiveness to Striga, we selected a soil from the Netherlands referred to as the "Clue Field" soil.[22] Despite its origin from an area where sorghum is not cultivated, the Clue Field soil has been previously used for soil and sorghum rhizosphere microbiome studies, as well as for sorghum root phenotyping.[22,23] We gamma irradiated a batch of this soil for the purpose of sterilization and ensured that gamma sterilization did not affect the physico-chemical properties of the soil (Data S1). We profiled the soil microbiome composition by sequencing 16S rRNA gene (bacteria) and internal transcribed spacer region (fungi) amplicons from the DNA extracted from bulk non-irradiated and gamma-irradiated soil. The alpha diversity of the bacterial composition of the non-irradiated soil was higher than that of the gamma-irradiated soil (Figure 1A), while fungal composition was comparable between the two soils (Figure 1B). The gamma-irradiated soil will herein be referred to as "sterilized" soil and the non-irradiated soil as "natural" soil.

To test the effect of the soil microbiome on Striga infection in sorghum, we grew seedlings of Striga-susceptible Shanqui Red (SQR) for 10 days in 50 mL of either natural or sterilized soil to allow for microbial colonization of their roots (Figure S1). The 10-day-old seedlings, along with the soil "plug," were transferred to larger pots with sand (control) or sand mixed with preconditioned *Striga hermonthica* seeds. The number of Striga attachments to sorghum roots were counted at 2 and 3 weeks post-infection, which corresponds to 4- and 5-week-old plants, respectively. We observed significantly fewer Striga attachments on SQR roots grown in the natural soil as opposed to the sterilized soil 2 and 3 weeks post-infection (Figure 1C; Data S1). This observation suggests that the natural soil contains microbial taxa that suppress Striga infection.

Next, we asked at which stage of the Striga life cycle this suppression occurs. We set out to determine whether the functional outcome of the chemical signals governing Striga seed germination (host-derived strigolactones) and haustorium formation

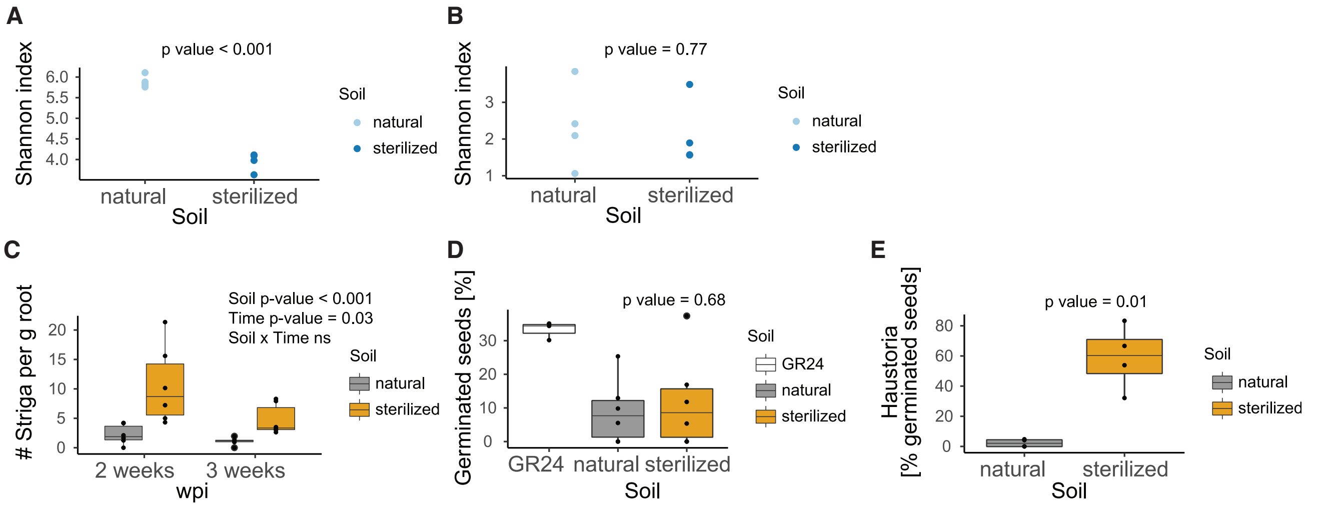

Figure 1. The soil microbiome suppresses Striga infection in sorghum

(A and B) Alpha diversity of (A) bacterial and (B) fungal communities of the field-collected soil ("natural") and its gamma-irradiated counterpart ("sterilized"). Significance of the differences was determined with a Welch t test (n = 4).

(C) Number of Striga attachments per gram of fresh root weight of the Striga-susceptible variety Shanqui Red (SQR) at 2 and 3 weeks post-infection (wpi) in natural and sterilized soil. The significance of the differences was assessed with a two-way ANOVA (n = 6).

(D and E) *In vitro* (D) germination and (E) haustorium formation of Striga seeds exposed to root exudates collected from 4-week-old sorghum plants grown in natural and sterilized soil. The synthetic strigolactone, GR24, was used as a positive control for germination assay. Significance of the differences was assessed with a Welch t test (exudates from six plants per soil were used with three technical replicates per exudate). Boxplots in (A)–(E) denote the span from the 25th to 75th percentile and are centered to the data median.

(host-derived HIFs)[7,24] is dependent on the soil microbial complement. To this end, we collected root exudates from 4-week-old SQR plants grown in the natural and sterilized soils in the absence of Striga and applied them to Striga seeds in an *in vitro* assay. We observed no difference in the germination percentage between seeds treated with sorghum root exudates from natural and sterilized soil (Figure 1D). However, we noted a difference in the percentage of Striga seeds that formed early stages of haustoria. More than 60% of the Striga seeds exposed to the exudates from the sterilized soil developed haustoria, whereas few haustoria were formed in the presence of the exudates of plants grown in the natural soil (Figure 1E). Together, these results suggest that members of the natural soil microbiome reduce Striga infection of the susceptible sorghum cultivar at the post-germination stage of the parasite's life cycle by interfering with haustorium initiation by host-derived cues, the HIFs.

The microbiome reduces the levels of haustorium-inducing factors in the sorghum rhizosphere

The low level of haustorium initiation by the root exudates from plants grown in the natural soil suggests that the microbial component of this soil affects the abundance of the HIFs. To test this hypothesis, we measured the levels of known HIFs in the exudates of SQR plants grown in the natural and sterilized soil both in the absence and presence of Striga. Using a two-way ANOVA, the differential abundance of detected HIFs and their dependence on the soil microbiome, as well as Striga infection and their interaction, was determined. We detected five previously characterized HIFs[24] with differential abundance in our treatments—acetosyringone, DMBQ (2,6-dimethoxybenzoquinone), syringic acid, vanillic acid, and vanillin. Of these differentially abundant HIFs, syringic acid and vanillic acid levels were lower in exudates collected from natural soil compared to sterilized soil both in the absence and presence of Striga at 2 weeks post-infection (Figures 2A and 2B). Lower levels of DMBQ and acetosyringone were detected in the exudates of plants grown in natural soil but only in the absence of Striga (Figures 2C–2E).

Given the reduction of haustorium formation of Striga seeds exposed to exudates from sorghum plants grown in natural soil (Figure 1E) and the reduced levels of several HIFs in the exudates of plants 2 weeks post-infection (Figures 2A–2D), we hypothesized that microbes present in the natural soil degrade HIFs. To test this hypothesis, we used the BioTransformer database[25] to predict the products of potential microbial conversion of these HIFs (DMBQ, syringic acid, vanillic acid, vanillin, acetosyringone). In total, 74 compounds were predicted as potential HIF break-down products (Data S1). In the untargeted metabolite profiles of root exudates from 4-week-old plants grown in natural or sterilized soil, we identified 82 features predicted to be HIF conversion products. Among these 82 features, the abundance of 26 compounds differed significantly between exudates of sorghum plants grown in the natural or sterilized soils (adjusted p value < 0.05, $\log_2$ fold change > 1 or < -1). The majority (73%) of these compounds accumulated to higher levels in exudates from plants grown in natural than in sterilized soil (Figure 2F). This indicates that in the presence of the soil microbiome from the natural soil, the putative HIF conversion products were more prevalent than the HIFs themselves. Collectively, these results suggest that degradation of HIFs by members of the

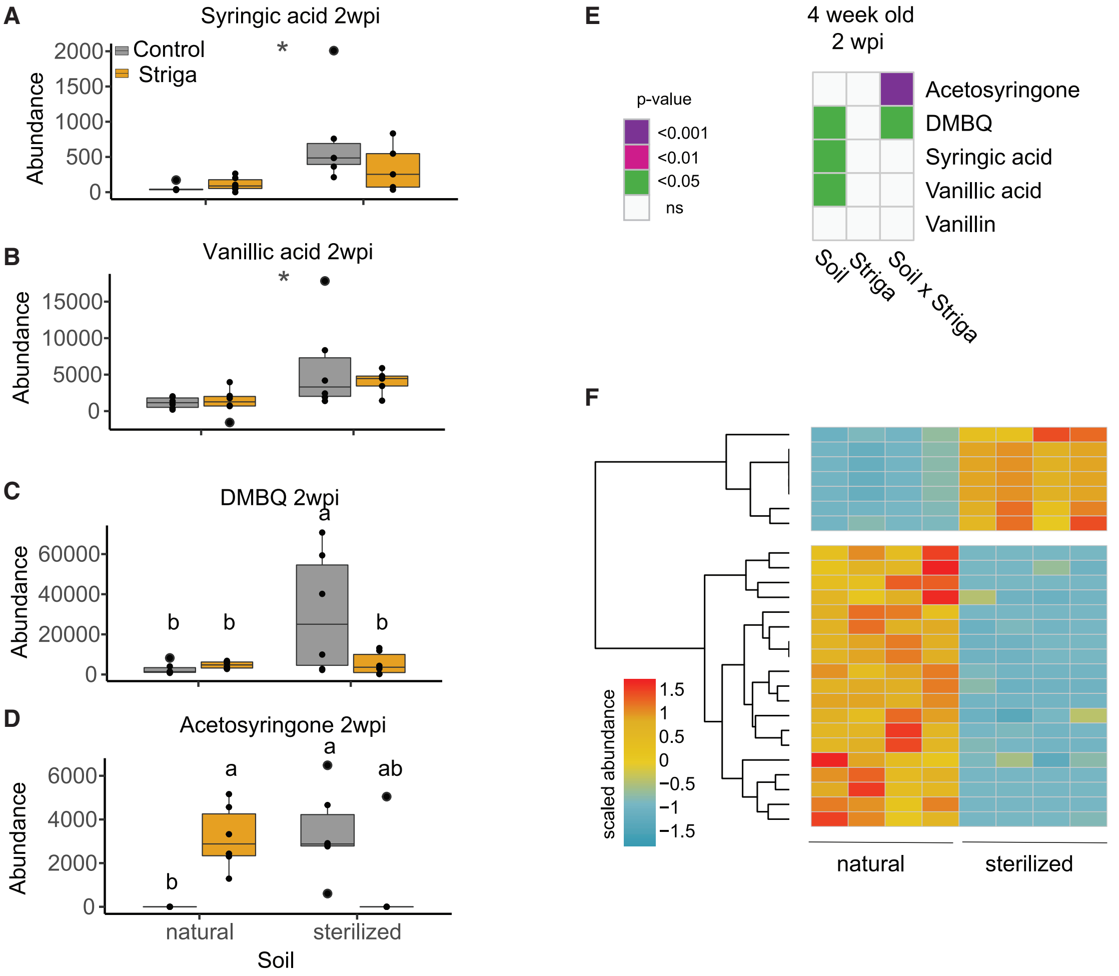

Figure 2. The soil microbiome influences haustorium-inducing factor (HIF) abundance in root exudates

(A–D) Relative abundance (measured as a peak area) of (A) syringic acid, (B) vanillic acid, (C) DMBQ, and (D) acetosyringone at 2 weeks post-infection (wpi). Asterisks denote the significance of the soil impact, while different letters show the significance of the differences between groups for traits, where a soil-by-Striga interaction effect was detected (Tukey post hoc test). Boxplots denote the span from the 25th to 75th percentile and are centered to the data median.

(E) Heatmap presenting the impact of the soil microbiome, Striga infection, and their interaction on the abundance of HIFs in root exudates as determined using a two-way ANOVA. Data are presented are from 2 wpi with Striga, which corresponds to 4-week-old sorghum plants. Purple, pink, and green colors denote the significant impact of the soil, Striga, and their interaction. White squares indicate the lack of a significant effect (n = 6).

(F) Abundance of features identified with untargeted metabolite profiling corresponding to potential HIF conversion products in root exudates collected from 4-week-old plants grown in the natural and sterilized Clue Field soil (n = 4). Values presented are the area under the associated peak scaled to the mean across all samples. The heatmap presents values for 26 compounds whose abundances differed significantly between exudates of plants grown in the two soils (adjusted p value < 0.05, $\log_2$ fold change > 1 or < −1).

microbiome is likely associated with the reduction of Striga infection of sorghum plants grown in the natural soil.

The soil microbiome modifies root cellular anatomy and corresponding transcriptional programs

Despite the ability of the soil microbiome to inhibit haustorium initiation, we still observed several Striga attachments on the roots of plants grown in natural soil (Figure 1C). Thus, we next assessed whether the microbiome complement of this soil elicits additional changes in host root morphology that could influence Striga attachment and penetration. We conducted a detailed characterization of root system architecture (RSA) and cellular anatomy to determine if any root traits are influenced by the soil microbiome, Striga infection, or their interaction. To separate the influence of the microbiome from Striga, we used a two-way ANOVA as previously conducted for the HIF analysis. The RSA data of SQR plants grown in sterilized soil in the absence of Striga was previously published in Kawa et al.[23] Similar to HIF abundance in the root exudates, the soil microbiome affected the root traits in a manner independent of Striga infection (linear model term: soil) as well as in a more complex manner dependent on Striga infection (linear model term: soil x Striga) (Figure 3A). As with HIF abundance, for subsequent experiments, we considered only those traits that the microbiome changed independently of Striga.

The RSA of the mature sorghum plant consists of seminal and crown roots; thus, each trait was quantified separately

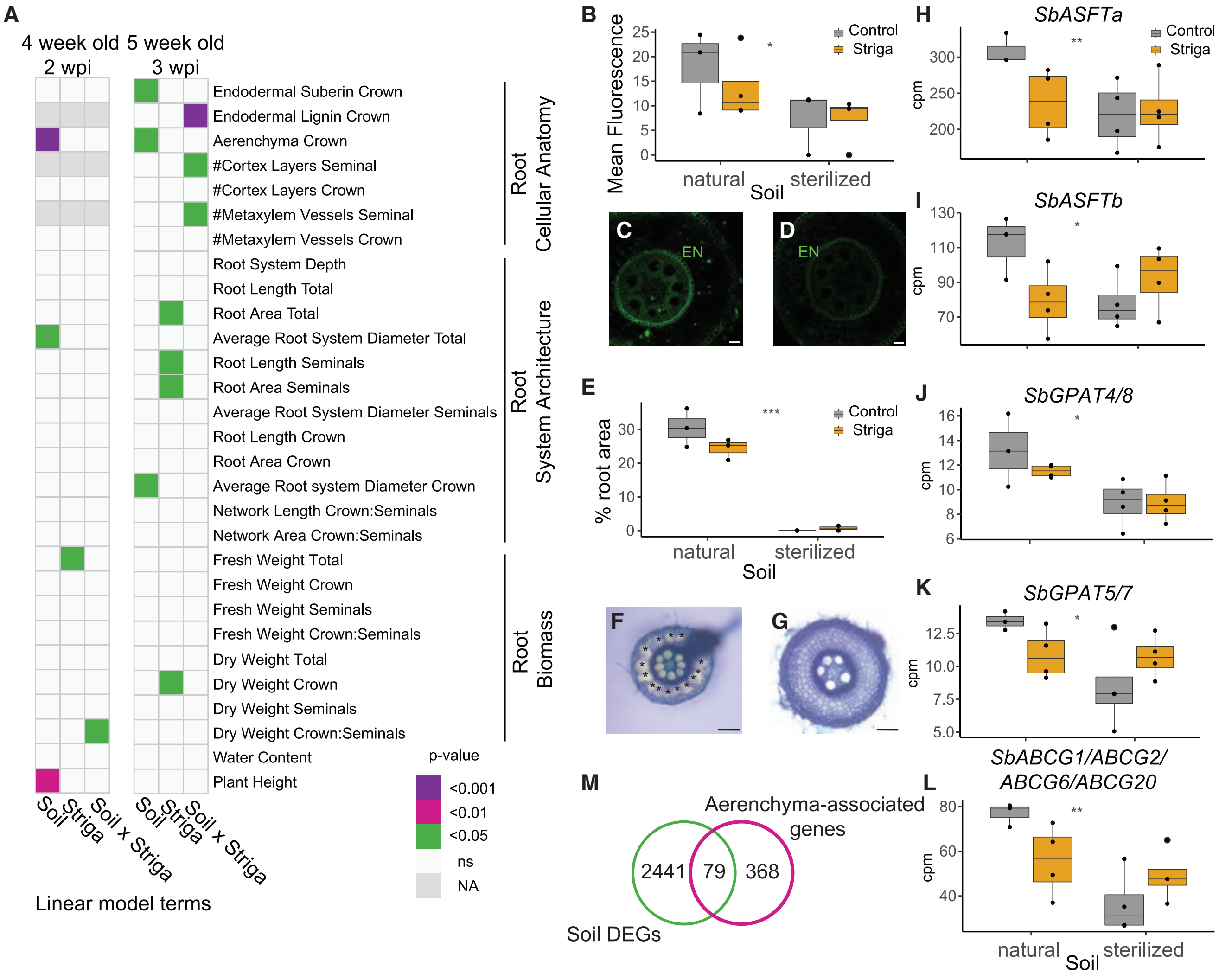

Figure 3. The soil microbiome induces changes in root system architecture and cellular anatomy

(A) Heatmap presenting the impact of the soil microbiome (Soil), Striga infection (Striga), and their interaction (Soil x Striga) on root cellular anatomy, root system architecture, and root biomass as determined by a two-way ANOVA. Data presented are from sorghum at 2 and 3 weeks post-infection (wpi) with Striga, which correspond to 4- and 5-week-old sorghum plants, respectively. Purple, pink, and green colors denote the significance threshold associated with the impact of the soil, Striga, and their interaction on a given trait. White squares indicate a lack of significant effect, while NA denotes that the trait was not tested at a given time point. The number of biological replicates tested for each trait is listed in Data S1.

(B–G) The suberin content (as measured by mean fluorol yellow pixel intensity) in the endodermis of sorghum crown roots 3 wpi with Striga (B–D) and aerenchyma proportion in the cortex of sorghum crown roots 3 wpi with Striga (E–G) grown on natural (C and F) and sterilized (D and G) soil. Aerenchyma area is expressed as a proportion of the whole root cross-section area. Asterisks in (F) indicate aerenchyma. Root cross-sections in (F) and (G) were stained with toluidine blue. Scale bar: 50 μm. Cross-sections in (F)–(G) were prepared from the exact same region of the exact same root as for suberin visualization.

(H–L) Expression of suberin biosynthetic genes (H) *SbASFTa*, (I) *SbASFTb*, (J) *SbGPAT4/8*, (K) *SbGPAT5/7*, and (L) *SbABCG1/ABCG2/ABCG6/ABCG20.* Expression of these genes was found to be regulated by the Clue Field soil microbiome (*adjusted p value < 0.05, **adjusted p value < 0.01).

Gray asterisks indicate the (B and E) p value or the (H–K) adjusted p value for the term Soil by a two-way ANOVA: *p < 0.05, **p < 0.01, and ***p < 0.001.

(M) Enrichment of sorghum orthologs of maize genes associated with root aerenchyma formation among the genes found to be regulated by the Clue Field soil microbiome (p = 0.008; Fisher's exact test).

Boxplots (B and E and H–L) denote the span from the 25th to 75th percentile and are centered to the data median. Dots represent individual values.

for crown and seminal roots, as well as for the entire root system (Figure S1). The soil microbiome had only a marginal effect on RSA and affected only the average diameter of the whole root system (at 2 weeks post-infection) or of the crown roots (at 3 weeks post-infection) (Figure 3A). Although the goal of these experiments was to decipher the influence of the soil microbiome on Striga infection from the perspective of the host, we also observed a significant impact of Striga on RSA. The effect of Striga on RSA was more pronounced at 3 weeks post-infection. Here, the total length and area of the root system and seminal root length were greater in plants infected with Striga when compared to non-infected plants

regardless of the soil type (Figures 3A, S2A, S2E, and S2F). We also observed a higher dry biomass of crown roots in plants 3 weeks post-infection as compared to its non-infected control (Figures 3A, S2A, and S2H).

The soil microbiome primarily influenced root cellular anatomy traits (Figure 3A). More endodermal suberization was observed in crown roots of 5-week-old plants in natural soil independent of Striga infection status (Figures 3B–3D). Additionally, more aerenchyma formed in crown roots of 4- and 5-week-old plants grown in natural soil, as compared to the sterilized soil, independent of Striga infection status (Figures 3E–3G). Similar to acetosyringone and vanillin levels, several root anatomy traits varied in a complex way dependent on the interaction of the soil microbiome and Striga infection, including the number of cortex layers and metaxylem vessels in seminal roots and lignification of the endodermis in crown roots (Figures 3A and S2A–S2D).

Our data suggest that the microbial component of the natural soil promotes endodermal suberin deposition and aerenchyma formation concomitant with suppression of Striga infection. To determine the potential molecular mechanisms that underly these changes, we conducted transcriptome profiling of the sorghum root systems at both 2 and 3 weeks post-infection and in the presence and absence of Striga. Indeed, the transcripts of several sorghum orthologs of suberin biosynthetic genes or putative transporters—*Sobic.003G368100* (*SbASFTa*) and *Sobic.005G122800* (*SbASFTb*),[26,27] *Sobic.004G010300* (*SbGPAT4/8*),[28] *Sobic.009G16200* (*SbGPAT5/7*),[29] and *Sobic.001G413700* (*SbABCG1/SbABCG2/ABCG6/ABCG20a*) (Figure S3)[30]—were upregulated in natural soil compared to sterilized soil in the presence and absence of Striga (Figures 3H–3L). Sorghum orthologs of maize genes previously reported as associated with aerenchyma formation were also enriched among the genes upregulated in the natural versus sterilized soil ($p = 0.008$ per Fisher's exact test, Figure 3M). These transcriptome data align with the observed microbiome-mediated changes of host root cellular anatomy, which in turn is correlated with reduced Striga infection.

Identification of soil-borne microbial taxa associated with Striga suppression

To identify microbial taxa associated with reduced Striga infection observed in the natural soil, we amplicon sequenced the bacterial communities from the following sub-categories: (1) the bulk soil, (2) sorghum rhizosphere (soil directly surrounding the root system), (3) roots growing in the soil plug (referred to as "soil-plug-associated roots"), and (4) roots growing into the sand (referred to as "sand-associated roots"), all from sorghum plants grown with or without Striga (Figure S1). We used covariance in bacterial taxa abundance across conditions (natural and sterilized soil in combination with the presence and absence of Striga across two time points of infection) to determine their potential link with Striga infection suppression via the three identified mechanisms in a generalized joint attribute modeling (GJAM) approach. The outputs of these models were mined to identify taxa whose relative abundance was negatively correlated with the number of Striga attachments and either negatively correlated with the abundance of HIFs with reduced levels in the natural soil compared to the sterilized soil (vanillic acid, syringic acid) or positively correlated with suberin levels in the endodermis and aerenchyma proportion (Data S3).

Considering the negligible effect of gamma irradiation on the soil fungal community composition (Figure 1B), we first asked in which sub-category the bacterial taxa predicted to induce each of these host-root-related traits reside. We thus identified the most associated bacterial taxa (by the magnitude of residual correlation) for a given trait present in at least one sub-category (see STAR Methods). The majority of the top 100 bacterial taxa predicted to reduce Striga infection were found in the rhizosphere 3 weeks post-infection (Figure S4A). The top 100 bacterial taxa associated with a reduction in HIF levels were found in both the rhizosphere and the soil-plug-associated roots, while those predicted to induce aerenchyma formation and suberization resided in the soil-plug-associated roots (Figures S4B–S4E). Across all the sub-categories, no unique bacterial taxa were linked to each of the studied traits. For each of the five traits (Striga attachment, aerenchyma, suberin, syringic acid, and vanillic acid) across all the sub-categories, the top-ranking bacterial taxa belonged to the Gammaproteobacteria, Deltaproteobacteria, Alphaproteobacteria, Bacteroidia, and Actinobacteria (Figure S4).

Given the diverse activities of bacteria, we next set out to identify specific bacterial taxa that were linked to changes in root cellular anatomy or HIF degradation as well as to the reduction of Striga infection. We thus created a combined rank (see STAR Methods) that summarizes the potential of a given taxa to reduce Striga infection via one of the identified mechanisms (Data S5). For each soil-/root-associated sub-category (described above), we identified taxa positively associated with root cellular anatomy trait (suberin, aerenchyma) and for which the same taxa were negatively associated with Striga infection. In the case of HIFs, the taxa would be negatively associated with syringic or vanillic acid levels, and the same taxa would be negatively associated with Striga infection. The majority of putative Striga-suppressive bacteria, regardless of the trait to which they were associated, belonged to the Actinobacteria and Proteobacteria (Figure S5).

Most bacterial taxa whose abundance positively correlated with suberin or aerenchyma content negatively correlated with the number of Striga attachments; in other words, more bacteria-induced aerenchyma/suberin coincided with less Striga attachments (Data S6). Conversely, the majority of bacterial taxa negatively correlated with suberin and aerenchyma were positively correlated with Striga infection. The majority of bacterial taxa associated with an increase in aerenchyma formation at 3 weeks post-infection were also associated with suberin induction (Figure S5). Bacterial taxa of interest for further studies with the purpose of reducing Striga infection may be those that are associated with multiple mechanisms (Figure S5).

Specific bacterial isolates prevent haustoria formation and induce suberization

A collection of bacterial strains from 35 genera has previously been established from the Clue Field soil.[31] From this collection, we prioritized bacterial isolates that matched, based on their taxonomic delineation and 16S amplicon sequence similarities, with

the candidates (based on the combined ranking) identified by GJAM analysis. More specifically, we selected four *Pseudomonas* (VK46, VK51, VK6, VK50) and four *Arthrobacter* isolates (VK48, VK14, VK49, VK15) originating from the Clue Field soil and taxonomically matching the operational taxonic units identified in the GJAM analysis to determine if these isolates were able to induce changes in the root-related traits associated with Striga suppression (HIF abundance, suberization, and aerenchyma content). We first tested three *Pseudomonas* strains that were associated with HIF degradation for their ability to affect haustoria formation (Data S7). Only 2% of germinated Striga seeds developed haustoria when treated with a cell-free filtrate of syringic acid exposed for 24 h to the *Pseudomonas* isolate VK46, as opposed to 70% haustorium induction elicited in the mock control (syringic acid exposed to the media with no isolate) (Figure 4A). However, *Pseudomonas* isolate VK46 did not reduce haustoria formation in the presence of vanillic acid (Figure 4B). The two remaining *Pseudomonas* isolates tested, VK6 and VK50, did not reduce haustorium induction in the presence of either vanillic acid or syringic acid (Figures 4A and 4B). To test whether the reduced haustorium formation is a result of HIF degradation induced by VK46, we measured the levels of syringic and vanillic acid after 24 h incubation with VK46 (t_0) and 48 h after application of culture filtrate on Striga seed (t_{48}). The same levels of syringic and vanillic acid were detected in mock (sterile) filtrates at t_0 and t_{48} after application to Striga seeds, which corresponded to the same levels of haustoria induction (Figures 4C–4F). In the presence of VK46, both syringic and vanillic acid were not detected (Figures 4E and 4F). However, degradation of only syringic acid was accompanied by reduced haustorium formation (Figure 4C). Degradation of vanillic acid resulted in a similar level of haustoria formed to that observed in the mock (Figure 4D). *Pseudomonas*, together with *Arthrobacter*, was also predicted by GJAM to reduce Striga infection via induction of aerenchyma (Data S7). None of the four *Pseudomonas* and none of the four *Arthrobacter* isolates we tested reproducibly induced aerenchyma formation (Figure S6).

Arthrobacter was the only genus associated (based on GJAM) with an increase in suberin content and Striga resistance for which isolates were available in the Clue Field bacterial collection (Data S7). In plant roots, suberin deposition occurs in three stages. In the first stage, there is an absence of suberin within the root meristem, followed by a "patchy" zone and a fully suberized zone in differentiated root.[28,32] Typically, suberin in roots is quantified by measuring its levels in a few representative cells from the cross-section (quantitative suberization) or by measuring the proportion of the non-suberized, patchy, and fully suberized zones within the root length (developmental suberization).[28,32] The latter is challenging for sorghum due to the high level of autofluorescence signal from its roots interfering with the suberin signal from fluorol yellow stain. We thus quantified the proportion of suberized cells within the endodermis in a radial cross-section and the proportion of plants with a fully suberized endodermis in a radial cross-section within the transition region between fully and patchy suberized zones (3–4 cm from the primary root tip; see STAR Methods). We additionally quantified the effect of microbial inoculation on suberization of the exodermis by quantifying the number of plants that developed a suberized exodermis.

Out of three tested *Arthrobacter* isolates (VK48, VK14, VK49), none induced quantitative differences in the fully differentiated endodermis (6–7 cm from the root tip) or in the transition region between fully suberized and patchy zones (3–4 cm from the root tip; Figure S7A). However, in plants inoculated with the *Arthrobacter* isolate VK49, significantly more suberized cells were found within 3–4 cm from the root tip than in the region that constituted a patchy suberization zone in non-inoculated plants (Figure 4G). Moreover, more plants developed a fully suberized endodermis when inoculated with *Arthrobacter* VK49 isolate than in non-inoculated plants (Figure 4H). Nearly 80% of the sorghum plants inoculated with *Arthrobacter* isolate VK49 developed a fully suberized endodermis, as opposed to 20% observed for the mock treatment (Figure 4H). This increase in suberization extended to the root exodermis with a slightly precocious deposition of suberin in the exodermis (Figure S7B). Collectively, these results provide proof of concept that microbiome analyses can lead to the isolation of individual bacterial strains that can perturb both the timing of suberin deposition as well as the number of suberized cells within the endodermal or exodermal cell files.

DISCUSSION

Here, we report that the microbiome of a field soil contributes to suppression of Striga infection of sorghum roots via disruption of host-parasite signaling and modulation of host root anatomy. Root exudates from sorghum grown in the Striga-suppressive soil did not affect Striga seed germination or strigolactone levels but did significantly reduce haustorium formation, a phenotype that was associated with reduced levels of four key HIFs (syringic acid, vanillic acid, DMBQ, acetosyringone) (Figures 1 and 2). These results indicate that host-parasite signaling was disrupted at the level of haustoria formation via HIF degradation. Furthermore, more aerenchyma and endodermal suberin was detected in roots of sorghum grown in the presence of the soil microbiome (Figure 3). These structural changes in root cellular anatomy likely affect the ability of Striga to penetrate the root. It is not known if progression of Striga through the root tissue requires a touch or mechanical stimulus from adjacent host tissue, but air-filled gaps in the cortex could likely disrupt this. Aerenchyma have also been associated with drought tolerance due to reduced metabolic and energy requirements.[33] An alternative hypothesis is that the parasitic plant may similarly sense a lack of metabolic activity and not continue with parasitization. A suberized endodermis can act as a physical barrier to Striga, preventing it from reaching the xylem. Physical barriers, consisting of lignin, callose, phenolic compounds, or silica, can provide partial resistance in several host species and to several parasite species.[14,34–41] These barriers can be innate or induced upon infection with a parasitic plant.[42] Establishment of sorghum lines with constitutive suberin and aerenchyma production could provide support for these hypotheses. Despite prior reports that RSA is associated with Striga resistance,[43–45] we observed no effect of the soil microbiome on RSA traits measured here (Figure 3A).

To begin to validate the role of specific microbial taxa in HIF degradation and induction of suberization and aerenchyma, we

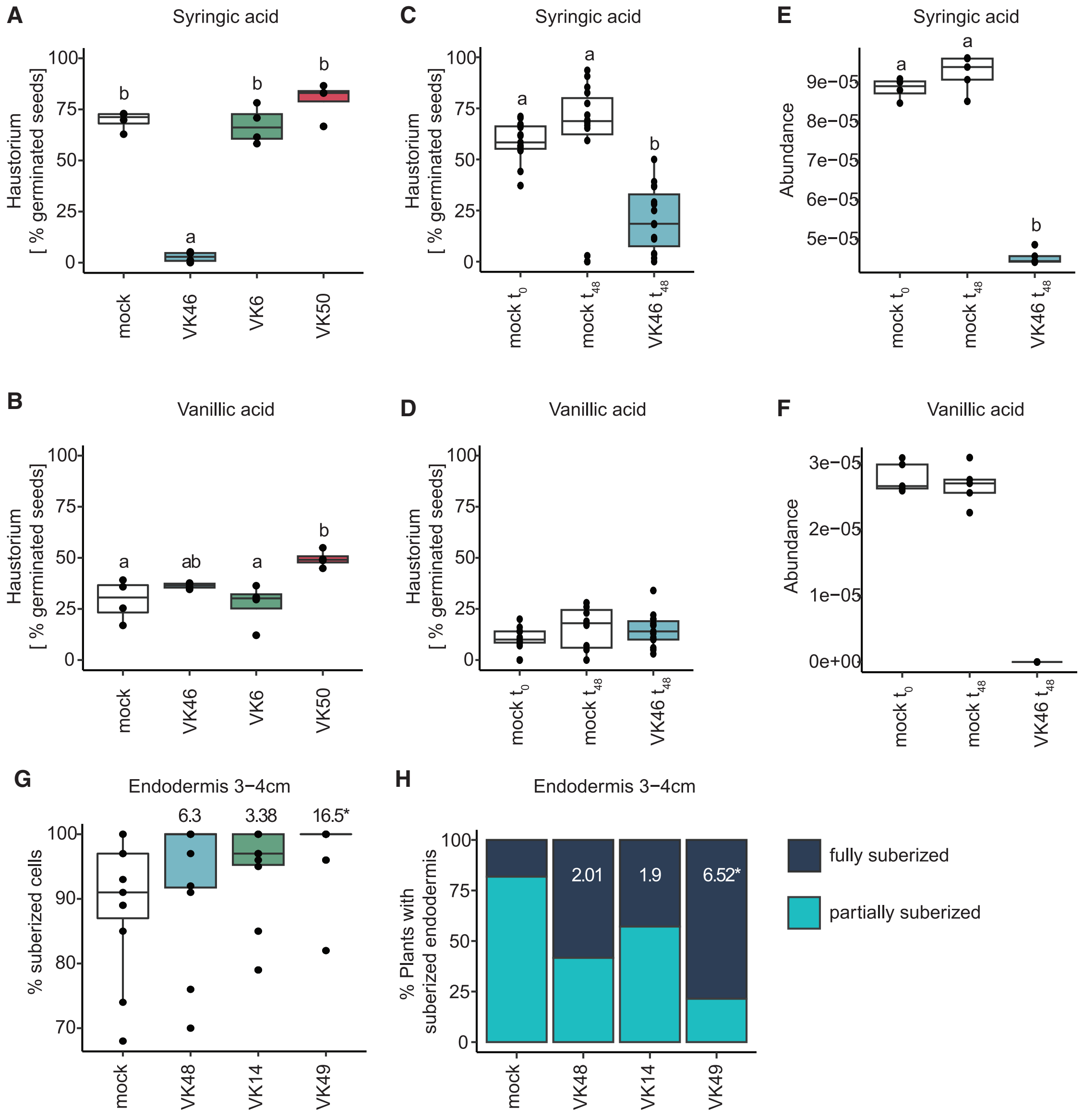

Figure 4. Individual bacterial isolates reduce haustorium formation and endodermal suberization

(A and B) Percentage of germinated Striga seeds that developed haustorium in the presence of (A) 100 μM syringic acid and (B) 50 μM vanillic acid incubated with *Pseudomonas* isolates VK46, VK6, and VK50. Sterile medium used to grow the bacteria was used as a mock treatment.

(C–F) The effect of VK46 on (C and D) haustorium formation and (E and F) HIF levels after incubation with (C and E) 100 μM syringic acid and (D and F) 50 μM vanillic acid. Measurements were made at the time of application to the Striga seeds and after 48 h of incubation. Statistical differences were tested with a one-way ANOVA and Tukey post hoc test. Letters denote significant differences between treatments.

(G and H) Percentage of suberized cells in the endodermis (G) and percentage of plants with a fully and partially suberized endodermis (H) within 3–4 cm from the root tip upon inoculation with *Arthrobacter* isolates VK48, VK14, and VK49. Numbers in (G) and (H) denote odds ratio, and asterisks denote significant difference between plants inoculated with each isolate and the mock-treated plants determined by the least squares method. Boxplots (A–G) denote the span from the 25th to 75th percentile and are centered to the data median.

tested a small number of bacterial isolates prioritized by GJAM analyses. It should be emphasized that the selection of the bacterial taxa was based on 16S amplicon sequence similarity, with only a minor 16S amplicon fragment as the template in the sequence alignment. In other words, our selection of the isolates was limited, as it does not cover the full 16S sequence and, more importantly, does not use other taxonomic and functional markers of these bacterial taxa. Nevertheless, we did show that some of the prioritized bacterial isolates were able to degrade specific HIFs or to induce suberization (Figure 4). These results provide proof of concept, at least in part, that specific bacterial taxa of the soil microbiome can mediate post-germination (haustorium formation) and post-attachment resistance, preventing Striga from penetrating through the root to establish a vascular connection.

The potential of specific microbial species to degrade phenolic compounds, including vanillic and syringic acid, has been previously reported.[46,47] While it was mostly studied in the context of lignin degradation, here, we show that this activity can also be leveraged to reduce Striga parasitism. *Pseudomonas* isolate VK46 was isolated from the soil with reduced HIFs within root exudates and inhibited haustorium formation in the presence of syringic, but not vanillic, acid (Figures 4A and 4B). Such selectivity toward some phenolic compounds among distinct microbes has been reported previously.[48]

Some pathogens reduce suberization of the endodermis that otherwise blocks their entry to plant vasculature.[49] Several commensal bacteria can lower suberin content in Arabidopsis.[50] This negative effect of microbes on Arabidopsis suberization contrasts with our observations in sorghum, where multiple microbial taxa were associated positively with endodermal suberin content (Data S5). These inter-species discrepancies could be caused by the presence of the exodermis in sorghum—an additional cell type where suberin can be deposited. Moreover, sorghum might assemble microbial communities different from those in Arabidopsis. Out of 41 endophytes tested in Arabidopsis, the majority reduced the fully suberized root zone, but six isolates promoted early endodermal suberization.[50] This suggests that some overlap in suberin-inducing bacterial function might exist between sorghum and Arabidopsis.

Increased suberization could alternatively result from microbes affecting the nutritional status of the plant (indirect effect) or by microbes promoting the production of suberin precursors by the plant (direct effect). The latter has been observed in sorghum grown under drought (thus, conditions promoting suberin deposition in roots),[51] where increased production of glycerol-3-phosphate coincides with enrichment of monoderm bacteria, like Actinobacteria.[52] It has been hypothesized that since monoderms use glycerol-3-phosphate to assemble their cell walls, they might induce its production in sorghum roots.[53] It is thus plausible that plants can also use this glycerol-3-phosphate as a substrate for suberin biosynthesis. Indeed, we observed Actinobacteria in our top-ranked 100 taxa associated with suberization as well as upregulation of two glycerol phosphate transferases genes (*SbGPAT4/8* and *SbGPAT5/7*) in sorghum roots exposed to natural soil (Figures 3J and 3K).

To the best of our knowledge, microbe-mediated induction of aerenchyma has not been reported. Ethylene induces aerenchyma formation,[54] and several ethylene-related genes were regulated by the Striga-suppressive soil microbiome (Figure 3M; Data S2). Microbes have been shown to interfere with plant ethylene signaling, and both ethylene-producing and ethylene-degrading bacterial strains have been found.[55] A reasonable hypothesis, therefore, is that ethylene-inducing microbes may induce aerenchyma that then restricts Striga entry into the root vasculature.

The known modes of pre- and post-attachment resistance in host species usually provide only partial protection. Likewise, each of the isolates identified here on their own would likely provide little or limited protection against Striga. Combining microbes in a consortium (also referred to as synthetic communities) that could induce multiple traits could provide a higher level of resistance. These microbial consortia should be assembled based on the extensive metagenomic sequencing and targeted identification and isolation of their members from the soils native to areas of their application. Bacterial taxa can then be used to prioritize the selection of microbial isolates from collections established from Striga-infested local soils in a targeted screen for resistance-associated phenotypes (HIF degradation, increase in aerenchyma and suberin content). Functional markers associated with (1) the potential to degrade syringic acid or (2) upregulation of genes associated with the increase in aerenchyma content and suberization will further facilitate the targeted screens. While the host genotype dependency of these identified mechanisms and their robustness to environmental conditions typical to areas where sorghum is grown still need to be addressed, this work lays the foundation for designing a multi-member microbial consortium that suppresses haustorium formation and induces diverse structural barriers in roots to collectively reduce Striga parasitism.

Limitations of the study

Our work identifies three modes of microbiome-based alteration of Sorghum host traits associated with suppression of Striga infection: degradation of HIFs, changes to root endodermal suberin, and induction of aerenchyma. Bacterial isolate VK46 degraded syringic and vanillic acid in an *in vitro* assay. Sorghum root exudates contain additional HIFs; thus, the efficiency of VK46 would likely need to be combined with other HIF-degrading isolates to reduce Striga infection *in planta*. While the microbiome-induced promotion of aerenchyma and endodermal suberization coincides with Striga infection suppression, we still lack genetic resources to ultimately prove that suberin forms a physical barrier to Striga and that the presence of aerenchyma limits parasite penetration through the root tissue. Furthermore, our selection of candidate bacterial taxa for functional validation was constrained because it did not encompass the entire 16S sequence and did not incorporate other taxonomic and functional markers associated with these bacterial taxa.

STAR★METHODS

Detailed methods are provided in the online version of this paper and include the following:

- KEY RESOURCES TABLE
- RESOURCE AVAILABILITY
  - ○ Lead contact
  - ○ Materials availability
  - ○ Data and code availability
- EXPERIMENTAL MODEL AND STUDY PARTICIPANT DETAILS
  - ○ Soil material
  - ○ Plant material and growth conditions – soil ‘‘plug’’ assay
- METHOD DETAILS
  - ○ Striga infection quantification
  - ○ Exudate collection and profiling
  - ○ Prediction of microbial degradation products
  - ○ *In vitro* germination and haustorium formation assay

    - Root system architecture phenotyping
    - Root cellular anatomy phenotyping
    - RNA-seq library preparation
    - Microbial community analysis
    - Haustorium formation and HIF quantification with individual bacterial isolates
    - Sorghum inoculation with individual bacterial isolates
    - Aerenchyma quantification in the presence of individual isolates
    - Suberin quantification -– individual isolates assay
- QUANTIFICATION AND STATISTICAL ANALYSIS
    - RNA-seq read processing and differential expression analysis
    - Gene orthology identification
    - Microbial community analysis
    - Identification of microbial candidates associated with reduced Striga infection

SUPPLEMENTAL INFORMATION

Supplemental information can be found online at https://doi.org/10.1016/j.celrep.2024.113971.

ACKNOWLEDGMENTS

We would like to thank Ludek Tikovsky and Harold Lemereis for their assistance in the greenhouse, Angelica Guercio for help with quantification of root cellular anatomy, and Daniel Runcie for the advice on the statistical analyses. We are grateful to Wim van der Putten and Viola Kurm for providing the bacterial isolates from Clue Field soil. D.K., B.T., M.Z.S., T. Taylor, A.W., H.E.V., F.D.-A., M.Z., J.D., T. Tessema, E.E.K., J.M.R., H.B., and S.M.B. gratefully acknowledge support from the Bill and Melinda Gates Foundation (Seattle, WA, USA) via grant OPP1082853 "RSM Systems Biology for Sorghum." S.M.B. was partially funded by an HHMI Faculty Scholar grant and IOS-1856749 and IOS-211980. A.B. was in part supported by the USDOE ARPA-E ROOTS Award no. DE-AR0000821 by the NSF CAREER Award no. 1845760. Any opinions, findings, and conclusions or recommendations expressed in this material are those of the author(s) and do not necessarily reflect those of the National Science Foundation.

AUTHOR CONTRIBUTIONS

Conceptualization, D.K., B.T., M.Z.S., J.M.R., H.B., and S.M.B.; data curation, D.K.; formal analysis, D.K., B.T., M.F.A.L., and A.B.; funding acquisition, J.M.R., H.B., and S.M.B.; investigation, D.K., B.T., M.S., T. Taylor, A.W., H.E.V., D.R., Z.M., F.D.-A., M.S., A.J.C., J.D., and S.M.B.; methodology, D.K., B.T., M.Z.S., M.F.A.L., and A.B.; project administration, E.E.K., J.M.R., H.B., and S.M.B.; software, D.K., M.F.A.L., and A.B.; supervision, D.K., B.T., E.E.K., J.M.R., H.B., and S.M.B.; resources, T. Tessema and J.M.R.; validation, D.K., B.T., and M.Z.S.; visualization, D.K.; writing – original draft, D.K. and S.M.B.

DECLARATION OF INTERESTS

A patent application relating to this work has been filed.

Received: January 26, 2023
Revised: January 17, 2024
Accepted: February 29, 2024
Published: March 26, 2024

REFERENCES

1. Harris-Shultz, K.R., Hayes, C.M., and Knoll, J.E. (2019). Mapping QTLs and Identification of Genes Associated with Drought Resistance in Sorghum. Methods Mol. Biol. *1931*, 11–40. https://doi.org/10.1007/978-1-4939-9039-9_2.
2. Gurney, A.L., Press, M.C., and Scholes, J.D. (1999). Infection time and density influence the response of sorghum to the parasitic angiosperm Striga hermonthica. New Phytol. *143*, 573–580. https://doi.org/10.1046/j.1469-8137.1999.00467.x.
3. Runo, S., and Kuria, E.K. (2018). Habits of a highly successful cereal killer, Striga. PLoS Pathog. *14*, e1006731. https://doi.org/10.1371/journal.ppat.1006731.
4. De Groote, H., Wangare, L., Kanampiu, F., Odendo, M., Diallo, A., Karaya, H., and Friesen, D. (2008). The potential of a herbicide resistant maize technology for Striga control in Africa. Agr Syst *97*, 83–94. https://doi.org/10.1016/j.agsy.2007.12.003.
5. Rodenburg, J., Demont, M., Zwart, S.J., and Bastiaans, L. (2016). Parasitic weed incidence and related economic losses in rice in Africa. Agric. Ecosyst. Environ. *235*, 306–317. https://doi.org/10.1016/j.agee.2016.10.020.
6. Akiyama, K., Matsuzaki, K.i., and Hayashi, H. (2005). Plant sesquiterpenes induce hyphal branching in arbuscular mycorrhizal fungi. Nature *435*, 824–827. https://doi.org/10.1038/nature03608.
7. Bouwmeester, H., Li, C., Thiombiano, B., Rahimi, M., and Dong, L. (2021). Adaptation of the parasitic plant lifecycle: germination is controlled by essential host signaling molecules. Plant Physiol. *185*, 1292–1308. https://doi.org/10.1093/plphys/kiaa066.
8. Awad, A.A., Sato, D., Kusumoto, D., Kamioka, H., Takeuchi, Y., and Yoneyama, K. (2006). Characterization of strigolactones, germination stimulants for the root parasitic plants Striga and Orobanche, produced by maize, millet and sorghum. Plant Growth Regul. *48*, 221–227. https://doi.org/10.1007/s10725-006-0009-3.
9. Bandaranayake, P.C.G., Filappova, T., Tomilov, A., Tomilova, N.B., Jamison-McClung, D., Ngo, Q., Inoue, K., and Yoder, J.I. (2010). A single-electron reducing quinone oxidoreductase is necessary to induce haustorium development in the root parasitic plant Triphysaria. Plant Cell *22*, 1404–1419. https://doi.org/10.1105/tpc.110.074831.
10. Yoshida, S., Cui, S., Ichihashi, Y., and Shirasu, K. (2016). The Haustorium, a Specialized Invasive Organ in Parasitic Plants. Annu. Rev. Plant Biol. *67*, 643–667. https://doi.org/10.1146/annurev-arplant-043015-111702.
11. Kuijt, J. (1969). The Biology of Parasitic Flowering Plants (University of California Press).
12. GRAVES, J.D., PRESS, M.C., and STEWART, G.R. (1989). A carbon balance model of the sorghum-Striga hermonthica host-parasite association. Plant Cell Environ. *12*, 101–107. https://doi.org/10.1111/j.1365-3040.1989.tb01921.x.
13. Gobena, D., Shimels, M., Rich, P.J., Ruyter-Spira, C., Bouwmeester, H., Kanuganti, S., Mengiste, T., and Ejeta, G. (2017). Mutation in sorghum LOW GERMINATION STIMULANT 1 alters strigolactones and causes Striga resistance. Proc. Natl. Acad. Sci. USA *114*, 4471–4476. https://doi.org/10.1073/pnas.1618965114.
14. Mbuvi, D.A., Masiga, C.W., Kuria, E., Masanga, J., Wamalwa, M., Mohamed, A., Odeny, D.A., Hamza, N., Timko, M.P., and Runo, S. (2017). Novel Sources of Witchweed (Striga) Resistance from Wild Sorghum Accessions. Front. Plant Sci. *8*, 116. https://doi.org/10.3389/fpls.2017.00116.
15. Goldwasser, Y., and Rodenburg, J. (2013). Integrated Agronomic Management of Parasitic Weed Seed Banks. In Parasitic Orobanchaceae, D.M. Joel, J. Gressel, and L.J. Musselman, eds. (Springer Berlin Heidelberg), pp. 393–413. https://doi.org/10.1007/978-3-642-38146-1_22.
16. Raaijmakers, J.M., and Mazzola, M. (2016). ECOLOGY. Soil immune responses. Science *352*, 1392–1393. https://doi.org/10.1126/science.aaf3252.

17. Weller, D.M., Raaijmakers, J.M., Gardener, B.B.M., and Thomashow, L.S. (2002). Microbial populations responsible for specific soil suppressiveness to plant pathogens. Annu. Rev. Phytopathol. *40*, 309–348. https://doi.org/10.1146/annurev.phyto.40.030402.110010.

18. Gómez Expósito, R., de Bruijn, I., Postma, J., and Raaijmakers, J.M. (2017). Current Insights into the Role of Rhizosphere Bacteria in Disease Suppressive Soils. Front. Microbiol. *8*, 2529. https://doi.org/10.3389/fmicb.2017.02529.

19. Masteling, R., Lombard, L., de Boer, W., Raaijmakers, J.M., and Dini-Andreote, F. (2019). Harnessing the microbiome to control plant parasitic weeds. Curr. Opin. Microbiol. *49*, 26–33. https://doi.org/10.1016/j.mib.2019.09.006.

20. Ahonsi, M.O., Berner, D.K., Emechebe, A.M., and Lagoke, S.T. (2002). Selection of rhizobacterial strains for suppression of germination of Striga hermonthica (Del.) Benth. seeds. Biol. Control *24*, 143–152. https://doi.org/10.1016/s1049-9644(02)00019-1.

21. Nzioki, H.S., Oyosi, F., Morris, C.E., Kaya, E., Pilgeram, A.L., Baker, C.S., and Sands, D.C. (2016). Striga Biocontrol on a Toothpick: A Readily Deployable and Inexpensive Method for Smallholder Farmers. Front. Plant Sci. *7*, 1121. https://doi.org/10.3389/fpls.2016.01121.

22. Schlemper, T.R., Leite, M.F.A., Lucheta, A.R., Shimels, M., Bouwmeester, H.J., van Veen, J.A., and Kuramae, E.E. (2017). Rhizobacterial community structure differences among sorghum cultivars in different growth stages and soils. FEMS Microbiol. Ecol. *93*. https://doi.org/10.1093/femsec/fix096.

23. Kawa, D., Taylor, T., Thiombiano, B., Musa, Z., Vahldick, H.E., Walmsley, A., Bucksch, A., Bouwmeester, H., and Brady, S.M. (2021). Characterization of growth and development of sorghum genotypes with differential susceptibility to Striga hermonthica. J. Exp. Bot. *72*, 7970–7983. https://doi.org/10.1093/jxb/erab380.

24. Cui, S., Wada, S., Tobimatsu, Y., Takeda, Y., Saucet, S.B., Takano, T., Umezawa, T., Shirasu, K., and Yoshida, S. (2018). Host lignin composition affects haustorium induction in the parasitic plants Phtheirospermum japonicum and Striga hermonthica. New Phytol. *218*, 710–723. https://doi.org/10.1111/nph.15033.

25. Djoumbou-Feunang, Y., Fiamoncini, J., Gil-de-la-Fuente, A., Greiner, R., Manach, C., and Wishart, D.S. (2019). BioTransformer: a comprehensive computational tool for small molecule metabolism prediction and metabolite identification. J. Cheminform. *11*, 2. https://doi.org/10.1186/s13321-018-0324-5.

26. Gou, J.Y., Yu, X.H., and Liu, C.J. (2009). A hydroxycinnamoyltransferase responsible for synthesizing suberin aromatics in Arabidopsis. Proc. Natl. Acad. Sci. USA *106*, 18855–18860. https://doi.org/10.1073/pnas.0905555106.

27. Molina, I., Li-Beisson, Y., Beisson, F., Ohlrogge, J.B., and Pollard, M. (2009). Identification of an Arabidopsis feruloyl-coenzyme A transferase required for suberin synthesis. Plant Physiol. *151*, 1317–1328. https://doi.org/10.1104/pp.109.144907.

28. Cantó-Pastor, A., Kajala, K., Shaar-Moshe, L., Manzano, C., Timilsena, P., De Bellis, D., Gray, S., Holbein, J., Yang, H., Mohammad, S., et al. (2024). A suberized exodermis is required for tomato drought tolerance. Nat. Plants *10*, 118–130. https://doi.org/10.1038/s41477-023-01567-x.

29. Beisson, F., Li, Y., Bonaventure, G., Pollard, M., and Ohlrogge, J.B. (2007). The acyltransferase GPAT5 is required for the synthesis of suberin in seed coat and root of Arabidopsis. Plant Cell *19*, 351–368. https://doi.org/10.1105/tpc.106.048033.

30. Yadav, V., Molina, I., Ranathunge, K., Castillo, I.Q., Rothstein, S.J., and Reed, J.W. (2014). ABCG transporters are required for suberin and pollen wall extracellular barriers in Arabidopsis. Plant Cell *26*, 3569–3588. https://doi.org/10.1105/tpc.114.129049.

31. Kurm, V., van der Putten, W.H., and Hol, W.H.G. (2019). Cultivation-success of rare soil bacteria is not influenced by incubation time and growth medium. PLoS One *14*, e0210073. https://doi.org/10.1371/journal.pone.0210073.

32. Kajala, K., Gouran, M., Shaar-Moshe, L., Mason, G.A., Rodriguez-Medina, J., Kawa, D., Pauluzzi, G., Reynoso, M., Canto-Pastor, A., Manzano, C., et al. (2021). Innovation, conservation, and repurposing of gene function in root cell type development. Cell *184*, 3333–3348.e19. https://doi.org/10.1016/j.cell.2021.04.024.

33. Zhu, J., Brown, K.M., and Lynch, J.P. (2010). Root cortical aerenchyma improves the drought tolerance of maize (Zea mays L.). Plant Cell Environ. *33*, 740–749. https://doi.org/10.1111/j.1365-3040.2009.02099.x.

34. Maiti, R.K., Ramaiah, K.V., Bisen, S.S., and Chidley, V.L. (1984). A Comparative Study of the Haustorial Development of Striga asiatica (L.) Kuntze on Sorghum Cultivars. Ann. Bot. *54*, 447–457. https://doi.org/10.1093/oxfordjournals.aob.a086816.

35. Rubiales, D., PÈrez-de-Luque, A., Joel, D.M., Alc·ntara, C., and Sillero, J.C. (2003). Characterization of Resistance in Chickpea to Crenate Broomrape (Orobanche Crenata). Weed Science *51*, 702–707. https://doi.org/10.1614/P2002-151.

36. Gurney, A.L., Slate, J., Press, M.C., and Scholes, J.D. (2006). A novel form of resistance in rice to the angiosperm parasite Striga hermonthica. New Phytol. *169*, 199–208. https://doi.org/10.1111/j.1469-8137.2005.01560.x.

37. Pérez-de-Luque, A., Lozano, M.D., Cubero, J.I., González-Melendi, P., Risueño, M.C., and Rubiales, D. (2006). Mucilage production during the incompatible interaction between Orobanche crenata and Vicia sativa. J. Exp. Bot. *57*, 931–942. https://doi.org/10.1093/jxb/erj078.

38. Yoshida, S., and Shirasu, K. (2009). Multiple layers of incompatibility to the parasitic witchweed, Striga hermonthica. New Phytol. *183*, 180–189. https://doi.org/10.1111/j.1469-8137.2009.02840.x.

39. Cissoko, M., Boisnard, A., Rodenburg, J., Press, M.C., and Scholes, J.D. (2011). New Rice for Africa (NERICA) cultivars exhibit different levels of post-attachment resistance against the parasitic weeds Striga hermonthica and Striga asiatica. New Phytol. *192*, 952–963. https://doi.org/10.1111/j.1469-8137.2011.03846.x.

40. Mutuku, J.M., Cui, S., Hori, C., Takeda, Y., Tobimatsu, Y., Nakabayashi, R., Mori, T., Saito, K., Demura, T., Umezawa, T., et al. (2019). The Structural Integrity of Lignin Is Crucial for Resistance against Striga hermonthica Parasitism in Rice. Plant Physiol. *179*, 1796–1809. https://doi.org/10.1104/pp.18.01133.

41. Mutinda, S.M., Masanga, J., Mutuku, J.M., Runo, S., and Alakonya, A. (2018). KSTP 94, an Open-pollinated Maize Variety Has Postattachment Resistance to Purple Witchweed (Striga hermonthica). Weed Sci. *66*, 525–529. https://doi.org/10.1017/wsc.2018.24.

42. Kawa, D., and Brady, S.M. (2022). Root cell types as an interface for biotic interactions. Trends Plant Sci. *27*, 1173–1186. https://doi.org/10.1016/j.tplants.2022.06.003.

43. Cherif-Ari, O., Housley, T.L., and Ejeta, G. (1990). Sorghum root length density and the potential for avoiding striga parasitism. Plant Soil *121*, 67–72. https://doi.org/10.1007/bf00013098.

44. Abate, M., Hussien, T., Bayu, W., Reda, F., and Vurro, M. (2017). Diversity in root traits of sorghum genotypes in response to Striga hermonthica infestation. Weed Res. *57*, 303–313. https://doi.org/10.1111/wre.12262.

45. Burridge, J.D., Schneider, H.M., Huynh, B.L., Roberts, P.A., Bucksch, A., and Lynch, J.P. (2017). Genome-wide association mapping and agronomic impact of cowpea root architecture. Theor. Appl. Genet. *130*, 419–431. https://doi.org/10.1007/s00122-016-2823-y.

46. Wang, J., Liang, J., and Gao, S. (2018). Biodegradation of Lignin Monomers Vanillic, p-Coumaric, and Syringic Acid by the Bacterial Strain, Sphingobacterium sp. HY-H. Curr. Microbiol. *75*, 1156–1164. https://doi.org/10.1007/s00284-018-1504-2.

47. Oshlag, J.Z., Ma, Y., Morse, K., Burger, B.T., Lemke, R.A., Karlen, S.D., Myers, K.S., Donohue, T.J., and Noguera, D.R. (2020). Anaerobic Degradation of Syringic Acid by an Adapted Strain of Rhodopseudomonas palustris. Appl. Environ. Microbiol. *86*, e01888-19. https://doi.org/10.1128/AEM.01888-19.

48. Margesin, R., Volgger, G., Wagner, A.O., Zhang, D., and Poyntner, C. (2021). Biodegradation of lignin monomers and bioconversion of ferulic acid to vanillic acid by Paraburkholderia aromaticivorans AR20-38 isolated from Alpine forest soil. Appl. Microbiol. Biotechnol. *105*, 2967–2977. https://doi.org/10.1007/s00253-021-11215-z.

49. Fröschel, C., Komorek, J., Attard, A., Marsell, A., Lopez-Arboleda, W.A., Le Berre, J., Wolf, E., Geldner, N., Waller, F., Korte, A., and Dröge-Laser, W. (2021). Plant roots employ cell-layer-specific programs to respond to pathogenic and beneficial microbes. Cell Host Microbe *29*, 299–310.e7. https://doi.org/10.1016/j.chom.2020.11.014.

50. Salas-González, I., Reyt, G., Flis, P., Custódio, V., Gopaulchan, D., Bakhoum, N., Dew, T.P., Suresh, K., Franke, R.B., Dangl, J.L., et al. (2021). Coordination between microbiota and root endodermis supports plant mineral nutrient homeostasis. Science *371*, eabd0695. https://doi.org/10.1126/science.abd0695.

51. Baxter, I., Hosmani, P.S., Rus, A., Lahner, B., Borevitz, J.O., Muthukumar, B., Mickelbart, M.V., Schreiber, L., Franke, R.B., and Salt, D.E. (2009). Root suberin forms an extracellular barrier that affects water relations and mineral nutrition in Arabidopsis. PLoS Genet. *5*, e1000492. https://doi.org/10.1371/journal.pgen.1000492.

52. Xu, L., Naylor, D., Dong, Z., Simmons, T., Pierroz, G., Hixson, K.K., Kim, Y.M., Zink, E.M., Engbrecht, K.M., Wang, Y., et al. (2018). Drought delays development of the sorghum root microbiome and enriches for monoderm bacteria. Proc. Natl. Acad. Sci. USA *115*, E4284–E4293. https://doi.org/10.1073/pnas.1717308115.

53. Xu, L., and Coleman-Derr, D. (2019). Causes and consequences of a conserved bacterial root microbiome response to drought stress. Curr. Opin. Microbiol. *49*, 1–6. https://doi.org/10.1016/j.mib.2019.07.003.

54. Yamauchi, T., Shimamura, S., Nakazono, M., and Mochizuki, T. (2013). Aerenchyma formation in crop species: A review. Field Crops Res. *152*, 8–16. https://doi.org/10.1016/j.fcr.2012.12.008.

55. Ravanbakhsh, M., Sasidharan, R., Voesenek, L.A.C.J., Kowalchuk, G.A., and Jousset, A. (2018). Microbial modulation of plant ethylene signaling: ecological and evolutionary consequences. Microbiome *6*, 52. https://doi.org/10.1186/s40168-018-0436-1.

56. Floková, K., Shimels, M., Andreo Jimenez, B., Bardaro, N., Strnad, M., Novák, O., and Bouwmeester, H.J. (2020). An improved strategy to analyse strigolactones in complex sample matrices using UHPLC-MS/MS. Plant Methods *16*, 125. https://doi.org/10.1186/s13007-020-00669-3.

57. Das, A., Schneider, H., Burridge, J., Ascanio, A.K.M., Wojciechowski, T., Topp, C.N., Lynch, J.P., Weitz, J.S., and Bucksch, A. (2015). Digital imaging of root traits (DIRT): a high-throughput computing and collaboration platform for field-based root phenomics. Plant Methods *11*, 51. https://doi.org/10.1186/s13007-015-0093-3.

58. Bucksch, A. (2014). A practical introduction to skeletons for the plant sciences. Appl. Plant Sci. *2*, apps.1400005. https://doi.org/10.3732/apps.1400005.

59. Bucksch, A., Burridge, J., York, L.M., Das, A., Nord, E., Weitz, J.S., and Lynch, J.P. (2014). Image-based high-throughput field phenotyping of crop roots. Plant Physiol. *166*, 470–486. https://doi.org/10.1104/pp.114.243519.

60. Lundberg, D.S., Lebeis, S.L., Paredes, S.H., Yourstone, S., Gehring, J., Malfatti, S., Tremblay, J., Engelbrektson, A., Kunin, V., Del Rio, T.G., et al. (2012). Defining the core Arabidopsis thaliana root microbiome. Nature *488*, 86–90. https://doi.org/10.1038/nature11237.

61. Van Noordwijk, M., and Brouwer, G. (1988). Quantification of air-filled root porosity: A comparison of two methods. Plant Soil *111*, 255–258.

62. Davis, M.P.A., van Dongen, S., Abreu-Goodger, C., Bartonicek, N., and Enright, A.J. (2013). Kraken: a set of tools for quality control and analysis of high-throughput sequence data. Methods *63*, 41–49. https://doi.org/10.1016/j.ymeth.2013.06.027.

63. McCormick, R.F., Truong, S.K., Sreedasyam, A., Jenkins, J., Shu, S., Sims, D., Kennedy, M., Amirebrahimi, M., Weers, B.D., McKinley, B., et al. (2018). The Sorghum bicolor reference genome: improved assembly, gene annotations, a transcriptome atlas, and signatures of genome organization. Plant J. *93*, 338–354. https://doi.org/10.1111/tpj.13781.

64. Dobin, A., Davis, C.A., Schlesinger, F., Drenkow, J., Zaleski, C., Jha, S., Batut, P., Chaisson, M., and Gingeras, T.R. (2013). STAR: ultrafast universal RNA-seq aligner. Bioinformatics *29*, 15–21. https://doi.org/10.1093/bioinformatics/bts635.

65. Robinson, M.D., McCarthy, D.J., and Smyth, G.K. (2010). edgeR: a Bioconductor package for differential expression analysis of digital gene expression data. Bioinformatics *26*, 139–140. https://doi.org/10.1093/bioinformatics/btp616.

66. Ritchie, M.E., Phipson, B., Wu, D., Hu, Y., Law, C.W., Shi, W., and Smyth, G.K. (2015). limma powers differential expression analyses for RNA-sequencing and microarray studies. Nucleic Acids Res. *43*, e47. https://doi.org/10.1093/nar/gkv007.

67. Rajhi, I., Yamauchi, T., Takahashi, H., Nishiuchi, S., Shiono, K., Watanabe, R., Mliki, A., Nagamura, Y., Tsutsumi, N., Nishizawa, N.K., and Nakazono, M. (2011). Identification of genes expressed in maize root cortical cells during lysigenous aerenchyma formation using laser microdissection and microarray analyses. New Phytol. *190*, 351–368. https://doi.org/10.1111/j.1469-8137.2010.03535.x.

68. Takahashi, H., Yamauchi, T., Rajhi, I., Nishizawa, N.K., and Nakazono, M. (2015). Transcript profiles in cortical cells of maize primary root during ethylene-induced lysigenous aerenchyma formation under aerobic conditions. Ann. Bot. *115*, 879–894. https://doi.org/10.1093/aob/mcv018.

69. Arora, K., Panda, K.K., Mittal, S., Mallikarjuna, M.G., Rao, A.R., Dash, P.K., and Thirunavukkarasu, N. (2017). RNAseq revealed the important gene pathways controlling adaptive mechanisms under waterlogged stress in maize. Sci. Rep. *7*, 10950. https://doi.org/10.1038/s41598-017-10561-1.

70. Edgar, R.C. (2013). UPARSE: highly accurate OTU sequences from microbial amplicon reads. Nat. Methods *10*, 996–998. https://doi.org/10.1038/nmeth.2604.

71. Edgar, R.C., Haas, B.J., Clemente, J.C., Quince, C., and Knight, R. (2011). UCHIME improves sensitivity and speed of chimera detection. Bioinformatics *27*, 2194–2200. https://doi.org/10.1093/bioinformatics/btr381.

72. Wang, Q., Garrity, G.M., Tiedje, J.M., and Cole, J.R. (2007). Naive Bayesian classifier for rapid assignment of rRNA sequences into the new bacterial taxonomy. Appl. Environ. Microbiol. *73*, 5261–5267. https://doi.org/10.1128/AEM.00062-07.

73. Quast, C., Pruesse, E., Yilmaz, P., Gerken, J., Schweer, T., Yarza, P., Peplies, J., and Glöckner, F.O. (2013). The SILVA ribosomal RNA gene database project: improved data processing and web-based tools. Nucleic Acids Res. *41*, D590–D596. https://doi.org/10.1093/nar/gks1219.

74. McMurdie, P.J., and Holmes, S. (2013). phyloseq: an R package for reproducible interactive analysis and graphics of microbiome census data. PLoS One *8*, e61217. https://doi.org/10.1371/journal.pone.0061217.

75. Clark, J.S., Nemergut, D., Seyednasrollah, B., Turner, P.J., and Zhang, S. (2017). Generalized joint attribute modeling for biodiversity analysis: median-zero, multivariate, multifarious data. Ecol. Monogr. *87*, 34–56. https://doi.org/10.1002/ecm.1241.

76. Leite, M.F., and Kuramae, E.E. (2020). You must choose, but choose wisely: Model-based approaches for microbial community analysis. Soil Biol. Biochem. *151*, 108042. https://doi.org/10.1016/j.soilbio.2020.108042.

77. Pollock, L.J., Tingley, R., Morris, W.K., Golding, N., O'Hara, R.B., Parris, K.M., Vesk, P.A., McCarthy, M.A., and McPherson, J. (2014). Understanding co-occurrence by modelling species simultaneously with a Joint Species Distribution Model (JSDM). Methods Ecol. Evol. *5*, 397–406. https://doi.org/10.1111/2041-210x.12180.

CellPress
OPEN ACCESS

STAR★METHODS

KEY RESOURCES TABLE

REAGENT or RESOURCE	SOURCE	IDENTIFIER
**Bacterial and virus strains**		
VK6	Kurm et al.[31]	N/A
VK14	Kurm et al.[31]	N/A
VK15	Kurm et al.[31]	N/A
VK46	Kurm et al.[31]	N/A
VK48	Kurm et al.[31]	N/A
VK49	Kurm et al.[31]	N/A
VK50	Kurm et al.[31]	N/A
VK51	Kurm et al.[31]	N/A
**Chemicals, peptides, and recombinant proteins**		
racGR24 (racemic mix of enantiomers $GR24^{5DS}$ and $GR24^{ent-5DS}$	StrigoLab, Italy	Cas# 76974-79-3; Batch CC1
DMBQ, 2,6-methoxy-1,4-benzoquinone 97%	Merck Sigma Aldrich Chemie B.V	Cat# 428566
Syringic acid, 95%	Merck Sigma Aldrich Chemie B.V	Cat# S6881
Vanillic acid, 98%	Alfa Aesar	Cat# A12074.14
Fluorol yellow	Santa Cruz Biotech.	Cat# sc-215052
**Critical commercial assays**		
QuantSeq 3′ mRNA-Seq Library Prep Kit	Lexogen	Cat# 015.96
RNaesy Plus Mini kit	Qiagen	Cat# 74134
MoBio PowerSoil DNA	Qiagen	Cat# 12888-100
**Deposited data**		
RNA-seq sorghum roots	This study	NCBI: GSE 216351
Amplicon sequences – microbiome profiling	This study	ENA: PRJEB57848
VK6 - isolate 16S amplicon sequence	This study	NCBI: KX503329
VK14 - isolate 16S amplicon sequence	This study	NCBI: KX503337
VK15 - isolate 16S amplicon sequence	This study	NCBI: KX503338
VK46 - isolate 16S amplicon sequence	This study	NCBI: KX503369
VK48 - isolate 16S amplicon sequence	This study	NCBI: OP954904
VK49 - isolate 16S amplicon sequence	This study	NCBI: OP959794
VK50 - isolate 16S amplicon sequence	This study	NCBI: OP959806
VK51 - isolate 16S amplicon sequence	This study	NCBI: OP967914
**Experimental models: Organisms/strains**		
Sorghum bicolor var. Shanqui Red	GRIN	PI 656025
Sorghum bicolor var. SRN39	Ethiopian Institute of Agricultural Research	PI 656027
Striga hermonthica	Abdelgabar Babiker	N/A
**Oligonucleotides**		
16S_V3-341F	CCTACGGGNGGCWGCAG	N/A
16S_V4-785R	GACTACHVGGGTATCTAATCC	N/A
ITS3_F	GCATCGATGAAGAACGCAGC	N/A
ITS4_R	TCCTCCGCTTATTGATATGC	N/A
**Software and algorithms**		
ImageJ	NIH, USA	https://ImageJ.nih.gov/ij
MassLynx™ version 4.1	Waters™	https://www.waters.com/waters/en_US/MassLynx-Mass-Spectrometry-Software/nav.htm?locale=-&cid=513662

(Continued on next page)

Continued

REAGENT or RESOURCE	SOURCE	IDENTIFIER
BioTransformer 3.0	Djoumbou-Feunang et al.[25]	http://biotransformer.ca
DIRT version 1.1.	Das et al.[57]	N/A
FastQC	Babraham Bioinformatics	https://www.bioinformatics.babraham.ac.uk/projects/fastqc/
fastx-trimmer	FASTX-toolkit	http://hannonlab.cshl.edu/fastx_toolkit/index.html
STAR	Dobin et al.[64]	N/A
R edgeR package	Robinson et al.[65]	N/A
R Limma package	Ritchie et al.[66]	N/A
UPARSE	Edgar[70]	N/A
R phyloseq package v.1.26.1	McMurdie and Holmes[74]	N/A
R gjam package version2.6.2	Clark et al.[75]	N/A

RESOURCE AVAILABILITY

Lead contact

Further information and requests for resources and reagents should be directed to and will be fulfilled by the lead contact, Siobhan Brady (sbrady@ucdavis.edu).

Materials availability

Plant material and bacterial isolates will be available upon request and may require a completed materials transfer agreement, import permits and phytosanitary certificates.

Data and code availability

- Sorghum transcriptome sequences were deposited in NCBI GEO under the accession number GSE 216351. Amplicon sequences from microbiome profiling are available on ENA under the accession number PRJEB57848. Amplicon sequences of tested isolates are available in the NCBI under the following accession numbers: VK6: KX503329, VK14: KX503337, VK15: KX503338, VK46: KX503369, VK48: OP954904, VK49: OP959794, VK50: OP959806, VK51: OP967914.
- Raw data from the growth measurements, root system architecture analysis, ANOVA tables and p values for each statistical test can be found in Data S1. CPM values and lists of differentially expressed genes are presented in Data S2. Residual correlations from GJAM and ranks assigned to each microbial taxa are to be found in Data S3 and Data S4, respectively. Residual correlations and rank for the members of the microbial collection are presented in Data S5. An overview of bacterial taxa found at two weeks post-infection in (A) bulk soil, (B) rhizosphere, (C) soil plug-associated roots and three weeks post-infection in (D) bulk soil, (E) rhizosphere, (F) soil plug-associated roots, (G) sand-associated roots, predicted to influence Striga infection via each of identified mechanisms is presented in Data S6. Raw data and results of statistical analysis from the experiments with individual bacterial isolates can be found in Data S7.
- Data analysis scripts are publicly available at https://github.com/DorotaKawa/Striga-suppressive-soil. The script for generalized joint attribute modeling can be found at https://github.com/Leitemfa/GJAM-PROMISE.
- Any additional information required to reanalyze the data reported in this paper is available from the lead contact upon request.

EXPERIMENTAL MODEL AND STUDY PARTICIPANT DETAILS

Soil material

Soil was collected from the Clue Field in the Netherlands; 52° 03′ 37.91″ N and 5° 45′ 7.074″ E.[22] Soil was dried and sieved through a 4 mm mesh and one batch of it was sterilized by gamma irradiation with a dose of 8kGy, at room temperature by Steris (the Netherlands). Description of the physiochemical properties of "natural" and "sterilized" soils were provided by Eurofins Agro (the Netherlands, Data S1).

Plant material and growth conditions – soil "plug" assay

Seed of *Striga hermonthica* were collected in Sudan and kindly donated by Abdelgabar Babiker, Seeds were sieved by a mesh of 200 ʃm pores to remove remaining soil particles and flower debris. Seeds were then surface sterilized with 10% (v/v) bleach and 0.02% (v/v) Tween 20 on filter paper and placed on a Buchner funnel connected to a vacuum pump until all liquid was removed. Next, seeds were washed twice for 5 min in sterile water. Sterilized seeds were left to dry on the filter paper overnight in a laminar flow hood. Sterile

CellPress
OPEN ACCESS

seeds were mixed with sand containing around 16% (w/v) water and pre-conditioned for 10 days in a dark container in the greenhouse with temperature set to 26°C. As a negative control, sand without Striga seeds was treated in the same manner.

Seeds of *Sorghum bicolor* var. Shanqui Red (SQR) were obtained from GRIN (https://www.ars-grin.gov) and SRN39 seeds were kindly donated by the Ethiopian Institute of Agricultural Research. Seeds were surface sterilized by agitating in a solution containing 4% (v/v) sodium hypochlorite and 0.2% (v/v) Tween 20 for 45 min followed by three rounds of 30 s incubations in 70% (v/v) ethanol followed by washes with sterile water. Next, seeds were washed four times with sterile water. Sterilized seeds were germinated on a wet Whatman paper (grade 1) at 28°C for 48 h in the dark, followed by 48 h in light. Four-day-old seedlings with approximately the same radicle length were transferred to 50 mL tubes filled with "natural" or "sterilized" soil (referred hereafter as the soil plug) mixed with 5% sterile water (w/v). Seedlings were watered with sterile water every second day. After 10 days, seedlings together with the soil plug were transferred to 40 cm long cones (Greenhouse Megastore, USA, catalog number CN-SS-DP) that were autoclaved prior to transfer. The bottom layer of the cones was filled with 350 mL of filter sand (0.5–1.0 mm, filcom.nl/) and the upper layer was filled with 350 mL preconditioned sand without (control) or with Striga seeds (3000 germinable Striga seeds per cone). Plants were organized in a randomized manner in the greenhouse compartment with the temperature set to 28°C during the day (11 h) and 25 °C at night (13 h) with the 70% relative humidity and light intensity of 450 ⌠mol/m^2/s. All measurements and sample collections were carried out at 14 and 21 days upon transfer to cones (referred to as two- or three-weeks post-infection; wpi). At day zero, seven and 14 (where day 0 is the day of the transfer to cones) plants were watered with 50 mL modified half-strength Hoagland solution containing 0.05 mM KH_2PO_4. On days one, four, 10, 13 and 17, plants were watered with 50 mL deionized sterile water.

METHOD DETAILS

Striga infection quantification

Six individual plants of SQR were used per treatment (Striga-infected and control) at each time point (2,3 wpi). SRN39 was used as a negative control for Striga infection, and no Striga attachments were observed on SRN39 roots at any conditions (Figure S1E). Sorghum plants were gently removed from the cones. The remaining sand and soil plug from the cone was collected and carefully examined for detached Striga plants. Roots were then gently washed in water and inspected under a dissecting microscope for early stages of Striga attachment. Roots were dried with a paper towel and fresh weight was recorded. The infection level was expressed as the ratio of total Striga attachments (the sum of early Striga attachments and the number of Striga plants recovered from the sand) and fresh root weight of individual plants. Data were analyzed with a two-way ANOVA, where a linear model was specified as: trait value = Soil+Treatment+Soil:Treatment, where Treatment stands for Striga infection or its absence (control).

Exudate collection and profiling

Each cone was flushed with water to collect 1 L of the flow-through. 100 mL of exudate were purified using solid phase extraction (SPE) with C18 Discovery cartridges (bed wt. 500 mg, volume 6 mL, Merck). Cartridges were activated using 5 mL acetone and washed with 5 mL distilled water. One hundred mL of sample was loaded on the cartridge and the flow through was collected. The cartridge was further washed with 6 mL distilled water. Finally, compounds were eluted using 3 mL acetone. The acetone was evaporated using a SpeedVac (Scanvac, Labgene, Châtel-Saint-Denis, Switzerland). The semi-polar fraction of the exudates was reconstituted in 150 μL 25% (v/v) acetonitrile and filtered using a micropore filter (0.22 μm, 0.75 mL, Thermo scientific). The collected flow-through was freeze dried (Heto Powerdry LL1500, Thermo) and extracted with absolute methanol to remove the salts. The methanol was subsequently evaporated using a SpeedVac (Scanvac, Labgene, Châtel-Saint-Denis, Switzerland). The polar fraction of the exudates was reconstituted in 150 μL 25% (v/v) acetonitrile and filtered using a micropore filter (0.22 μm, 0.75 mL, Thermo Scientific).

Untargeted analysis was performed as described in Kawa et al., 2021.[23] Briefly, 5 μL of root exudates (semi-polar and polar fraction) were injected on a Nexera UHPLC system (Shimadzu, Den Bosch, The Netherlands) coupled to a high-resolution quadrupole time-of-flight mass spectrometer (Q-TOF; maXis 4G, Bruker 194 Daltonics, Bruynvisweg 16/18). Compounds were separated on a C18 stationary phase column. Peak finding, peak integration and retention time correction were performed as in Kawa et al., 2021.[23]

Targeted phenolic analysis was performed on a Waters Acquity UPLC I-Class System (Waters, Milford, MA, USA) equipped with Binary solvent manager and Sample manager was employed as a chromatographic system coupled to a Xevo TQ-S tandem quadrupole mass spectrometer (Waters MS Technologies, Manchester, UK) with electrospray (ESI) ionization interface. Five μL of root exudates (semi-polar and polar fraction) were separated on an Acquity UPLC BEH C18 column (2.1 × 100 mm, 1.7 μm particle size, Waters, Milford, MA, USA) with 15 mM formic acid in both water (A) and acetonitrile (B). At a flow rate of 300 μL per min and a column temperature of 40°C, the following gradient was applied: 0 min, 5% B; 2 min, 5% B; 32 min, 18% B; 60 min, 24% B; 65 min, 100% B. The compounds were measured in the ESI ion source of the tandem mass analyzer operating in the same conditions as in.[56] Mass data of phenolic compounds were acquired in multiple reaction monitoring (MRM) mode. The MassLynx software, version 4.1 (Waters), was used to control the instrument as well as to acquire and process MS data.

Prediction of microbial degradation products

The structures of five HIFs: DMBQ, syringic acid, vanillic acid, vanillin, acetosyringone were input in the web-based tool BioTransformer to predict their microbial degradation products (http://biotransformer.ca/).[25] The first level predicted conversion compounds were used as an input for secondary conversion products. The exact masses of the degradation products were matched

with the untargeted profiles of root exudates to retrieve potential candidates within a range of 25ppm error. Abundances of tested compounds in root exudates of plants grown in “natural” and “sterilized” soil (soil “plug” system) in the absence of Striga were compared with a Student’s t-test with a false discovery rate adjustment from multiple comparisons. The predicted conversion products of m/z larger than HIFs used as an input for the BioTransformer that were detected likely due to the presence of HIF active compounds with larger mass or is due to non-causal correlation with larger, non-HIF, metabolites that are degraded by microbes, or are products of microbial biosynthesis.

In vitro germination and haustorium formation assay

Two hundred mg of Striga seeds were surface sterilized in 2% sodium hypochlorite containing 0.02% Tween 20 for 5 min, and then washed 5 times with sterile MilliQ water. The sterilized Striga seeds were spread on sterile glass fiber filter papers (Whatman GF/A, Sigma-Aldrich) in petri dishes moistened with 3 mL sterile MilliQ and preconditioned for 6–8 days at 30°C. 0.1 ppm racGR24 (racemic mix of enantiomers $GR24^{5DS}$ and $GR24^{ent-5DS}$) and 100 mM DMBQ was used as positive control for Striga germination and haustorium formation, respectively. Stock of GR24rac was prepared in acetone and DMBQ was dissolved in methanol/water 50% (v/v). Dried, preconditioned Striga seeds were treated with 300 times-diluted root exudates from plants grown in the “natural” or “sterilized” soil, or GR24rac or DMBQ, and each of the solutions was further divided into 3 technical replicates. Striga seeds were incubated in the dark at 30°C for 2 days, when the number of germinated Striga and haustoria formed were counted. The Striga germination rate was calculated for each replicate using the formula: GR% = (Ngs/Nts) × 100, where Ngs is the number of germinated seeds per well and Nts is the total number of seeds per well. Haustorium formation was assessed by counting the number of germinated seeds that developed early stage haustoria (pre-haustoria). The haustorium formation rate (HFR%) was calculated for each replicate using the formula: HFR % = (NHs/Ngs) × 100, where NHs is the total number of haustorium per well and Ngs is the number of germinated seeds per well. Welch t-sample test was used to compare effects elicited by the exudates of plants grown in the “natural” and “sterilized” soil.

Root system architecture phenotyping

Data was collected 2 and 3 weeks post-infection (wpi), when plants were 4 and 5-weeks-old, respectively. Plant height was scored as the length from the sand surface to the bend of the highest leaf. Sorghum plants were removed from the cones and roots were cleaned from the sand and soil plug by gentle washes in water. Crown roots were separated from seminal roots and their fresh weight was separately scored. Roots were then placed in a transparent tray filled with water and scanned at 800dpi resolution with an Epson Perfection V700 scanner. Next, roots were dried with a paper towel, placed in paper bags, dried for 48 h in 65°C and weighed to determine their dry weight.

Root system architecture was analyzed with the DIRT (Digital Imaging of Root Traits) software v1.1.[57] The total root network area and total network length (to simplify we refer to it as total root area and total root length) used skeleton methods[58,59] as described in Kawa et al.[23] Mean root network diameter was calculated as the ratio of network area over network length. The dataset was cleaned from extreme outliers by removing individuals with values outside the 3rd quartile. All collected data were analyzed with a two-way ANOVA, where a linear model was specified as: trait value = Soil+Treatment+Soil:Treatment, where Treatment stands for infected or not infected (control) with Striga. The root system architecture data of SQR plants from sterilized soil in the absence of Striga were previously published in Kawa et al.[23]

Root cellular anatomy phenotyping

Sorghum plants (2 and 3 wpi) were gently taken from the cones and washed in water to remove remaining sand and soil. For each plant a 1.5 cm segment of root tissue was cut from the tip of a crown root, from the middle of a crown root and from the middle of a seminal root. For comparison of SQR root cellular anatomy at the seedling stage, sterilized seeds were placed in 25 cm long germination pouches (PhytoAb Inc., catalog number: CYG-38LG) filled with 50 mL of autoclaved water. Root tissue was harvested from 10-day-old seedlings. For each plant a segment of root tissue was cut from 7 cm distance from a root tip.

Root tissue was embedded in 5% (w/v) agar and fixed by a 10 min vacuum infiltration in FAA solution (50% ethanol 95%, 5% glacial acetic acid, 10% formalin, 35% water, all v/v) followed by overnight incubation in FAA and rehydration by 30 min incubations in a sequence of 70%, 50%, 30% and 10% (v/v) ethanol. Embedded tissue was stored in water at 4°C. Sections of 200–300 μm thickness were made with a Leica VT1000 vibrating microtome.

Suberin was stained with 0.01% (w/v) Fluorol Yellow 088 in lactic acid at room temperature, in the dark, for 30 min. Sections were rinsed three times for 5 min with water. Counter staining was done with 0.5% (w/v) aniline blue at room temperature for 30 min, followed by four 10-min washes with water. Sections were mounted on slides with 50% glycerol prior to microscopic examination. Sections were imaged with an LSM 700 laser scanning microscope (Carl Zeiss) with an excitation wavelength 488 nm and gain-optimized to the signal strength of the sample with the highest fluorol yellow signal. All images were taken with the same settings. Quantification of endodermal suberin was done by calculating the mean fluorescence of two representative endodermal cells per section in ImageJ. The mean for two cells per section was used for further analysis.

For the aerenchyma quantification, cross-sections were prepared from the exact same region of the exact same root as for suberin visualization and were stained for 5 min in 0.1% toluidine blue (w/v) followed by five brief washes with water. Brightfield images were taken with an Olympus AH-2. The proportion of aerenchyma was expressed as the percentage of the area of the root section. The

number of cortex layers and the number of metaxylem vessels were scored manually. The data collected from the cones experiment were analyzed with a two-way ANOVA. For each genotype-time point data subsets a linear model was specified as: trait value = Soil+Treatment+Soil:Treatment, where Treatment stands for infected or a not infected (control) with Striga. The data from the experiment with seedlings were derived from two independent experiments, thus a mixed model was used with experimental batch (Exp) as an independent factor specified with the formula: lmer (trait ∼Genotype + (1|Exp)) with lme4 v.1.1–21 R package.

RNA-seq library preparation

Two and three weeks after Striga infection (corresponding to 4- and 5-week-old plants), root material was harvested 2 h after the light turned on. Each sorghum plant was gently removed from the cone and whole root system was cleaned from the remaining sand and soil by washing in water, dried with paper towel and snap frozen in liquid nitrogen (whole process took approximately 3 min per plant). Root tissue was ground with pestle in mortar, and RNA was extracted with RNaesy Plus Mini kit (Qiagen) with application of cell lysate on the QIAshredder columns (Qiagen) followed by the on-column Dnase I (Qiagen) treatment. Extracted RNA was precipitated with 3M NaOAc pH 5.2 (Thermo Scientific) in 100% ethanol and the pellet was washed with 70% (v/v) ethanol and dissolved in RNase-fee water. RNA-seq libraries were prepared with QuantSeq 3′ mRNA-Seq Library Prep Kit (Lexogen) following manufacturer protocol. Four biological replicates and three technical replicates for each RNA sample were used.

Libraries were sequenced at the UC Davis DNA Technologies Core with Illumina HiSeq 4000 in SR100 mode.

Microbial community analysis

The "natural" and "sterilized" soil plugs were prepared and placed in cones filled with sand as described above, except no plant was transferred. The soil plugs and cones were placed in the same greenhouse compartment as cones with plants and were watered according to the same scheme. Fourteen days after transfer to cones, the soil plug was excavated from the sand for the DNA extraction. These samples were used to profile the microbiome communities of the bulk soil in the absence of the plant (Data shown in Figures 1A and 1B).

The microbiome communities in the bulk soil in the presence of a plant and those associated with sorghum roots, bulk soil, rhizosphere and root material were collected 14 and 21 days after transfer to cones as in[60] with small modifications. First, soil not associated with roots was collected, shaken for 30 s in 35 mL sterile phosphate buffer, centrifuged at 3,000 rpm for 20 min. Collected pellets were frozen in liquid nitrogen and constituted "bulk soil" samples. Roots that grew in the soil plug were carefully separated from those that grew out of the soil plug and into the sand compartment. The excess of soil and sand was gently removed to leave a thin layer of 1–2 mm on the root surface. The roots were then shaken in 35 mL sterile phosphate buffer and transferred to a sterile Petri dish containing phosphate buffer to be thoroughly washed and to remove remaining soil/sand particles. The phosphate buffer was centrifuged at 3,000 rpm for 20 min, soil and sand pellets were frozen in liquid nitrogen and constitute a "rhizosphere soil" or "root-associated sand" samples. The fresh weight of washed roots was scored, and roots were immediately frozen in liquid nitrogen (and constituted "soil plug-associated roots" and "sand-associated roots" sub-categories).

DNA was extracted with MoBio PowerSoil DNA isolation kit (Qiagen, Germany) from approximately 300 mg of ground root material or 250 mg of soil/sand as recommended by the manufacturer. Prior to extraction from sand and soil, an additional centrifugation step was performed (10000 rpm at 4°C for 5 min). DNA concentrations were measured with a Nanodrop 2000 spectrophotometer (Thermo Fisher Scientific, USA) and stored at −80°C for further analysis. The DNA yield from "root-associated sand" was not sufficient for sequencing, thus these samples were discarded from further analysis.

Microbial communities were characterized by sequencing amplicons of the 16S rRNA region V3-V4 (with primer set: 16S_V3-341F: CCTACGGGNGGCWGCAG, 16S_V4-785R: GACTACHVGGGTATCTAATCC) for the bacteria, and ITS3-ITS4 (with primer set: ITS3_F: GCATCGATGAAGAACGCAGC, ITS4_R: TCCTCCGCTTATTGATATGC) for fungi. The amplicons were sequenced with Illumina MiSeq by BaseClear (Leiden, Netherlands).

Haustorium formation and HIF quantification with individual bacterial isolates

A single bacterial colony was selected to inoculate minimal media (0.5% NaCl, 0.1% KH_2PO_4, 0.01% BactoTM Yeast-Extract, pH = 5.0) supplemented with 2mM N-acetylglucosamine. The cultures were grown for 24 h at 25°C with shaking (200 rpm). Overnight cultures were spun down at 8000 rpm for 5 min, and the collected cells were washed twice with 10mL sterile 0.9% NaCl. Cells were suspended in 0.9% NaCl and OD600 was adjusted to 0.15. Eighteen μL of bacterial suspension was added to 332 μL of the N-acetyloglucosamine media supplemented with 100 μM of syringic acid or 50 μM vanillic acid dissolved in methanol or an equivalent volume of methanol as a negative control (four biological replicates per isolate). After growth for another 24 h, 50 μL of the cell-free culture filtrate was applied to Striga seeds pre-germinated with 100 μL 1μM GR24. Striga seeds were surface sterilized with 0.5% sodium hypochlorite for 5 min, rinsed three times with sterile water and preconditioned at 28°C for 12 days in dark on a wet 13 mm disk GF/A filter paper (VWR, Whatman). After 48 h of Striga seed incubation with the cell-free culture filtrate at 28°C, Striga seeds were imaged. The percentage of seeds that developed haustorium from all germinated seeds was quantified with the ImageJ Cell Counter plug-in. For the experiment with multiple isolates, four replicates (an individual replicate consisted of 100 seeds on one filter paper disk) were used per treatment. For the second experiment, including only isolate VK46, four individual cell-free culture filtrates were used, each applied on four disks with Striga seeds. Prior to centrifugation, an aliquot of the liquid culture was used to measure the OD, to ensure an equal rate of bacterial growth in all treatments. Forty μL of each filtrate was collected at the time

of application to Striga seeds ($t_{0,}$ to ensure no HIF degradation during the incubation) and at the time of haustoria quantification t_{24}) and concentrations of vanillic and syringic acid were quantified with LC-MS as described for the targeted analysis in the section exudate collection and profiling. The mock treatment was methanol. Statistical analyses were performed with a one-way ANOVA with a Tukey post-hoc test.

Sorghum inoculation with individual bacterial isolates

Individual isolates were cultured in a 1/10 dilution of tryptic soy broth (TSB, Difco) agar (1.5%, m/v) media containing 50 mg/L thiabendazole (Sigma) and incubated for 48 h at 26°C. A single colony was then used to inoculate liquid TSB media (1/10 media dilution) and incubated for 48 h at 26°C with shaking (200 rpm). Bacteria were harvested by centrifugation at 5000 rpm for 20 min and the resulting pellet was resuspended in sterile modified half-strength Hoagland solution (see methods for soil "plug" assay). In all the experiments individual isolates were applied to the sorghum variety Shanqui Red (SQR). An individual plant was inoculated with 10^7 CFU/g sand in 5 mL of half-strength Hoagland media. The inoculum was applied to the root of a two-day-old sorghum seedling (pregerminated on wet Whatman paper for 48 h at 28°C) at the same time as seedling transplantation into a 50 mL tube filled with sand (moistened with 5 mL half-strength Hoagland media beforehand). Plants were watered every second day with 5 mL sterile water.

Aerenchyma quantification in the presence of individual isolates

To estimate the proportion of aerenchyma, we measured the porosity of the entire root system two weeks post inoculation following the protocol of.[61] Roots were gently removed from the sand, washed in water, very gently dried with a paper towel and weighed in 25 mL pycnometers (Eisco Labs) were filled with water and weighted. The harvested root systems were placed in an individual pycnometer, refilled with water and weighed. Next, the pycnometers with roots were subjected to vacuum infiltration until the last air bubbles were seen, and their weight was scored. Root system porosity was calculated as: porosity = $(P_v - P_r)/(P_w + R\text{-}P_r)$ where P_w is the weight of the pycnometer filled with water; P_r is the weight of the pycnometer filled with water and containing the root system; P_v is the weight of the pycnometer with a vacuum infiltrated root system and R is the root system weight at the moment of harvest. All tested isolates were first screened in two separate experiments with n = 6. Next, the isolates with the largest difference from the mock treatment were tested again with higher replication (n = 15).

Suberin quantification -- individual isolates assay

One week after bacterial inoculation, roots were gently collected from the sand, washed in water and 1–1.5 cm of root segments were cut from two regions: 3–4 and 6-7cm from the root tip of the primary root. Region 3–4 cm constitutes the "patchy" suberization zone in the mock-treated SQR root, while 6-7cm is the zone where the first onset of exodermis suberization is usually seen. Root tissue was embedded in agar, fixed in FAA, sectioned, and stained with fluorol yellow and imaged as described in the root cellular anatomy phenotyping section. To quantify the differences along the root's longitudinal axis, we also quantified the proportion of suberized and non-suberized cells in the endodermis. Given the technical challenges with obtaining sections that can be visualized in one plane, we excluded the areas of sections that were not completely perpendicular to the root's longitudinal axis. These regions were determined by following the changes in the background fluorescent signal from the vasculature. The regions with less fluorescence in the vasculature, and adjacent endodermal cells were excluded from the analysis and are depicted in Figure S8. We also determined the presence and absence of the suberized exodermis. Statistical analyses were done with a generalized linear model, for the proportion of plants with a fully suberized endodermis: glm(Fully_suberized ~ Strain, family = binomial(link = "logit")), for the proportion of suberized cells: glm(cbind('Number of Suberized','Number of Non Suberized') ~ Strain, family = quasibinomial(link = "logit")), followed by comparison of each isolate with mock treatment with emmeans with option type = "response" with emmeans R package 1.8.1–1. The proportion of plants with a suberized exodermis was tested with Fisher's exact test between each isolate and mock. Sections from 10 to 15 individual plants per treatment were used.

QUANTIFICATION AND STATISTICAL ANALYSIS

RNA-seq read processing and differential expression analysis

Quality control of obtained transcriptome sequences was performed with FastQC (http://www.bioinformatics.babraham.ac.uk/projects/fastqc/) before and after read processing. Three technical replicates of each library were pooled before read processing. Barcodes were removed from raw reads with fastx-trimmer (http://hannonlab.cshl.edu/fastx_toolkit/index.html) with parameters: -v -f 12 -Q33. A wrapper from Kraken Suite[62] was used for adaptor trimming and quality filtering with options: -geom no-bc -tabu $tabu -3pa $seqAdapt -noqc -dust-suffix 6/ACTG -dust-suffix-late 6/ACTG -nnn-check 1/1 -qqq-check 35/10 -clean-length 30 -polya 5. Processed reads were mapped to the reference genome of *Sorghum bicolor* BTx623[63] using STAR[64] with options: –outFilterMultimapNmax 20 –alignSJoverhangMin 8 –alignIntronMin 20 –alignIntronMax 10000 –outFilterMismatchNmax 5 –outSAMtype BAM SortedByCoordinate –quantMode TranscriptomeSAM GeneCounts.

Genes for which no raw reads were detected across all samples were removed. Counts per million (CPM) were calculated with the cpm() function from the edgeR package.[65] Only genes with a CPM >1 in at least three samples were used for further analysis. CPM values are listed in Data S1 and Dataset S2. Differentially expressed genes (DEGs) were determined with the R/Bioconductor limma package.[66] CPM values were normalized with the voomWithQualityWeights() function with quantile normalization to account for

different RNA inputs and library sizes. Data from 4- and 5-week-old plants were analyzed separately. For each gene the linear model was defined as an interaction of the soil type ("natural" or "sterilized") and treatment (control or infected with Striga) as: log(counts per million) of an individual gene ~ Soil*Treatment. Differentially expressed genes for each term of the linear model were selected based on a false discovery rate of <0.05. Lists of differentially expressed genes for each term (soil, treatment, soil by treatment) are found in Data S1 and Dataset S2.

Gene orthology identification

A list of sorghum orthologs of Arabidopsis suberin biosynthetic genes was obtained from.[28] To identify sorghum orthologs of ABCG transporter family proteins, a phylogenetic tree was generated as described in.[32] Next, we created a list of 672 maize genes whose expression was shown to change during root aerenchyma formation as reported in.[67–69] Sorghum orthologs of maize genes were obtained from www.maizegdb.org. In total 447 unique sorghum genes have been defined as orthologs of maize genes associated with aerenchyma formation Data S1 and Dataset S2. Enrichment of these genes among genes differentially expressed by soil type (2 wpi) was tested with Fisher's Exact test.

Microbial community analysis

The 16S rRNA region V3-V4 amplicons were sequenced with Illumina MiSeq by BaseClear (Leiden, Netherlands). Raw sequence processing and quality control were performed with the UPARSE pipeline.[70] In brief, reads were paired and trimmed for quality (maximal expected errors of 0.25, reads length >250 bp). Sequences were clustered into Operational Taxonomic Units (OTUs) at 97% of nucleotide identity, followed by chimera removal using UCHIME.[71] Taxonomic assignments of representative OTUs were obtained using the RDP classifier[72] against the Silva Database.[73] Sequences affiliated with chloroplasts were removed.

Analysis of microbial communities of "natural" and "sterilized" bulk soil without sorghum planted was performed with R phyloseq package v.1.26.1.[74] Data was transformed with RLE normalization and rescaled to median sample count. Alpha diversity of each sample was calculated with estimate_richness function with "measures" set to "Shannon". Significance of the difference between bulk "sterilized" and "natural" soil was determined with a Student's t-test.

Identification of microbial candidates associated with reduced Striga infection

Statistical analyses were conducted in R v4.0.1 using different packages. To identify microbial taxonomic units associated with reduced Striga infection via the identified host traits, generalized joint attribute modeling was used (gjam package version2.6.2[75]). This modeling estimates the effects of soil sterilization and Striga infection on microbial communities (bacteria and fungi) within individual microbiome sub-categories (bulk soil, rhizosphere, soil plug-associated roots and sand-associated roots) and the number of Striga attachments and traits associated with Striga suppression (aerenchyma content, endodermal suberization, abundances of syringic acid and vanillic acid). The model analysis returns regression coefficients from the effect of the different treatments and quantified the increase or decrease in the microbial relative abundance and the changes in the other variables. Model diagnosis evaluated using Markov Chain Monte Carlo (MCMC) to check when the estimated coefficients reached a stable value (after 10,000 simulations). Since the experiment consisted of a two-way factorial design, regression coefficients were compared against the following hypotheses: H1 - within each soil type ("natural" or "sterilized") there is a difference between the Striga treatments (infected vs. control); H2 - within each Striga treatments there is a difference between soil types. The model was applied for individual traits for each time point, at which they were found to be affected by the soil type, but not Striga infection, thus for 2 wpi: Striga attachments, aerenchyma content, abundances of: syringic acid, vanillic acid and; for 3 wpi: Striga attachments, aerenchyma content, endodermal suberization).

As a joint model, gjam also allows the extraction of residual correlations to investigate the relationship between the soil microbiome, Striga infection and associated traits.[76] The residual correlations measure how strongly two different variables are associated regardless of the influence of the treatment, which is therefore used to seek potential biotic interactions.[77] In our study, residual correlations were calculated per each microbial sub-categories and are listed in Data S3.

Residual correlations calculated for individual microbial sub-category for each trait were filtered as follows: negative correlations for the number of Striga attachments and HIF abundance (vanillic acid, syringic acid), while positive correlations were kept for suberin content and aerenchyma proportion for further analysis. We first ranked the taxa based on their correlation for each trait across microbial sub-categories. Then, to identify taxa potentially reducing Striga attachment number via each of identified mechanisms, ranking was done for each sub-category separately. Ranks for Striga attachment number and HIF levels were assigned so that the taxa with the lowest correlation received the highest rank value. Ranks for aerenchyma proportion and suberin content were assigned so that the taxa with the highest correlation received the highest rank value. Taxonomic membership was summarized for the top 100 bacterial taxa. The difference in the number of taxa that were summarized is due to less fungal taxa present in the Clue Field soil as compared to bacterial taxa.

Next a sum of ranks for Striga attachments number with a rank for each of: vanillic acid, syringic acid, suberin content, aerenchyma proportion, was calculated. The combined ranking was calculated as the rank of this sum. Individual and listed ranks calculated per each microbial sub-category are presented in Data S4. Taxonomic membership was summarized with a cut-off of residual correlation −0.2 for Striga attachments, syringic acid and vanillic acid levels and 0.2 for aerenchyma proportion and suberin content.

From the collection of bacterial strains isolated from the Clue Field soil by,[31] we selected isolates belonging to genera whose residual correlation was higher than 0.2 for suberin content and aerenchyma proportion and lower than −0.2 for Striga attachment

number and HIFs abundance. By this we selected as candidates for reduction of Striga suppression via: i) promotion of aerenchyma formation: *Arthrobacter*, *Aeromicrobium*, *Bradyrizobium*, *Nocardia*, *Mesorhizobium*, *Paenibacillus*, *Phenylobacterium*, *Pseudomonas*; ii) degradation of vanillic and syringic acid: *Arthrobacter*, *Aeromicrobium*, *Pseudomonas* ii) induction of suberin deposition: *Arthrobacter*, *Aeromicrobium*, *Bradyrizobium*, *Nocardia*, *Mesorhizobium*, *Paenibacillus*. From the isolates we were able to re-grow and confirm their taxonomic identity by re-sequencing 16S rRNA (with primer set FQ 5′-AGAGTTTGATCCTGGCTCAG-3′ and REV 5′-GGTTACCTTGTTACGACTT-3′), four Pseudomonas, and four Arthrobacter isolates were used for *in vitro* tests of HIF degradation and inoculation of sorghum plants. Residual correlations derived from the generalized joint attribute modeling and ranks calculated for each genera in the collection can be found in Data S5.

Cell Reports, Volume *43*

Supplemental information

The soil microbiome modulates the sorghum root metabolome and cellular traits with a concomitant reduction of Striga infection

Dorota Kawa, Benjamin Thiombiano, Mahdere Z. Shimels, Tamera Taylor, Aimee Walmsley, Hannah E. Vahldick, Dominika Rybka, Marcio F.A. Leite, Zayan Musa, Alexander Bucksch, Francisco Dini-Andreote, Mario Schilder, Alexander J. Chen, Jiregna Daksa, Desalegn W. Etalo, Taye Tessema, Eiko E. Kuramae, Jos M. Raaijmakers, Harro Bouwmeester, and Siobhan M. Brady

Supplementary Figures

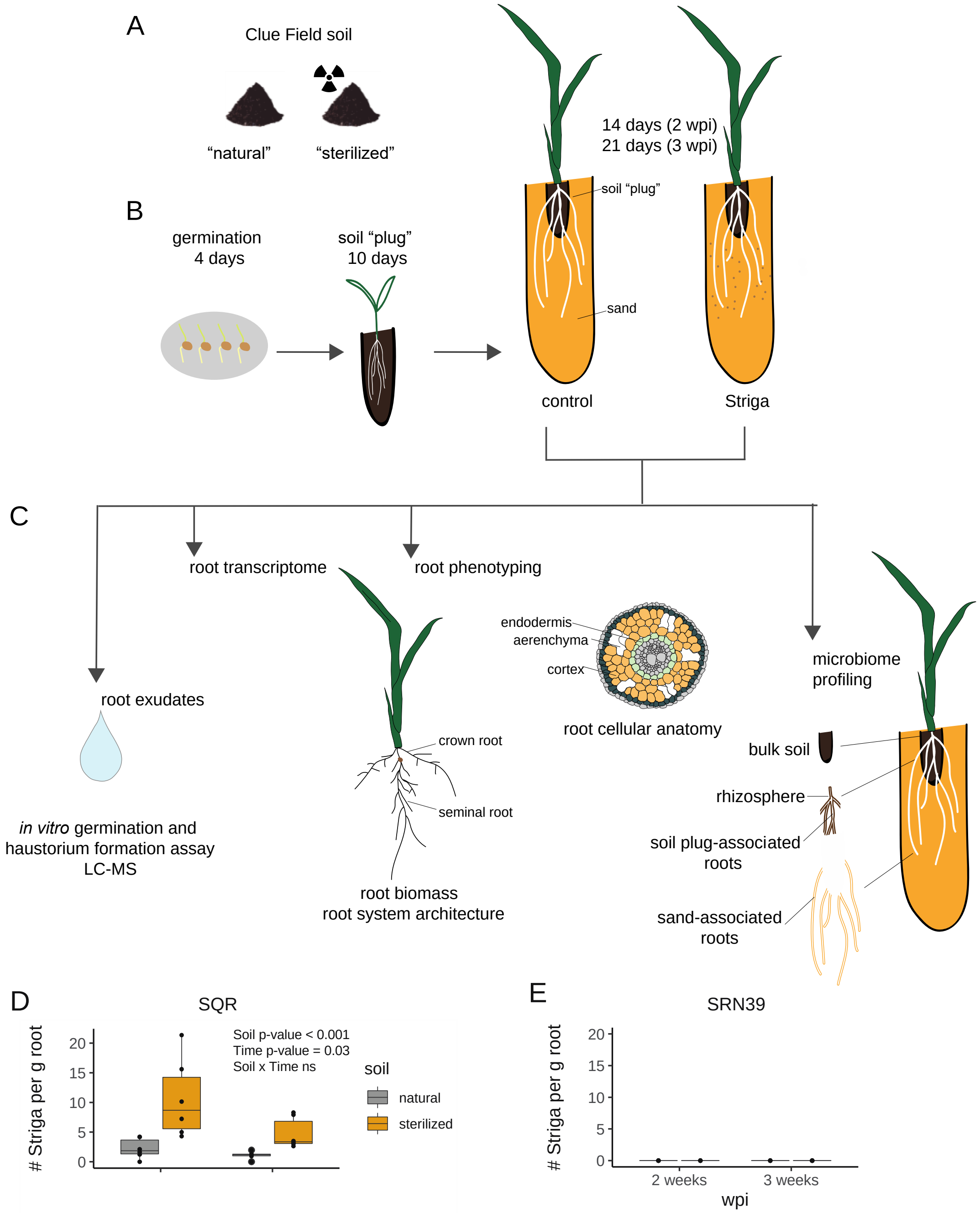

Supplementary Figure 1. Visual description of the methodology used to investigate the mechanisms of Striga infection suppression by soil microbiome. (A) Two batches of Clue Field soil were tested: “natural” and “sterilized”, the latter subjected to gamma-irradiation. (B) Sorghum seedlings were germinated and grown for 4 days on moistened filter paper and then (B) transferred to a soil “plug” for 10 days. The seedling, together with the soil “plug” was then transferred to conical tubes filled with sand (control) or sand mixed with Striga seeds. (C) Root tissue and root exudates were collected two and three weeks post-infection (wpi). The root exudates were used for the *in vitro* germination and haustorium formation assay and metabolite analyses. Root phenotyping included quantification of root system architecture and cellular anatomy. Root transcriptomes were profiled with RNAseq. Microbiome profiles were obtained from bulk soil, rhizosphere, soil plug-associated roots (root system part in contact with soil “plug”) and sand-associated roots (the root system that was in contact with sand). (D) Number of Striga attachments per gram of fresh root weight of Striga susceptible variety Shanqui Red (SQR) and (E) Striga resistant SRN39 at two and three weeks post-infection (wpi) in natural and sterilized soil. Significance of the differences was assessed with a two-way ANOVA (n=6).

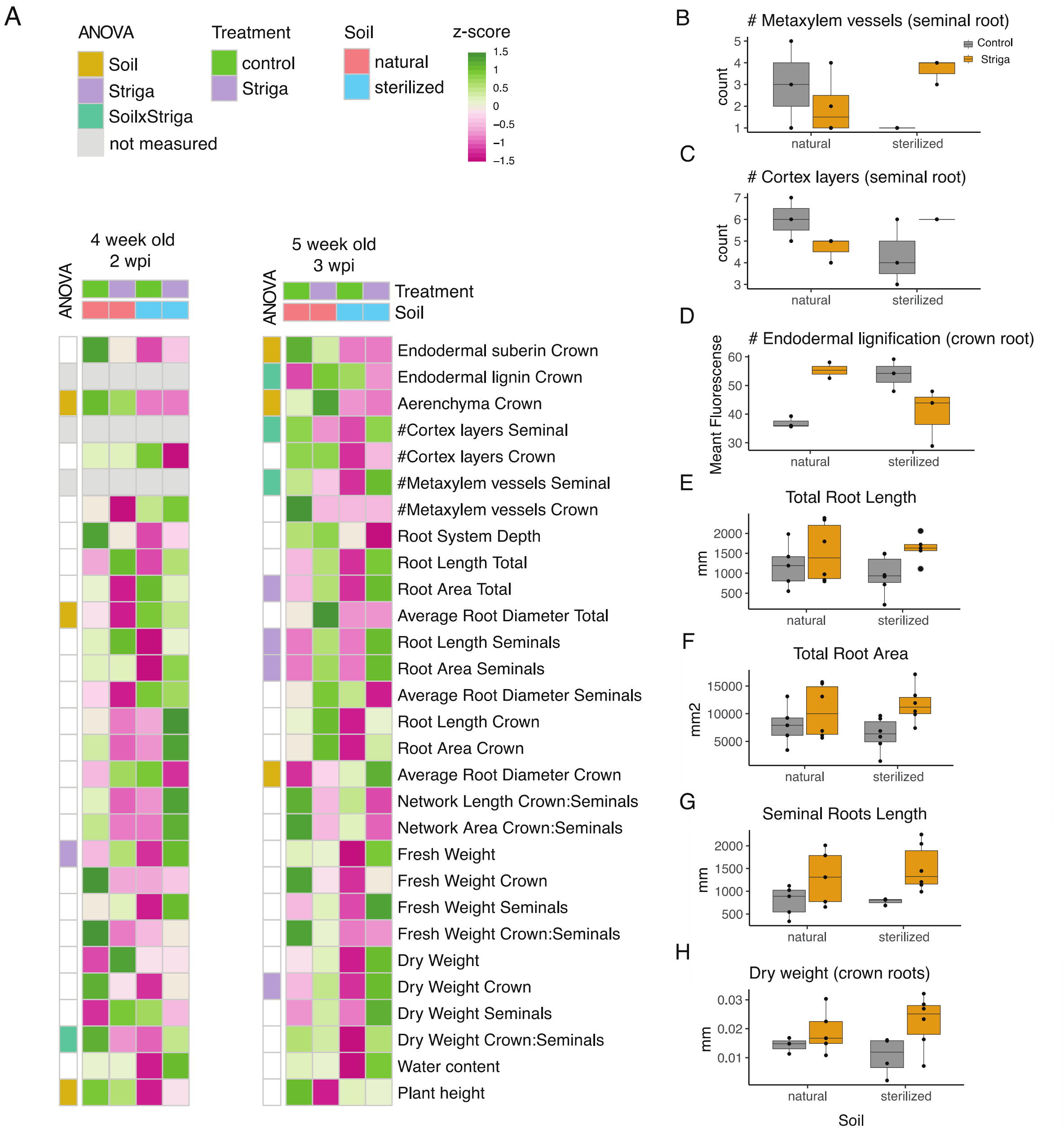

Supplementary Figure 2. Phenotypic characterization of root cellular anatomy, root system architecture and root biomass of Shanqui Red (SQR). (A) Heatmap presents values of each trait scaled across conditions tested. The left panel of each heatmap indicates whether the trait was significantly affected by soil, Striga, and soil by Striga interaction (as identified by a two-way ANOVA). (B) The number of metaxylem vessels and (C) cortex layers in seminal roots, (D) endodermal lignification of crown roots, (E) Length and (F) area of total root system, (G) length of seminal roots and (H) dry biomass of crown roots. Data in B-H are from five-week-old plants (three weeks post-infection). The boxplots denote data spanning from the 25th to the 75th percentile and are centered to the data median. Dots represent individual values.

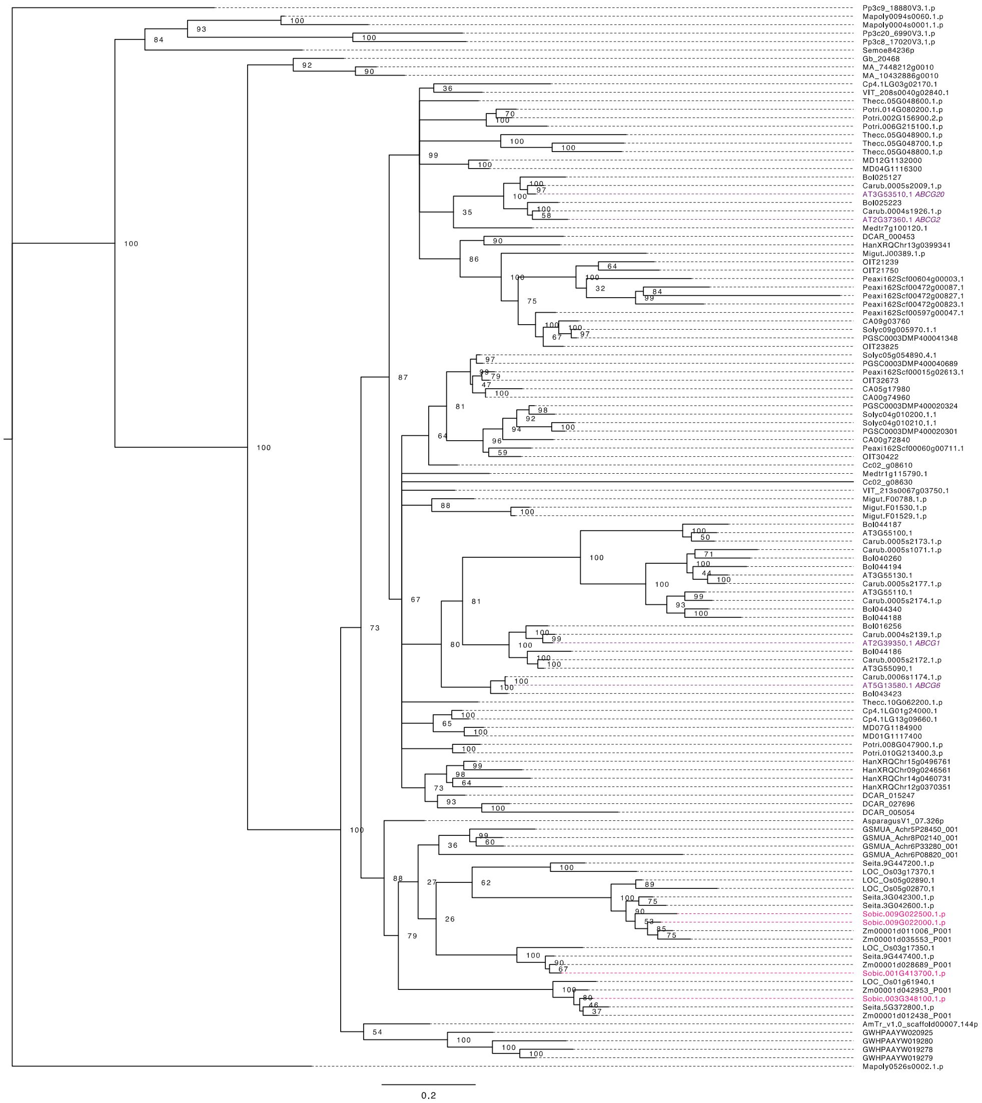

Supplementary Figure 3. Phylogenetic tree of ABCG transporters. Phylogenetic trees generated using protein sequences of several plant species. *Arabidopsis thaliana* genes are highlighted in purple. *S. bicolor* genes are highlighted in pink for reference. AmTr: *Amborella trichopoda*, AT: *Arabidopsis thaliana*, Asparagus: *Asparagus officinalis*, Azfi: *Azolla filiculoides*, Bol: *Brassica oleracea*, Carub: *Capsella rubella*, CA: *Capsicum annuum*, Cc: *Coffea canephora*, Cp: *Cucurbita pepo*, DCAR: *Daucus carota*, Gb: *Ginkgo biloba*, HanXRQ: *Helianthus annuus*, MD: *Malus domestica*, Mapoly: *Marchantia polymorpha*, Medtr: *Medicago truncatula*, Migut: *Mimulus guttatus*, GSMUA*: Musa acuminata*, OIT: *Nicotiana attenuata*, GWHPAAYW: *Nymphaea colorata*, LOC_Os: *Oryza sativa japonica*, Peaxi: *Petunia axillaris,* Pp: *Physcomitrella patens*, MA: *Picea abies*, Potri: *Populus trichocarpa*, Semoe: *Selaginella moellendorffii*, Seita: *Setaria italica,* Solyc: *Solanum lycopersicum*, PGSC: *Solanum tuberosum*, Sobic: *Sorghum bicolor*, Thecc: *Theobroma cacao*, VIT: *Vitis vinifera*, Zm: *Zea mays.*

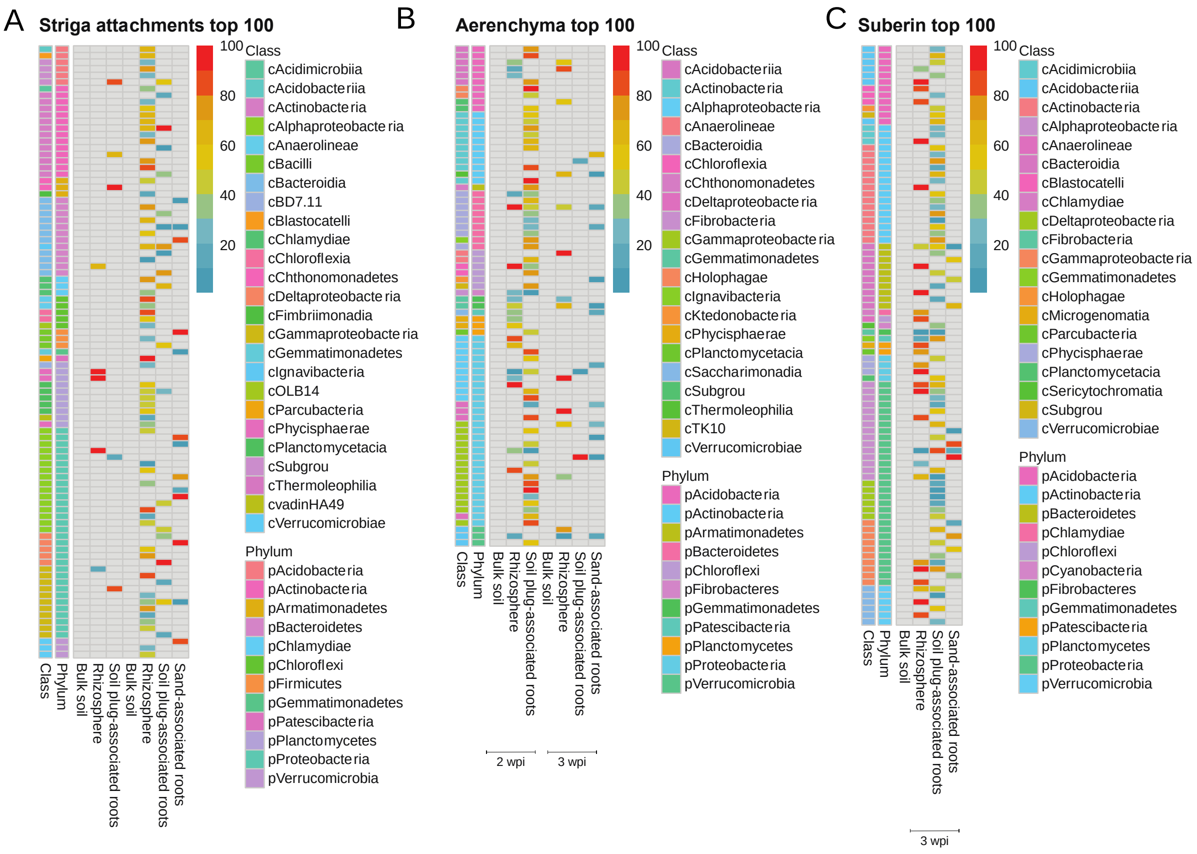

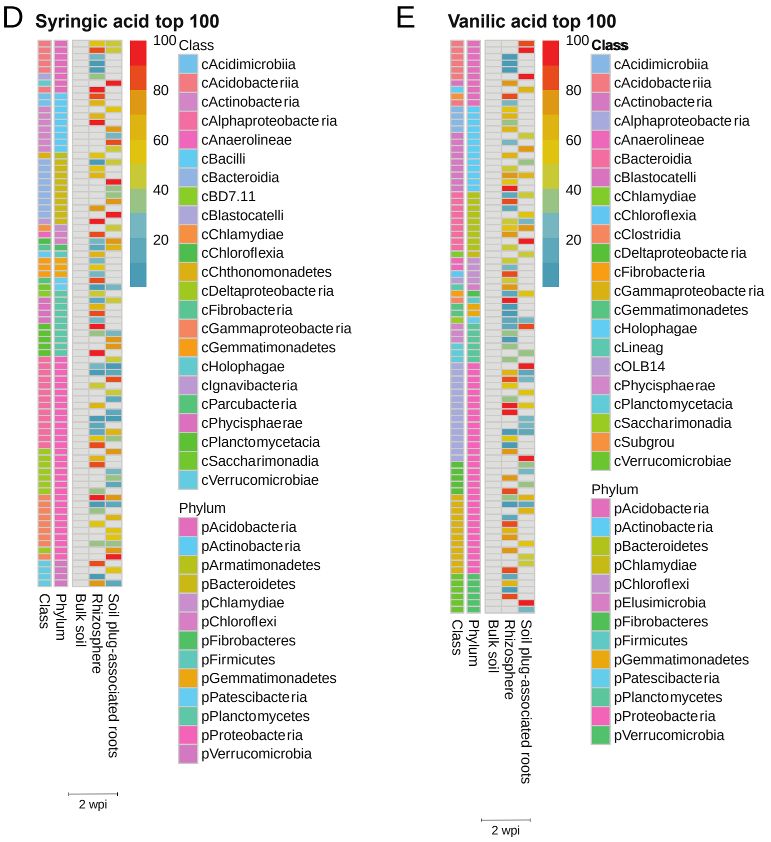

Supplementary Figure 4. Overview of top 100 bacterial taxa predicted to (A) reduce the number of Striga attachments; (B) induce aerenchyma formation; (C) induce endodermal suberization; reduce levels of, (D) syringic acid, (E) vanillic acid. Heatmaps present the rank calculated for each taxa (with blue indicating highest, while red the lowest rank) and the microbial sub-categories where each taxon was found (x-axis). Taxonomic membership at the phylum and class level is indicated with side panel colors.

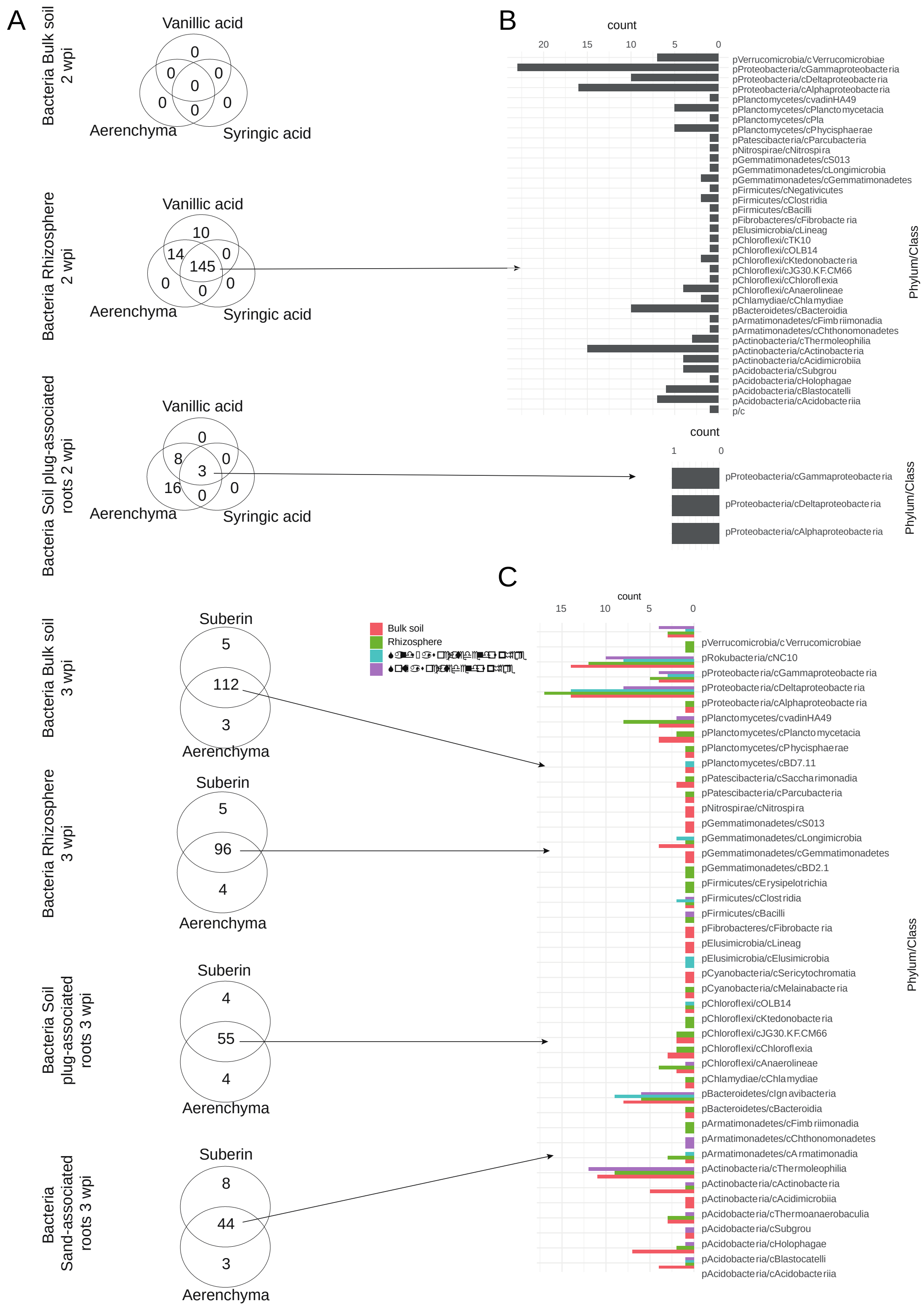

Supplementary Figure 5. Overlap of the number of bacterial taxa predicted to reduce Striga infection via each mechanism of action. (A) Number of bacteria found to reduce Striga infection via each of the mechanisms with the cut-off of residual correlation -0.2 for Striga attachments, syringic acid and vanillic acid levels and 0.2 for aerenchyma proportion and suberin content. Phylogenetic membership of taxa inducing (B) all four modes of Striga suppression in the rhizosphere two weeks post-infection and inducing suberin content and aerenchyma formation across microbial sub-categories three weeks post-infection.

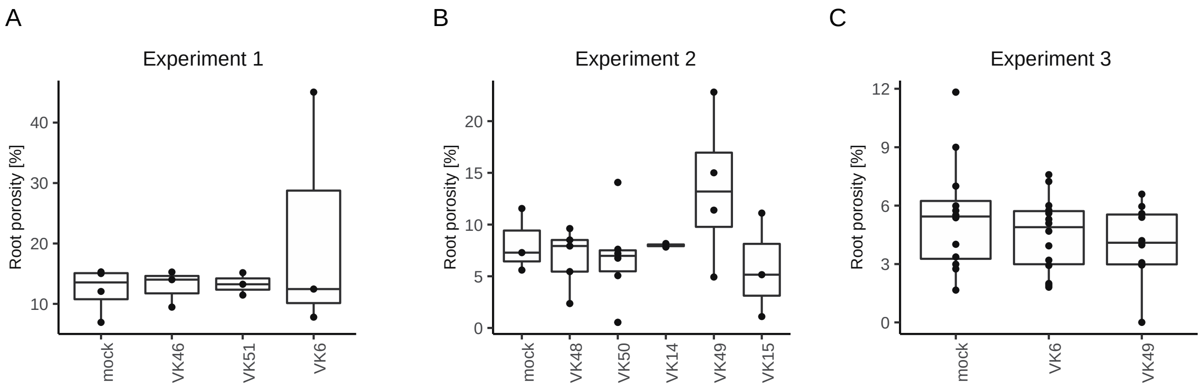

Supplementary Figure 6. Root porosity as a proxy for aerenchyma content of the whole root system (expressed as a proportion of the volume of the whole root system) of plants inoculated with (A) Pseudomonas 1987, 2039 and 2050, (B) Arthrobacter VK48, VK14, VK49, VK49 and Pseudomonas VK50, n = 6. (C) Isolates VK6 and VK49 were retested with n =15. One-way ANOVA was used to determine the effect of the inoculation. No significant differences were detected.

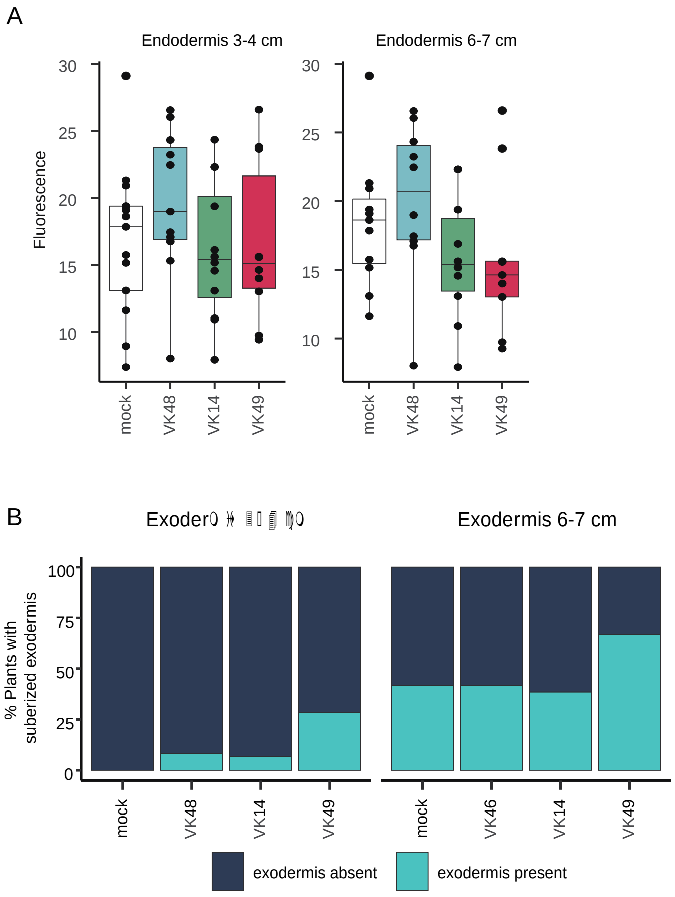

Supplementary Figure 7. Suberin content in the main root endodermis (A) of plants inoculated with Arthrobacter strains VK48, VK 2073 and VK 2105. Suberin was stained with fluorol yellow and quantified with mean intensity of pixel. One-way ANOVA was used to determine the effect of the inoculation. No significant differences were detected. B) Percentage of plants with a suberized or non-suberized exodermis in the root region 3-4 cm and 6-7 cm from the root tip, upon inoculation with Arthrobacter strains VK 1979, VK 2073 and VK 2105. No significant differences were detected as per Fisher exact test.

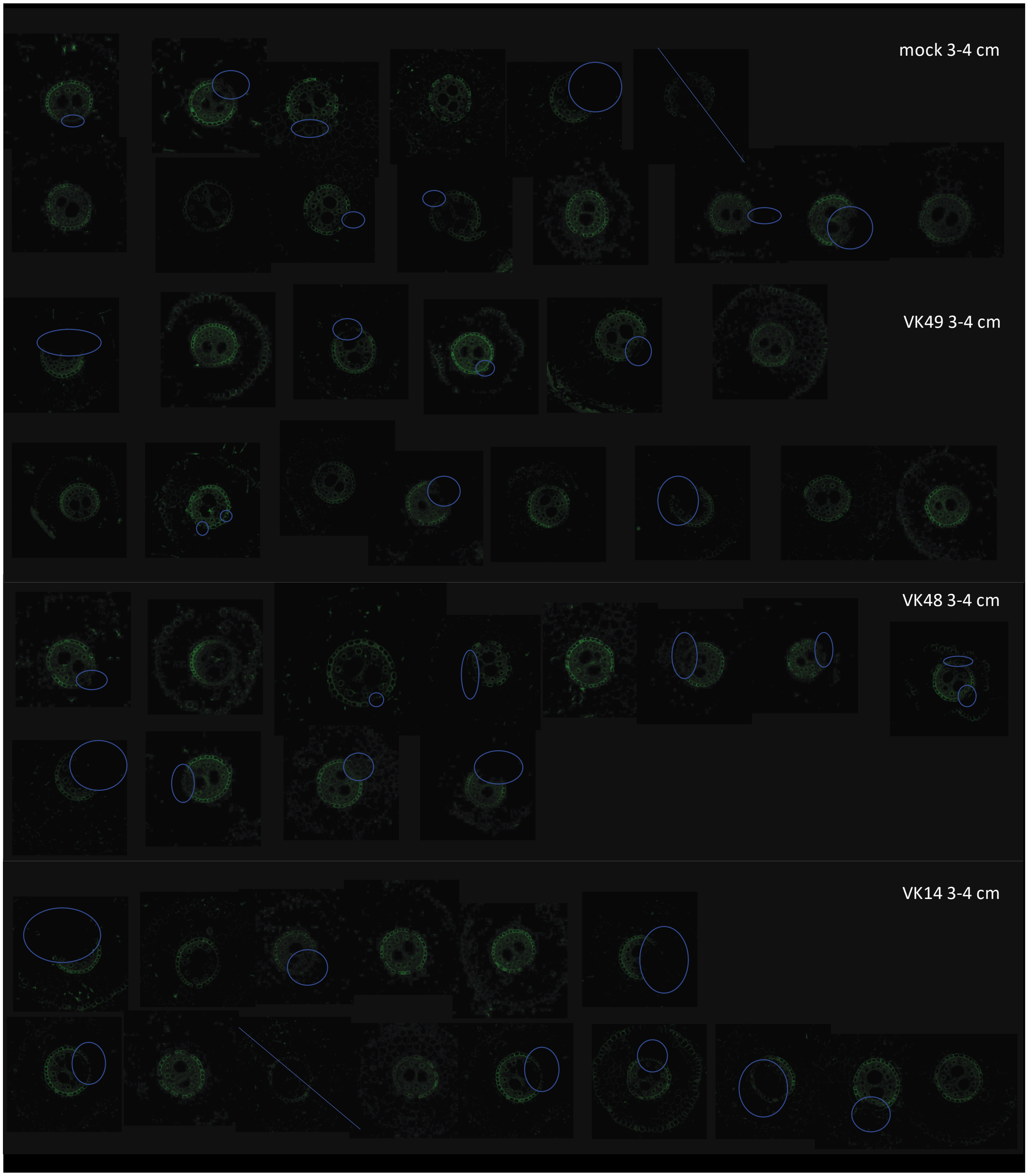

Supplementary Figure 8. Raw images used for quantification of the proportion of suberized cells in endodermis. Blue circle denotes regions that were excluded from the analysis (see Methods).
